# New Mannich-type arylidenerhodanines as potent inhibitors of AChE and BChE: synthesis, biological evaluation, cytotoxicity and molecular modeling

**DOI:** 10.1039/d5ra07416a

**Published:** 2025-12-15

**Authors:** Feyzi Sinan Tokalı, Halil Şenol, Yeliz Demir, Orhan Uluçay, Mubashir Ameen, Zahid Shafiq

**Affiliations:** a Department of Material and Material Processing Technologies, Kars Vocational School, Kafkas University 36100 Kars Türkiye feyzitokali@gmail.com; b Department of Pharmaceutical Chemistry, Faculty of Pharmacy, Bezmialem Vakif University 34093 Fatih Istanbul Türkiye; c Department of Pharmacy Services, Nihat Delibalta Göle Vocational High School, Ardahan University 75700 Ardahan Türkiye; d Department of Chemistry, Faculty of Sciences, Atatürk University 25240-Erzurum Türkiye; e Department of Bioengineering, Faculty of Engineering and Architecture, Kafkas University 36100 Kars Türkiye; f Faculty of Pharmacy, Bahauddin Zakariya University Multan 60800 Pakistan; g Institute of Chemical Sciences, Bahauddin Zakariya University Multan 60800 Pakistan zahidshafiq@bzu.edu.pk

## Abstract

Alzheimer's disease (AD) is a neurodegenerative disorder with a gradual increase in severity. The underlying cause of the disease is the dysfunction of cholinergic neurotransmission affecting mainly the activity of acetylcholinesterase (AChE) and butyrylcholinesterase (BChE). Within the context of the present research, a new group of 3,5-disubstituted rhodanine derivatives containing tertiary amine groups has been prepared and their potency in the inhibition of AChE and BChE was assessed. Enzymatic assays demonstrated that compounds 6 and 11 exhibited exceptional inhibitory potency, with *K*_i_ values of 13.61 nM and 12.70 nM against AChE, and 10.44 nM and 25.11 nM against BChE, respectively, surpassing the reference inhibitors tacrine (145.21 nM for AChE and 169.54 nM for BChE) and donepezil (67.41 nM for AChE and 62.44 nM for BChE). Cytotoxicity studies confirmed minimal toxicity in human umbilical vein endothelial cells (HUVEC) at concentrations several times higher than the effective inhibitory doses (IC_50_ = 79.13 µM for 6 and 69.14 µM for 11). The results from molecular docking and MM-GBSA calculations supported this presumption by foretelling strong binding affinities, where compound 11 was the one to show a free energy of −103.26 kcal mol^−1^ for AChE and compound 6 −86.75 kcal mol^−1^ for BChE. Moreover, the 250 ns molecular dynamics simulations gave a confirmation of the structural stability and the prolonged existence of the key interactions in the enzyme active sites during the entire time. The findings of this research emphasize compounds 6 and 11 as potential candidates for the creation of strong cholinesterase inhibitors for the treatment of Alzheimer's disease, thus encouraging additional studies.

## Abstract 2

Mannich-type rhodanine derivatives showed potent AChE/BChE inhibition with low cytotoxicity, supported by strong and stable binding modes, highlighting their potential for Alzheimer's therapy.

## Graphical abstract

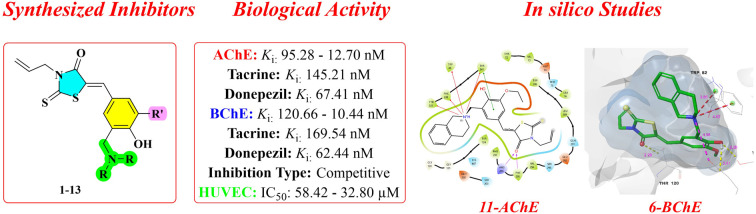

## 1. Introduction

Alzheimer disease (AD) is a major disorder impacting the life quality of both patients and their families, it targets mostly middle-aged and old people, and is considered as one of the worst diseases which plague the elderly population.^1,2^ It is typified by widespread dementia such as personality changes and behavioral changes as clinical presentation. The modern pathophysiology includes decreased levels of acetylcholine (ACh), aggregation of β-amyloid (Aβ), deposition of the tau protein, oxidative stress, inflammation, and biometal dyshomeostasis.^3^ The shrinkage of ACh is deemed as the major cause of cognitive impairment in Alzheimer disease. Abnormal increases in the acetylcholinesterase (AChE) activity in victims lead to low levels of ACh.^4,5^ The ACh is rapidly hydrolyzed into choline and acetate anions by AChE, which consequently ends the long term excitatory action of neurotransmitters on the postsynaptic membrane.^6^ The most effective treatment approach to Alzheimer disease is the inhibition of AChE to replenish ACh levels. The currently existing type of therapeutic intervention is restricted to AChE inhibitors (AChEIs) such as donepezil, rivastigmine, galantamine, and tacrine (latter was discontinued because of toxicity) and the newly approved Aβ modulator aducanumab.^7,8^ Though AChEIs do not stop the advancement of the disease, they have shown usefulness in alleviating cognitive functions associated with learning and memory and form the major pharmacological agents in the management of the AD.^9,10^

The treatment period for these AChEIs is constrained because to the extensive damage to neurons in AD patients, resulting in AChE levels being permanently diminished by over 90%, although BChE levels may grow to 120% of the physiological norm.^11^ BChE serves a vital function as a compensatory enzyme in the progression of AD. BChE has been demonstrated to be strongly linked with aberrant β-amyloid (Aβ) deposition.^12^ Numerous researches have indicated that BChE inhibition may serve as a viable approach for the treatment of advanced AD.^13^ Consequently, AChE and BChE inhibitors are extensively employed in the treatment of AD.

**Fig. 1:**
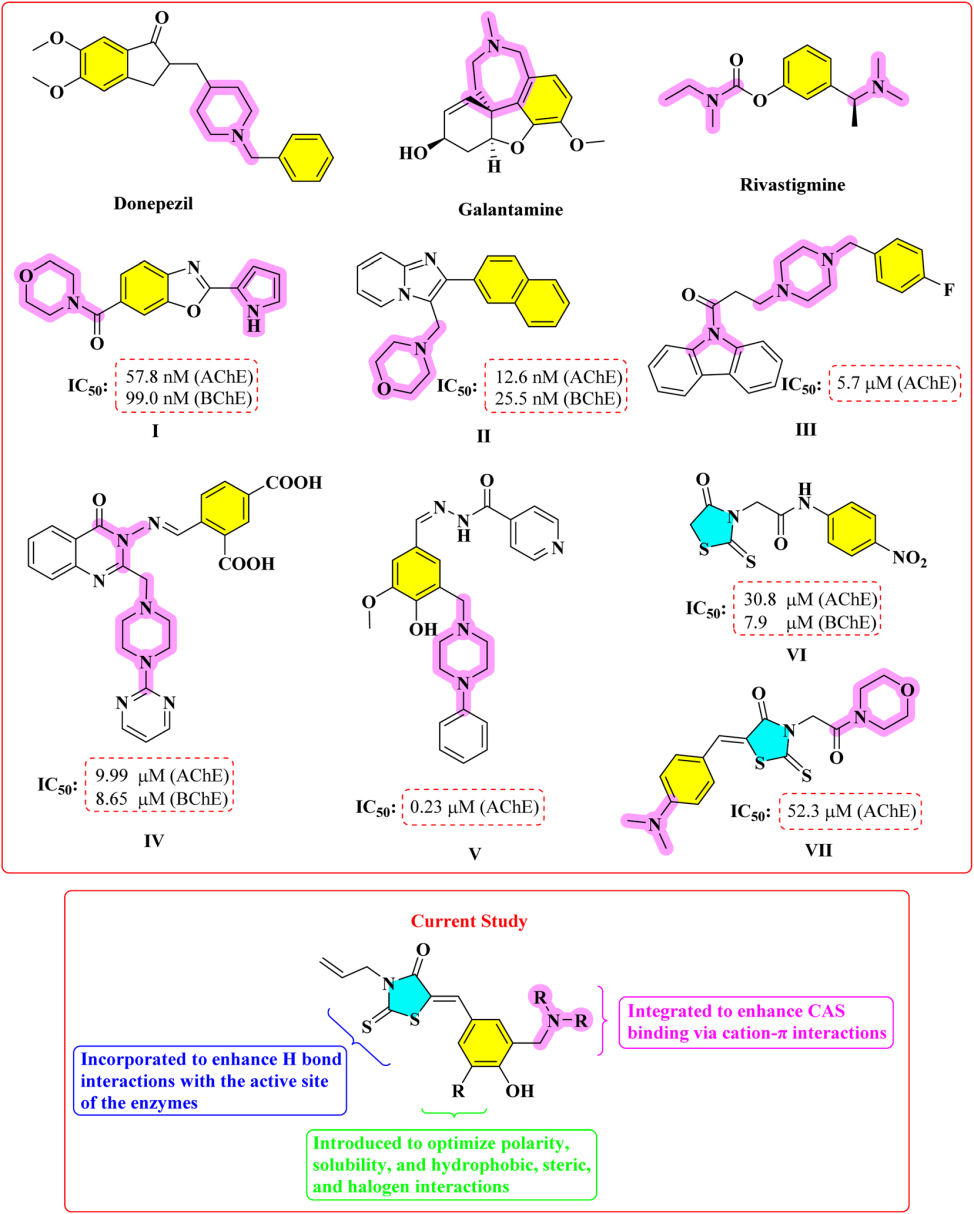
The design strategy of target compounds.

Tertiary amine groups are commonly present as pharmacophoric units in many cholinesterase inhibitors. This structural feature is also found in well-known cholinesterase inhibitors such as donepezil, rivastigmine, and galantamine (Fig. 1). The nitrogen atoms engage in cation–π interactions with the critical aromatic residues of the catalytic anionic site (CAS) of cholinesterases and thus interact with the CAS.^14,15^ In parallel, recent studies aiming to discover new cholinesterase inhibitors frequently incorporate tertiary amine moieties into molecular structures, regardless of the specific chemical class of the synthesized compounds (I–V) (Fig. 1).^16–21^ The Mannich reaction, a three-component reaction involving formaldehyde and a secondary amine, is one of the most powerful ways to add tertiary amine functionality to drug-like skeletons. It is highly important in medicinal chemistry as a technique that can be easily adjusted for the pharmacokinetic properties. For example, incorporation of polar aminomethyl groups can be one method to increase water solubility, while using certain amines can generate lipophilicity instead, this being contingent on the therapeutic profile desired.^22,23^ Moreover, Mannich-type derivatives are commonly investigated as prodrugs, which are able to liberate the active substance by means of enzymatic or chemical hydrolysis processes like deaminomethylation or deamination.^24^

The rhodanine nucleus has been a subject of considerable attention in the course of drug discovery and development due to its various biological activities and flexible structures. Being privileged scaffolds, rhodanines have a wide range of pharmacological activities, which include antimicrobial,^25^ anticancer,^26^ antidiabetic,^27,28^ and anti-inflammatory^29^ activities. The combination of their ability to establish strong interactions with wide ranges of biological targets with synthetic accessibility and structural diversity makes them useful targets in medicinal chemistry programs. Cholinesterases are also of biological interest as rhodanine targets in recent years, mostly as a result of discoveries on newer published studies. Despite the lack of research in this field, in some cases, the incorporation of the rhodanine core with other functional groups has led to compounds with strong cholinesterase inhibitory properties, thus indicating possible therapeutic effects against the Alzheimer disease (VI and VII) (Fig. 1).^30–32^

Based on the above, we have come up with a logical design plan based on molecular hybridisation to install the rhodanine scaffold tertiary amine moieties in one molecular platform (Fig. 1). The rhodanine core has been shown to have interaction potential with various biological targets, including cholinesterases. The selection of the R substituents was done to determine systematically the effect of the electronic and steric properties on cholinesterase binding. Electron-donating substituents, methoxy and ethoxy groups, were added to enhance the interactions in the hydrophobic pockets, whereas the electron-withdrawing and relatively bulky bromo group was added to test steric tolerance and possible halogen bonding. The polarizing group morpholinomethyl group was added to improve polarity, solubility, and also to increase the number of hydrogen-bonding interactions thus assessing the polar group (polar functionalities) on enzyme affinity. The NR_2_ moieties in the Mannich-type linkage were chosen to maximize interactions with the CAS through cation–π interactions, while also allowing modulation of key physicochemical properties such as solubility and lipophilicity. Hence, this directed strategy enabled a logical appraisal of the influence of the rhodanine core and the tertiary amine functionalities on the inhibition of the enzyme, granting significant understanding of the structure–activity relationship, even though the compound set was restricted.

## 2. Results and discussion

### 2.1. Chemistry

**Fig. 2:**
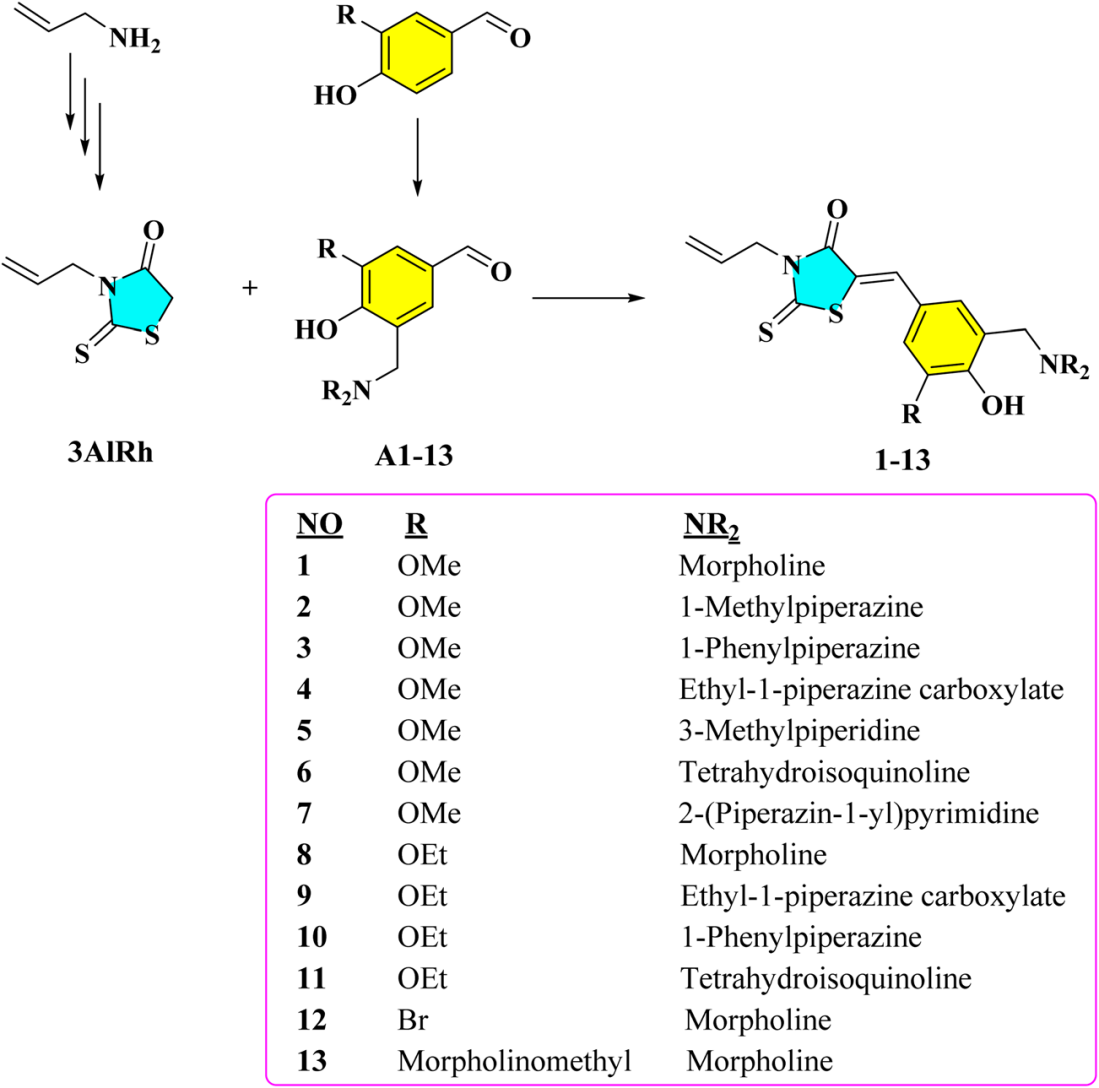
The synthesis scheme of compounds 1–13.

In this study, thirteen new 3,5-disubstituted rhodanine derivatives were designed and synthesized. In the synthesis study, firstly, 3-allylrhodanine (3AlRh) was obtained by the reaction of allylamine, carbon disulfide, and chloroacetic acid according to the method reported by Aisha *et al.*^33^ Mannich-type phenolic aldehydes (A1–13) were synthesized from the reaction of phenolic aldehydes with various seconder amines in the presence of formaldehyde according to the Mannich reaction. Finally, 3AlRh was treated with A1–13 in the presence of catalytic amount of piperidine and the target compounds (1–13) were obtained with good yields (Fig. 2). The structures of the target compounds were characterized with FTIR, ^1^H NMR, ^13^C NMR, and HRMS spectroscopic methods. The spectra of the compounds are given in the SI file.

In the FTIR spectra of the compounds 1–13, the characteristic C=O band of the rhodanine ring and C=CH band of the benzylidene moiety are seen at the range of 1707–1690 cm^−1^ and 1581–1571 cm^−1^, respectively. In the ^1^H NMR spectra of the compounds, peaks of the characteristic C=CH protons of the arylidene moiety resonated as a singlet at the range of *δ* 7.66–7.62 ppm. The signals corresponding to the allylic protons were observed at the range of 5.93–4.73 ppm as two multiplets and a doublet. A sharp singlet was displayed by the amine (–CH_2_–N) protons in the region of *δ* 3.86–3.77 ppm. In the ^13^C NMR spectra, the C=S carbons resonated between *δ* 193.0–192.4 ppm, whereas the C=O signals appeared at the range of *δ* 167.6–167.4 ppm. The C=CH carbons of the arylidene moiety and the aminomethyl carbons were seen at *δ* 135.2–133.7 ppm and *δ* 61.6–59.0 ppm, respectively.

### 2.2. Enzyme inhibition

#### 2.2.1. AChE inhibition study and structure–activity relationship (SAR) analysis

All synthesized compounds exhibited strong inhibitory profiles against both AChE and BChE (AChE *K*_i_: 12.70–95.28 nM; BChE *K*_i_: 10.44–112.34 nM), with *K*_i_ values notably lower than those of the reference drug tacrine (AChE *K*_i_: 145.21 nM; BChE *K*_i_: 169.54 nM). For donepezil (AChE *K*_i_: 67.41 nM; BChE *K*_i_: 62.44 nM) some compounds are described as having better inhibition action while others as having only slightly weaker action. To be more precise, compounds 1–4, 6, 7, 9, 10, 11, and 13 showed greater inhibition than donepezil for at least one of the enzymes and compounds 5, 8, and 12 were considered to be less potent. Among all the tested compounds, 6 (AChE 13.61 nM; BChE 10.44 nM) and 11 (12.70 nM; 25.11 nM) presented the strongest inhibition, with *K*_i_ values that are much lower than those of donepezil and tacrine (Table 1).

**Table 1:** *K*_i_ values of compounds 1–13, tacrine, and donepezil for AChE and BChE

Inhibitor	AChE (nM)		BChE (nM)		Selectivity
	*K*_i_	*R* ^2^ a	*K*_i_	*R* ^2^	(BChE/AChE)
1	57.62 ± 11.56	0.9655	53.65 ± 8.87	0.9412	0.93
2	46.05 ± 5.21	0.9678	50.92 ± 10.44	0.9789	1.11
3	62.02 ± 13.61	0.9478	67.66 ± 11.44	0.9657	1.09
4	28.73 ± 6.12	0.9805	33.05 ± 9.21	0.9554	1.15
5	95.28 ± 11.23	0.9734	88.67 ± 15.45	0.9612	0.93
6	13.61 ± 1.43	0.9854	10.44 ± 1.33	0.9878	0.77
7	25.44 ± 9.45	0.9751	28.98 ± 7.54	0.9701	1.14
8	85.11 ± 8.45	0.9556	120.66 ± 12.67	0.9456	1.42
9	31.46 ± 5.15	0.9589	40.45 ± 6.63	0.9675	1.29
10	18.63 ± 2.05	0.9799	21.55 ± 5.23	0.9656	1.16
11	12.70 ± 1.96	0.9611	25.11 ± 6.41	0.9812	1.98
12	43.00 ± 8.77	0.9809	97.22 ± 17.87	0.9814	2.26
13	26.51 ± 9.21	0.9567	112.34 ± 23.55	0.9743	4.24
Tacrine	145.21 ± 18.66	0.9702	169.54 ± 25.66	0.9766	1.17
Donepezil	67.41 ± 7.75	0.9688	62.44 ± 9.31	0.9832	0.93

a *R* ^2^: coefficient of determination.

The results indicated that the degree and positions of ring substitution altered the binding affinity of enzymes across the investigated series. A detailed SAR analysis is provided.

**Fig. 3:**
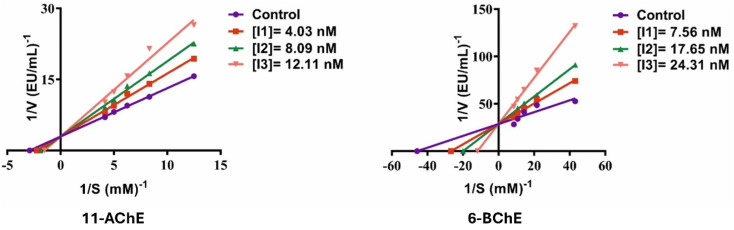
Lineweaver–Burk plots for compounds 11 and 6 demonstrate competitive inhibition of AChE and BChE, respectively.

Compound 1 was moderate with a *K*_i_ of 57.62 nM. The compound has a methoxy and morpholinomethyl replacement pattern. The polar interactions could have been provided by the morpholine ring, but the lack of a large aromatic system might have limited π–π stacking with the peripheral anionic site (PAS) and limited the overall potency. Compound 2 had a better inhibitory effect of a *K*_i_ of 46.05 nM. Replacement of morpholine ring by the methylpiperazine moiety seems to increase the capability of the compound to bind to the AChE gorge through hydrophobic interactions and possible hydrogen bonding of the second tertiary amine. The framework leans itself towards conformational flexibility and no more than steric hindrance. The presence of a phenylpiperazine group in compound 3 resulted in a *K*_i_ of 62.02 nM which was a little weaker than compound 2. The introduction of a bulk aromatic ring may have aided the increase of the π–π stacking with aromatic residues in PAS, but the steric demands were probably prohibiting the perfect fit in the active site gorge. Compound 4 was shown to increase its activity significantly with a *K*_i_ of 28.73 nM, 5.05 and 2.35 times stronger than tacrine and donepezil, respectively. The polar and hydrogen bonds are probably increased by the ethoxycarbonylpiperazine substituent. Together with its rather small and mobile structure, the molecule successfully interacts with the catalytic site, as well as the peripheral pocket of the enzyme. Compound 5 had a lower potency of 95.28 nM. The lack of aliphatic bulk due to a 3-methylpiperidine group is offset by the lack of a rigid aromatic system lowering π–π stacking, leading to a lesser affinity. The compound is a minimal bit more efficient than tacrine and does not perform as well in comparison to its aromatic analogues. Compound 6 became one of the strongest AChE inhibitors in the series with a *K*_i_ of 13.61 nM which is 10.67 and 4.95 times stronger than tacrine and donepezil. This addition of tetrahydroisoquinoline moiety adds an aromatic scaffold that is probably involved in powerful interactions with major aromatic residues. Besides that, the isoquinoline nitrogen can also take part in the hydrogen bonding and this is what contributes to its exceptional binding affinity. Compound 7 had a *K*_i_ of 25.44 nM, which was more than 2.65 times active than donepezil. The molecule has a pyrimidinylpiperazine substitution which combines heteroaromatic and flexible components. The addition of the pyrimidine ring with additional nitrogen atoms, which could serve as the hydrogen bond acceptors, is accompanied by the structural flexibility of the piperazine, which is necessary to accommodate the enzyme in the groove. Compound 8 had a *K*_i_ of 85.11 nM which was slightly better than tacrine but much lower than donepezil and compound 1, even though structurally it was similar. The ethoxy replacement to be used instead of methoxy seems to cause a small degree of steric hindrance and reduced polarity, minimizing hydrogen bonding opportunities and binding complementarity. Compound 9, an ethoxy analog to compound 4, had a *K*_i_ of 31.46 nM (Table 1), which is at the top of the active inhibitors in the group. The compound still has the ethoxycarbonylpiperazine unit which seems to be important to keep the compound with a high potency. The ethoxy substitution is likely to introduce some steric bulk, but the compound has nevertheless been able to bind the enzyme effectively albeit at slightly lower efficiency than compound 4. One of the most active molecules in the series is compound 10 which has a *K*_i_ of 18.63 nM. The combination of phenylpiperazine substituent and an ethoxy group is the possible factor that contributes to aggressive hydrophobic and aromatic interactions. Such a two-aromatic structure allows very strong contact with the CAS and the PAS, which increases the inhibitory efficiency significantly. Compound 11 was very strong in 12.70 nM of *K*_i_ that is more effective than references. Besides, the compound was measured to have a competitive form of inhibition (Fig. 3). The structure retains a tetrahydroisoquinoline framework together with an ethoxy group of the aryl ring. It is the most active compound in the series and it remains highly active meaning that the tetrahydroisoquinoline motif takes consistently good binding even when there are slight polar changes. Compound 12 was mediocre with a *K*_i_ of 43.00 nM. Bromine substituent increases lipophilicity and introduces the potential of halogen bonding. Although these features enhance the binding a little more than simpler derivatives of morpholine, steric effects could have inhibited stronger interactions in the active site. Compound 13 was potently active and its *K*_i_ was 26.51 nM. The structure has two morpholinomethyl groups, presumably, involved in various hydrogen bonds. Though the augmented polarity boosts the binding, the huge substituent motif can bring about conformational restraint, which somewhat restrains the complete inhibitory capacity, relative to the most successful compounds.

#### 2.2.2. BChE inhibition study and SAR analysis

Inhibitory properties of compounds 1–13 were also evaluated against BChE, a secondary cholinesterase enzyme which has become more and more relevant within the context of AD pathogenesis especially in late stages when AChE activity fades and BChE activity rises. Regarding the BChE inhibition, a number of compounds were more potent than tacrine and donepezil (*K*_i_: 169.54 and 62.44 nM, respectively). Compound 6 was the most effective BChE inhibitor with a *K*_i_ value of 10.44 nM, 10 (21.55 nM), 11 (25.11 nM) and 4 (33.05 nM). These and other compounds showed much greater inhibitory effect, especially the compound 6, which proved to be 16.24 and 5.98-fold more inhibitory than tacrine and donepezil respectively (Table 1).

Compound 1 was a potent inhibitor of BChE, which is more than 3.16 times more inhibitive (*K*_i_: 53.65 nM) than tacrine. The morpholinomethyl group is probably useful in offering advantageous hydrogen bonding in the bigger and more relaxed BChE active site. The moderate lipophilicity of the compound combined with the possibility of polar interaction suggests the opportunity to fit into the BChE binding pocket best. With a slightly higher activity than 1, compound 2 (*K*_i_: 50.92 nM) incorporates a methylpiperazine moiety that increases its binding potential through electrostatic interactions, as well as better hydrophobic complementarity in BChE extended active site. The tight binding is probably due to the small size of the methylpiperazine which prevents steric interference. Replacement of this phenylpiperazine residue (3) with a BChE inhibitory potency of 2 slightly. While the aromatic ring might offer π–π stacking, its increased steric bulk may prevent ideal binding within the deeper pockets of the BChE enzyme. Compound 4 (*K*_i_: 33.05 nM) was potent in its inhibition with an ethoxycarbonylpiperazine replacement. The presence of the ester moiety adds potency of the dipole–dipole and H-bonding ability, especially in the highly polar active site of BChE. This is probable to be tight-binding due to its balance of hydrophilicity and conformational flexibility. Compound 5 was one of the weaker inhibitors in the series though more effective than tacrine (*K*_i_: 88.67 nM). The 3-methylpiperidine ring is non-aromatic and hydrogen bonding is constrained, and the saturated congesting character of the ring can result in the incomplete accessibility to cavities of greater depths in BChE. Compound 6 (*K*_i_: 10.44 nM) was an excellent BChE inhibitor, whereby *K*_i_ was lower than 16.24 and 5.98 fold than tacrine and donepezil. Also, this compound was detected to have a competitive inhibition profile (Fig. 3). 7 (*K*_i_: 28.98 nM), containing a pyrimidinylpiperazine group, performed very well, with a *K*_i_ 5.85-fold lower than tacrine. The heteroaromatic pyrimidine ring may interact *via* polar coordination and edge-to-face interactions with surrounding polar residues. This is further facilitated by its flexibility and permits occupation of the enzyme. Compound 8 that is among the less potent inhibitors contains an ethoxy functional group and a morpholine ring whose hydrophilicity may not be stabilized adequately in the inner cavity of BChE that is relatively more hydrophobic. Although the compound has hydrogen bond potential, the compound is not aromatic or planar, which correlates with binding BChE tighter. Compound 9 analog of 4 was also very active, although it has an ethoxy group rather than a methoxy. The most important contribution is the ethoxycarbonylpiperazine that is likely to stabilize the dipolar interactions. Compound 10 (*K*_i_: 21.55 nM) had an ethoxy group and a tetrahydroisoquinoline core without losing excellent binding affinity. The planar and aromatic geometry facilitates a high level of interaction with key residues, which places the compound in one of the leading BChE inhibitors in this series 11 showed very high inhibitory activity (*K*_i_: 25.11 nM), consistent with its AChE performance. The presence of both phenylpiperazine and ethoxy substituents forms a dual-interaction motif, engaging both PAS and the hydrophobic subpockets. This structure enables strong π–π and van der Waals interactions, explaining its over 6.75 and 2.49-fold higher potency than references. Although compound 12 has a bromine substituent that in theory can help form halogen bonds, it was still slightly inhibited. The bromine can provide steric hindrance and favor orientation in the active site and counteract some of the potential hydrophobic benefits. Though this compound 13 has two morpholinomethyl groups that could form multiple hydrogen bonds, the size of such groups could decrease the binding efficiency provided by the limited conformation freedom. Consequently, its affinity was worse than most other derivatives, but superior to tacrine.

#### 2.2.3. Dual AChE/BChE inhibitor profile and selectivity

One of the main predictors of therapeutic efficacy of candidate compounds is the cholinesterase selectivity.^34^ AChE's primary inhibitors increase acetylcholine levels in synapses, thereby affecting intelligence and cognitive abilities. As it is a primary inhibitor of AChE, it is relatively more effective in the early stages of Alzheimer's disease.^35^ On the other hand, BChE-selective inhibitors can be of increased use at later stages of the disease when BChE activity increases as AChE activity decreases and have also been linked to a decrease in β-amyloid aggregation.^36^ AChE and BChE inhibitors have greater therapeutic potential and maintain their efficacy during disease progression, allowing for more balanced regulation of cholinergic signaling.^37^ The ratio of BChE *K*_i_ to AChE *K*_i_ (BChE/AChE) is called selectivity; non-selective inhibition is a value near 1, AChE preference is a value less than 1 and BChE preference is a value larger than 1.^38^ A number of these new series known compounds had both AChE and BChE dual inhibitory activity. According to the selectivity index, all the compounds 1–10 were dual inhibitors with the same affinity to the enzymes (0.77–1.42). On the contrary, 11, 12, and 13 were highly AChE selective and the SI of these compounds exceeded 2.0 which is a good indication of preference to AChE. Compound 8 (SI = 1.42) only showed a slight preference by BChE. In comparison with tacrine which exhibited moderate dual activity but weak AChE selectivity, all compounds of this series exhibited better inhibitory characteristics, which makes them good future cholinesterase inhibitors.

#### 2.2.4. Correlation between AChE and BChE inhibitory activities and heatmap analysis

**Fig. 4:**
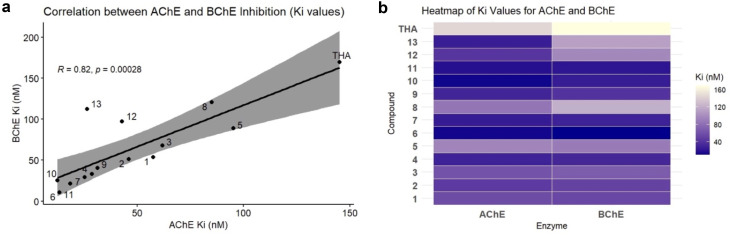
Comparative analysis of AChE and BChE inhibition by the synthesized compounds. (a) Pearson correlation plot reflecting the relationship between the *K*_i_ values of AChE and BChE for all compounds tested. Every dot depicts a single compound, and for the case of tacrine, it is treated as the reference inhibitor. (b) Heatmap visualizing the distribution of the *K*_i_ values (nM) for each compound against AChE and BChE. The color intensity signifies the inhibition potency, where the darker shades represent lower *K*_i_ values (stronger inhibition).

A Pearson correlation test was performed as a measure to examine the possible correlation between the inhibitory activities of AChE and BChE using the *K*_i_ values of the synthesized rhodanine derivatives. The correlation analysis showed that there was a statistically significant and strong positive correlation between the compounds and AChE (*R* = 0.82, *p* = 0.00028) that is, the compounds that have a strong inhibition of AChE also have a similar strong inhibition of BChE. Notwithstanding a considerable disparity in the architecture of the active sites of the two enzymes, AChE with a narrow, highly aromatic opening, and BChE with a wider and more flexible one, such correlation can be attributed to common structural interaction demands. The two cholinesterases have a conserved catalytic triad and CAS, prefer cation–pi and pi–pi interaction with tertiary amine-containing ligands. In the present study the Mannich-type rhodanine derivatives have tertiary amine moieties and extended aromatic systems that are uniformly built into these derivatives allowing the effective interactions with major residues in both enzymes. In addition, structural variations notwithstanding, the PAS of AChE and that of BChE complement the similarity on aromatic and cationic interactions. This mechanistic explanation justifies the activity of the SAR trends in the two cholinesterase enzymes. The majority of compounds were concentrated around the regression line, which indicated a coherent SAR in both enzymes as in Fig. 4a. It is interesting to note that the compounds 6, 10 and 11 with the strongest AChE inhibitory activity were also the strongest BChE inhibitors, another indication of the similarity of the two dual-site binding affinities. The line however, was slightly deviated by a few compounds like 12 and 13, which may have occurred because of steric or electrostatic effects that selectively affect binding to the larger BChE active site. This correlation suggests that the molecular properties that are selective to AChE inhibition could also play the role of BChE inhibition, but with enzyme-specific alterations.

A heatmap was generated to comprehensively illustrate the inhibitory activity of the synthesized products against AChE and BChE, utilizing their *K*_i_ values (Fig. 4b). The gradient of color indicates the degree of inhibition and darker colors depict greater inhibition (lower *K*_i_ values) and light colors depict lesser inhibition. Most of the compounds as shown were highly dual inhibitory, especially those compounds 6, 10 and 11, which were deeply blue in each of the two columns, indicating their low nanomolar *K*_i_. On the contrary, the reference inhibitor tacrine was observed in light colors, which proves its relatively weak inhibition. Other compounds like 12 and 13 had different binding affinity towards AChE and BChE. The heatmap analysis will visually support the SAR results and demonstrate several potentially useful dual inhibitors with high potency, as compared to tacrine.

### 2.3. Cytotoxicity

The early-stage evaluation of newly synthesized bioactive compounds typically involves not only assessing their efficacy against biological targets but also determining their safety profiles in relevant human cell lines. While enzymatic inhibition provides valuable insights into the pharmacological potential of novel compounds, it is equally important to assess their safety profiles in relevant non-cancerous human cell lines. In this study, we evaluated the cytotoxic effects of the most potent AChE and BChE inhibitors (4, 6, 7, 10, and 11) on the HUVEC cell line, a model of normal human umbilical vein endothelial cells. This assessment was undertaken to ensure that the observed cholinesterase inhibition was not accompanied by undesirable cytotoxicity, thereby supporting the compounds' potential for further therapeutic development. The results obtained from the cytotoxicity assay are presented in Table 2.

**Table 2:** Impact on cell viability caused by the most potent AChE and BChE inhibitors on the HUVEC cell line

Inhibitors	HUVEC
	IC_50_ (µM)
4	73.13 ± 0.50
6	79.71 ± 0.52
7	43.32 ± 0.47
10	47.39 ± 0.67
11	69.14 ± 0.59

The findings from the enzyme inhibition tests demonstrated that at low nanomolar concentrations these compounds are highly effective as inhibitors of AChE and BChE. The strongest inhibition was displayed by compounds 6, 10, and 11 with *K*_i_ values of 13.61 nM, 18.63 nM, and 12.70 nM, respectively. Cytotoxicity tests conducted on HUVEC cells have shown that the compounds used, even at higher concentrations, do not significantly reduce cell viability. For example, compound 6 had an IC_50_ of 79.71 µM, and compound 11 showed an IC_50_ of 69.14 ± 0.59 µM. Given that this cytotoxicity values are five thousand times higher than the *K*_i_ values for enzyme inhibition, it becomes clear that potent enzyme inhibition does not correlate with cytotoxic effects on normal cells.

### 2.4. Molecular docking

In the realm of drug discovery, molecular docking has become a computerized necessity that gives one the necessary information for a profound understanding of the small molecules that are the future drug candidates, how they interact with their corresponding target proteins, and their binding affinities. To make the results of the docking even more trustworthy, the Induced Fit Docking (IFD) procedure was used, which makes it possible to dynamically alter the conformations of the ligands as well as the flexibility of the binding site of the protein, thus simulating the physiological conditions more accurately. The strategy was further supported by the experimental data from the Molecular Mechanics-Generalized Born Surface Area (MM-GBSA) calculations, which were performed to evaluate quantitatively the binding free energies, thus giving a thermodynamic viewpoint on the stability and the favorability of the ligand–protein complexes.^39^ IFD scores and MM-GBSA binding free energy calculations indicate that all compounds produced exhibit significant binding affinity for both AChE and BChE enzymes and exceed the affinity of the reference inhibitor (Table 3).

**Table 3:** IFD scores and MM-GBSA Δ*G* binding free energies of the compounds against AChE and BChE

Compounds	IFD score (kcal mol^−1^)		MMGBSA Δ*G* bind. (kcal mol^−1^)	
	AChE (PDB ID: 4EY7)	BChE (PDB ID: 5NN0)	AChE	BChE
1	−12.895	−10.112	−74.97	−60.16
2	−13.601	−10.847	−77.86	−68.90
3	−12.905	−10.149	−67.60	−61.57
4	−14.062	−8.664	−75.25	−75.17
5	−13.885	−8.315	−72.92	−78.48
6	−15.258	−14.326	−90.61	−86.75
7	−14.915	−8.304	−68.72	−65.11
8	−12.719	−10.343	−80.28	−64.11
9	−15.057	−8.902	−82.38	−61.15
10	−13.498	−13.216	−76.80	−72.78
11	−18.468	−11.496	−103.26	−65.36
12	−12.990	−10.492	−71.76	−62.06
13	−13.825	−11.884	−64.42	−69.77
Tacrine	−12.990	−10.492	−71.76	−62.06
Donepezil	−13.662	−11.568	−78.82	−61.56

The IFD points *versus* AChE varied from roughly −12.7 to −18.5 kcal mol^−1^, with the compound 11 showing the best binding score (−18.468 kcal mol^−1^) indicating a very strong and complementary interaction in the AChE active site. In addition, compounds 6 (−15.258 kcal mol^−1^), 9 (−15.057 kcal mol^−1^), and 7 (−14.915 kcal mol^−1^) revealed high binding affinities to AChE and were superior to the reference drugs tacrine and donepezil which had scores of −12.990 and −13.662 kcal mol^−1^ respectively.

With respect to BChE, IFD scores most often showed less negativity relative to AChE but differed from −8.3 to −14.3 kcal mol^−1^. Compound 6 showed the most favorable docking score for BChE (−14.326 kcal mol^−1^) while compound 10 (−13.216 kcal mol^−1^) and compound 11 (−11.496 kcal mol^−1^) came next in scoring. The data imply very strong predicted interactions for compound 6 with BChE in particular.

MM-GBSA binding free energy calculations further substantiated the docking results. When it comes to compound 11, it exhibited the strongest binding stability with AChE, which was the reason for its most negative value of −103.26 kcal mol^−1^. Compound 6 was showing high binding as well with both enzymes (−90.61 kcal mol^−1^ for AChE and −86.75 kcal mol^−1^ for BChE), thus a stable ligand–protein complex could be formed. Compounds 9 and 8 were also displaying good binding free energies with AChE (−82.38 and −80.28 kcal mol^−1^, respectively).

Compounds 6 and 11, on the other hand, exhibited Δ*G*-bind values of −82.44 and −78.92 kcal mol^−1^ (AChE), and −62.25 and −62.19 kcal mol^−1^ (BChE), stiffness and thus higher predicted binding affinity than tacrine and donepezil, which showed Δ*G*-bind values of −71.76 and −78.82 kcal mol^−1^ (AChE) and −62.06 and −61.56 kcal mol^−1^ (BChE). The combined output of docking and MM-GBSA analysis clearly marks compounds 6 and 11 as the initial participants in the drug discovery process, due to their strong and long-lasting binding with AChE and BChE. These computer results do not contradict the *in vitro* inhibition data.

**Fig. 5:**
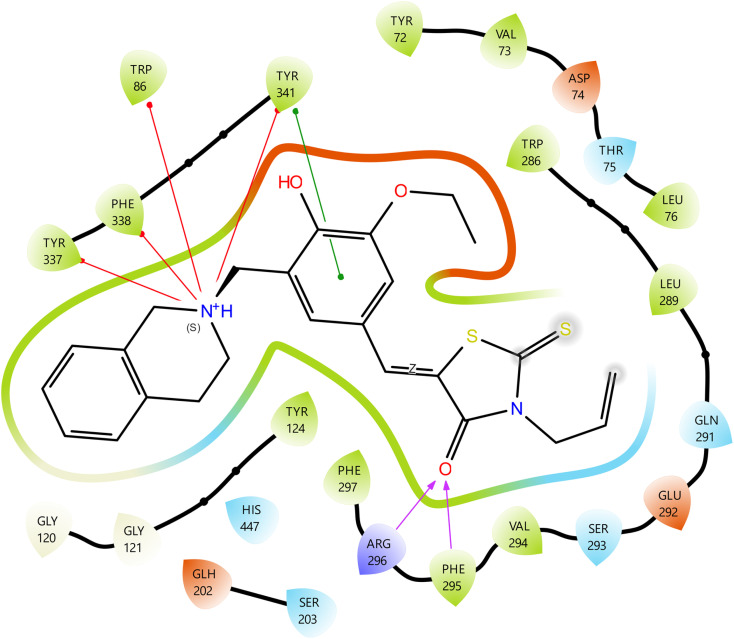
Molecular docking 2D ligand–protein interactions of 11–AChE complex.

The critical interactions of compound 11 with the active site residues of AChE are shown in Fig. 5. The ligand formed three hydrogen bonds which were very important and one of them was between its rhodanine carbonyl oxygen and the residues Arg-296 and Phe-295. The residues in question are located near the entrance of the active site gorge and play a crucial role in stabilizing the ligands as they enter the catalytic pocket, thus enhancing the efficiency of inhibition. The hydrogen bond formation among them strengthens the ligand's position and increases the overall binding affinity.^40,41^ The benzylidene phenyl ring was involved in π–π stacking along with Tyr-341 that further increased the aromatic interactions. The nitrogen of the tetrahydroisoquinoline provided superior binding through four π–cation interactions with Tyr-337, Phe-338, Trp-86, and Tyr-341. The amino acids are renowned for their roles in substrate recognition and catalysis; Trp-86 is crucial for stabilizing the quaternary ammonium group of acetylcholine within the catalytic active site,^42^ whereas Tyr-337 and Tyr-341 are pivotal in the peripheral anionic site, regulating substrate accessibility and the interaction with β-amyloid.^43^ The dual inhibitory mechanism is indicated by the multi-site engagement of this compound, which not only improves but also potentially makes the drug effective against AChE.

**Fig. 6:**
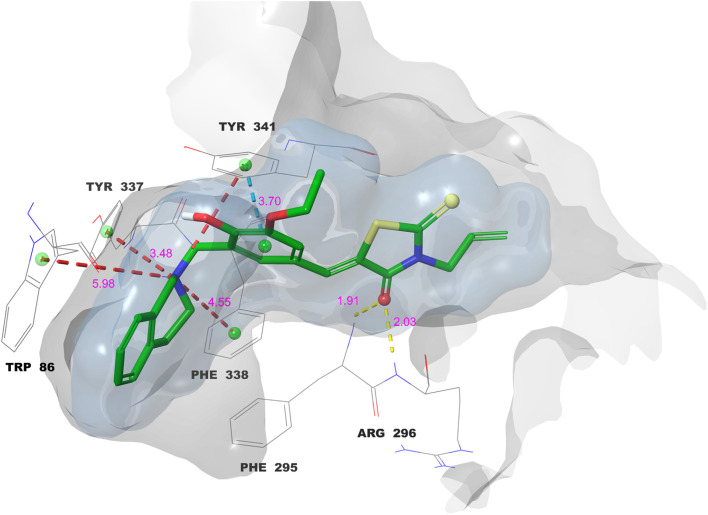
Molecular docking 3D ligand–protein interactions of 11–AChE complex.

The interactions of the 11–AChE complex are depicted in Fig. 6 in 3D form with detailed visualization of the main binding interactions. The yellow dashed lines indicate the hydrogen bonds whose lengths are in the range of 1.91–2.03 Å, thus confirming their strong and stable nature. The turquoise dashed line shows the π–π stacking interaction between the benzylidene ring and Tyr-341 with the length of 3.70 Å, which is a good stack. The four cation–π interactions are shown in red dashed lines between the aminomethyl nitrogen and Trp-86, Tyr-337, Tyr-341, and Phe-338, with bond distances ranging from 3.48 to 5.98 Å. The importance of such interactions is amplified because Trp-86 is situated in the catalytic active site of AChE, and it is a well-established fact that cation–π interactions enhance greatly the binding affinity of ligands as well as the target selectivity. The figure illustrates the binding surfaces: the bluish cloud represents the ligand's surface binding regions, whereas the grayish cloud denotes the protein's surface binding regions. The two surfaces fully coincide, indicating that the ligand is optimally positioned within the active site.

**Fig. 7:**
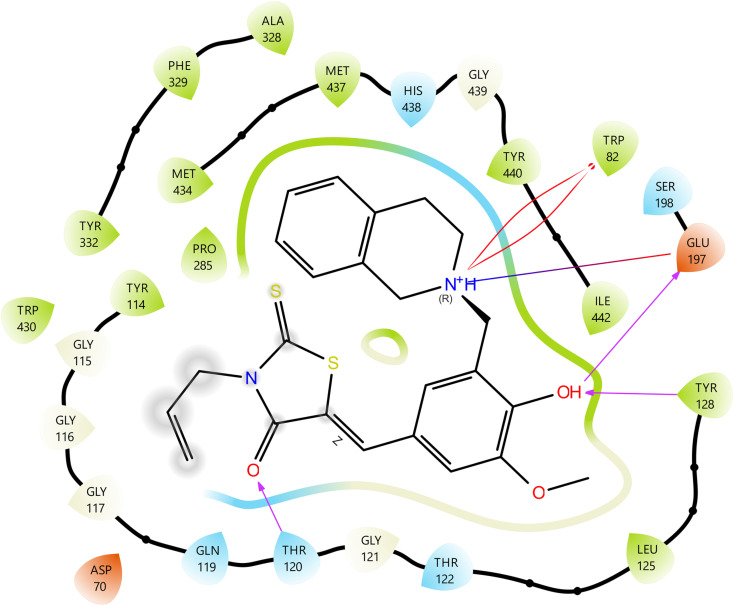
Molecular docking 2D ligand–protein interactions of 6–BChE complex.

In the case of 6–BChE, the carbonyl oxygen of the rhodanine made a hydrogen bond with Thr-120 that served to stabilize the interaction, while the phenolic hydroxyl group provided further hydrogen bonds with Tyr-128 and Glu-197, thus gripping the ligand in the active site (Fig. 7). Tetrahydroisoquinoline nitrogen formed a salt bridge with Glu-197 while simultaneously participating in two cation–π interactions with Trp-82. The importance of Trp-82 in recognizing the substrate within the anionic catalytic site of BChE is substantiated, and the interaction of cation–π along with a salt bridge serves to demonstrate the occurrence of strong electrostatic as well as aromatic stabilization.^44^ Furthermore, the hydrogen bonds, in conjunction with robust interactions, account for the compound's elevated binding affinity and suggest significant competitive inhibition of BChE.

**Fig. 8:**
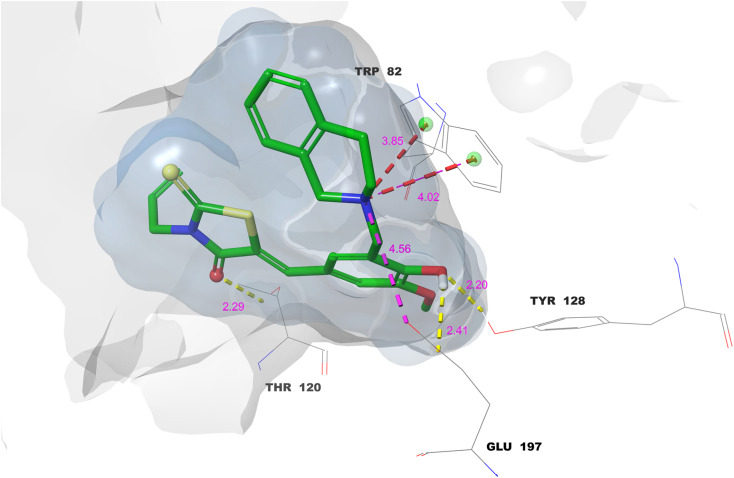
Molecular docking 3D ligand–protein interactions of 6–BChE complex.

The active site of BChE used the 3D docking model of compound 6 to show that the compound was binding in a very organized way, which was also backed by a mesh of interactions that were spatially very favorable (Fig. 8). The rhodanine carbonyl oxygen formed a strong hydrogen bond with Thr-120 (2.29 Å), which likely contributes to anchoring the ligand near the mid-gorge region of the active site. Furthermore, the phenolic OH was involved in hydrogen bonds with Tyr-128 and Glu-197, which are the main residues at the mouth of the gorge, with lengths of 2.20 and 2.41 Å respectively, accentuating the ligand's positioning and overall orientation. The aminomethyl nitrogen got involved in two cation–π interactions with Trp-82 at 3.85 Å and 4.02 Å, thus imitating the interaction mode of the endogenous substrates such as acetylcholine. This same nitrogen also formed a salt bridge with Glu-197 (4.56 Å), which resulted in increased electrostatic stabilization and possibly the prolonging of the ligand's residence time in the active site gorge.

The molecular docking and interaction analyses that were done in detail indicate that the exceptional binding profiles of compounds 11 and 6 towards AChE and BChE, respectively, are favorable. Compound 11 is the most powerful AChE inhibitor, interacts with enzymes through the formation of key hydrogen bonds, huge π–π stacking, and several cation–π contacts with amino acids that are important for substrate recognition and catalysis. In a similar way, compound 6 shows a good coordination of hydrogen bonds, cation–π interactions, and salt bridge formation at the BChE active site, which in unison result in its high binding affinity and predicted inhibitory efficacy. The multi-modal binding interactions have the dual effect of increasing the stability of the ligand–enzyme complexes and providing a mechanism of competitive inhibition in agreement with their *in vitro* enzyme inhibition profiles.

### 2.5. Molecular dynamics simulations

**Fig. 9:**
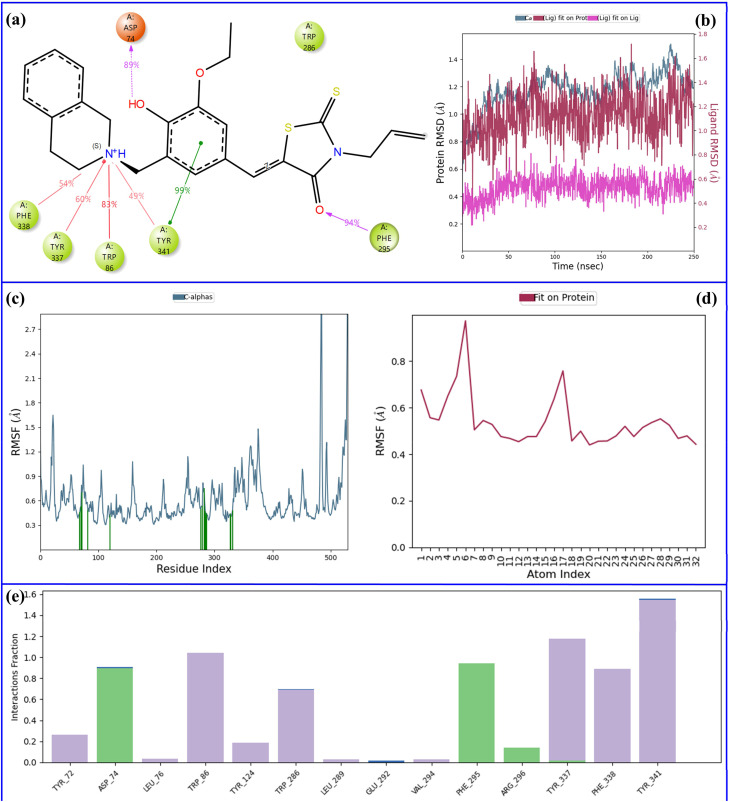
Analysis of the 250 ns MD simulation of 11–AChE complex. (a) 2D representation of important interactions between ligand and protein, (b) comparative plot of RMSD of ligand and protein atoms, (c) RMSF of protein atoms, (d) RMSF of ligand atoms, (e) histogram of fractional interactions.

**Fig. 10:**
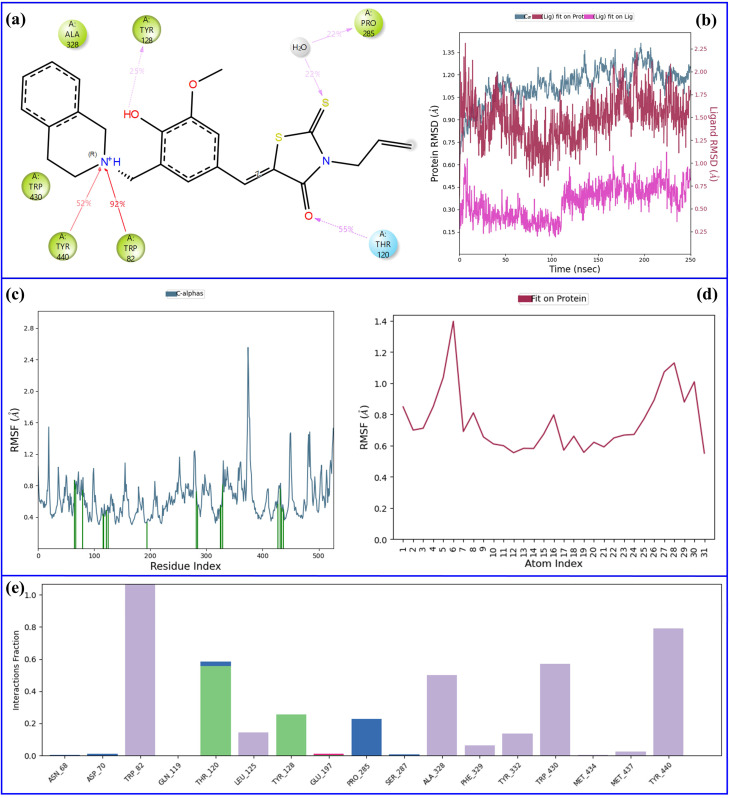
Analysis of the 250 ns MD simulation of 6–BChE complex. (a) 2D representation of important interactions between ligand and protein, (b) comparative plot of RMSD of ligand and protein atoms, (c) RMSF of protein atoms, (d) RMSF of ligand atoms, (e) histogram of fractional interactions.

Molecular dynamics (MD) simulations are one of the most powerful techniques preferred in the discovery of new drugs, providing very clear information about the stability of protein–ligand complexes. Two critical measures, Root Mean Square Deviation (RMSD) and Root Mean Square Fluctuation (RMSF), are usually employed to assess overall conformational stability and residue-specific flexibility, respectively. While RMSD indicates the total structural movements of the complex in the course of the simulation, RMSF points out the areas of the protein that are moving and that could have a role in the binding of the ligand and the recognition of the molecules.^45^ Based on the results from *in vitro* enzyme inhibition testing and molecular docking investigations, molecular dynamics simulations were conducted on the most promising inhibitors: compound 11 for acetylcholinesterase and compound 6 for butyrylcholinesterase. Fig. 9 and 10 are used to show the dynamic behavior of these complexes throughout the 250 ns simulations indicating their stability as well as their interaction patterns.

The main interactions between compound 11 and AChE are depicted in Fig. 9a. During the 250 ns MD simulation, the ligand establishes a network of highly persistent interactions with key binding-site residues. The rhodanine carbonyl forms a strong hydrogen bond (purple arrows) with Phe-295 (94% occupancy), and the phenolic OH engages Asp-74 *via* a hydrogen bond (89% occupancy), both indicating long lived polar anchoring of the scaffold. The benzylidene ring is nearly constantly involved in π–π stacking (green line) with Tyr-341 (99% occupancy) which greatly stabilizes the ligand through aromatic interactions in the pocket. On top of this, the tetrahydroisoquinoline nitrogen does create some remarkable cation–π interactions (red lines) with Trp-86 (83% occupancy), Tyr-337 (60%), Phe-338 (54%) and once in a while with Tyr-341 (49%), which all together have led to the assumption that electrostatic/aromatic interactions are of great significance in ligand retention and orientation.

The protein's Cα atoms (pale blue) showed an average RMSD value of 1.2 Å throughout the entire simulation, which meant that the backbone conformation was very stable with almost no structural deviation from the initial equilibrated structure. The ligand also showed an average RMSD of 1.2 Å (red), therefore closely following the stability of the binding site. Furthermore, the low RMSD of the ligand (pink) (0.5 Å) indicates that its binding pose was essentially unchanged during the entire simulation, only with minor thermal fluctuations (Fig. 9b). These values together denote a protein–ligand complex that was well-stabilized where both the receptor and the ligand showed high conformational stability, thus, supporting the robustness of the docked pose under dynamic conditions.

Fig. 9c and d present the results of the entire RMSF analysis for the protein's Cα atoms and the ligand atoms, respectively, throughout the simulation. The RMSF values averaged across the board for both the Cα atoms of the protein and the ligand were about 0.6 Å, which means that there was a very high local structural rigidity and slight atomic movements during the entire simulation. The protein backbone exhibited such low RMSF values that it was interpreted that the protein was in a stable and well-folded state with limited mobility which is indicative of a well-folded and equilibrated system. The low RMSF value of the ligand supports this interpretation as it points to a very close and strong interaction with the binding pocket and only slight shifts from the average position of the ligand. Fig. 9c also shows the contacts between the ligand and the protein very clearly, with green vertical lines indicating approximately twenty amino acid–ligand contacts.

Fig. 9e presents an interaction histogram illustrating the variety and frequency of contacts formed between the ligand and protein residues. Hydrophobic interactions are shown in the grey color, hydrogen bonds in the green color, and water-mediated hydrogen bonds in the blue color. The histogram sorts interactions by the ligand's functional groups, acknowledging that every group can connect with various residues, while single residues may at the same time engage in interactions with several ligand groups. This detailed representation efficiently summarizes the major interaction patterns. The amino acids Asp-74, Trp-86, Phe-295, Tyr-337, Phe-338, and Tyr-341 are particularly noticed as the ones with the highest interaction frequencies, indicating their indispensable function in the ligand's anchoring at the active site.

The 250 ns MD analysis of 6–BChE complex is given in Fig. 10. During the simulation, the ligand established several significant interactions with the BChE active site residues. The carbonyl oxygen of rhodanine established a hydrogen bond of moderate strength with Thr-120 (occupancy 55%), while the sulfur atom of the thiocarbonyl group in rhodanine took part in a water-mediated hydrogen bond with Pro-285 (occupancy 22%). Furthermore, the phenolic –OH group was the hydrogen bond donor to Tyr-128 (occupancy 25%). On top of that, the nitrogen atom of tetrahydroisoquinoline was the strong cation–π interactions partner with Trp-82 (occupancy 92%) and Tyr-440 (occupancy 52%) (Fig. 10a). These continuous polar and aromatic contacts were among the main stabilizing forces keeping the ligand in the BChE binding pocket all through the MD run.

Fig. 10b illustrates the RMSD analysis of the ligand and protein atoms over the full simulation duration. The protein's Cα atoms showed an average RMSD of 1.1 Å during the simulation, which is a clear indication of a stable backbone conformation with negligible structural fluctuations. The ligand, on the other hand, showed a higher average RMSD of 1.75 Å, which could be interpreted as the binding pocket being more flexible or mobile than the protein. Nevertheless, the ligand's deviation from the initial docked position was only 0.7 Å, which is still quite small and suggests that the ligand had been adjusting conformationally but had remained in its original binding pose for the most part.

The mean RMSF values for the Cα atoms of the protein and the ligand were ∼0.8 Å, denoting the presence of moderate flexibility in the system (Fig. 10c and d). The protein backbone kept a rather stable conformation along with some localized variations, which is a regular behavior of proteins in physiological conditions. In the same way, the ligand showed similar atomic fluctuations which, on the one hand, indicated that it was still in a “locked” conformation but, on the other hand, it permitted for the dynamic adjustments that were absolutely necessary in the binding pocket.

In the interaction histogram shown in Fig. 10e, the various interaction types between the ligand and the protein are presented. The histogram reveals that the highest number of interactions were with the residues Trp-82, Thr-120, Trp-430, and Tyr-440, thus, their role in holding the ligand in the active site and stabilizing it was prominently pointed out.

Thus, the extensive 250 ns MD simulations serve as a proof for the stability and presence of very strong interaction between the compounds 11 and 6 in AChE and BChE, active sites respectively. Compound 11 shows a very stable and also an extremely well supported conformation through a very strong and abundant network comprising of persistent hydrogen bonds, π–π stacking and cation–π interactions which together sort of hold the ligand and keep it firmly in the AChE binding pocket. The low values of RMSD and RMSF give additional confirmation of very small structural changes and atomic movements, which are characteristic of a rigid and properly managed protein–ligand complex. Similarly, 6 exhibits significant polar and aromatic interactions with key BChE residues, although its ligand RMSD suggests somewhat higher flexibility within the binding site. Nonetheless, the ligand preserves its overall binding pose, supported by moderate protein and ligand RMSF values, reflecting a dynamic but specific interaction. These results, along with the first two compounds, confirm the strong inhibition potential of both and open up their molecular-level binding mechanisms for discussion, thereby drawing attention to their likelihood as lead candidates in cholinesterase inhibition.

## 3. Conclusion

In this study, we created a new set of 3,5-disubstituted rhodanine derivatives with tertiary amine groups, using a molecular hybridization approach to create dual AChE/BChE inhibitors. The new compounds displayed not only strong inhibitory activity but also selectivity, with some being dual inhibitors while others preferred AChE. Cytotoxicity tests on HUVEC cells confirmed the safety of these substances at the concentrations which were effective for enzyme inhibition. The experimental findings were backed by computational analyses, like molecular docking, MM-GBSA and molecular dynamics simulations, which also revealed the important interactions with the enzymes. The most significant conceptual progress of this research is the combination of the rhodanine scaffold with tertiary amine groups thereby resulting in the formation of multifunctional molecules that can penetrate both cholinesterase isoforms. The dual-target strategy may provide therapeutic benefits in Alzheimer's disease by treating cholinergic deficiencies at various stages.

However, it is important to acknowledge the limitations of this study. Our investigations were primarily based on *in vitro* enzyme inhibition and cytotoxicity assays and *in silico* computational analyses, including molecular docking and MD simulations. While these approaches provide valuable mechanistic insights and strong preliminary evidence of inhibitory potential, they do not fully capture the complexity of biological systems *in vivo*. Therefore, further studies involving cellular assays, pharmacokinetic profiling, toxicity evaluations in animal models, and ultimately clinical investigations are necessary to comprehensively validate the therapeutic efficacy and safety of these rhodanine-based compounds. Addressing these aspects will be crucial to advancing these promising molecules from bench to bedside in the context of Alzheimer's disease treatment.

## 4. Material and methods

### 4.1. Chemistry

The different vendors provided the chemicals that were used in this research. The melting points of the substances were measured using the WRS-2A Microprocessor Melting-point Apparatus and the readings were uncorrected. The compounds' FTIR spectra were taken with the Alpha-P Bruker FT-IR spectrophotometer. The ^1^H NMR spectra were recorded using a Bruker (400 MHz) spectrometer. A Bruker (100 MHz) spectrometer was used for the ^13^C NMR-APT spectra. The chemical shifts are given as *δ* in ppm with respect to tetramethylsilane (TMS) (*δ* 0.00 singlet) in deuterated chloroform (CDCl_3_). HRMS data were obtained through electrospray ionization (ESI) on a Thermo Fisher Scientific Q Exactive™ Hybrid Quadrupole-Orbitrap™ instrument.

#### 4.1.1. Synthesis of 3-allylrhodanine (3AlRh)

Allylamine (10 mmol) and triethylamine (15 mmol) were dissolved in ethanol (10 mL) and the solution was stirred on ice bath. CS_2_ (20 mmol) was added to this solution by dropwise and stirring was continued until precipitates formed. The formed solid was filtered off and washed with cold diethyl ether. A solution of chloroacetic acid (20 mmol) and sodium chloroacetate (10 mmol) in distilled water (15 mL) was prepared, and the solid obtained from the first step was added to this solution. The mixture was then stirred for about two hours at room temperature until it gave an orange color. Then, 6 N HCl (100 mL) was added, and stirring was continued for an additional 10 minutes. The formed crude product was filtered off and recrystallized from ethanol-diethyl ether (1 : 1) mixture (Fig. 2).

#### 4.1.2. Synthesis of Mannich-type aldehydes (A1–13)

Formaldehyde (15 mmol, w/w: 37%) and corresponding secondary amine (12 mmol) were dissolved in absolute ethanol (30 mL) and the mixture was refluxed for one hour. To this mixture the phenolic aldehyde (vanillin, ethylvanillin, 3-bromo-4-hydroxybenzaldehyde or 4-hydroxybenzaldehyde) (10 mmol) was added and the final solution was refluxed for 4–8 hours. After completion, the mixture was cooled to room temperature and left in the freezer overnight. The formed crude product was filtered off and recrystallized from ethanol (Fig. 2).

#### 4.1.3. Synthesis of compounds 1–13

3AlRh (10 mmol) and A1–13 (10 mmol) were dissolved in absolute ethanol (20 mL) and catalytic amount of piperidine (3–4 drops) was added to this solution. The mixture was refluxed for 4–5 hours and cooled to room temperature. The formed crude product was filtered off and recrystallized from ethanol (Fig. 2).

##### 4.1.3.1 (*Z*)-3-Allyl-5-[4-hydroxy-3-methoxy-5-(morpholinomethyl)benzylidene]-2-thioxothiazolidin-4-one (1)

Orange solid, yield: 78%, mp: 190–192 °C. FTIR (cm^−1^): *ν*_max_ 2935, 1706, 1575, 1263, 1118, 1026. ^1^H NMR (400 MHz, CDCl_3_) *δ* 7.63 (s, 1H), 6.94 (s, 1H), 6.84 (s, 1H), 5.92–5.82 (m, 1H), 5.31–5.24 (m, 2H), 4.75 (d, *J* = 5.6 Hz, 2H), 3.95 (s, 3H), 3.80 (s, 2H), 3.78 (s, 4H), 2.63 (s, 4H). ^13^C NMR (100 MHz, CDCl_3_) *δ* 192.8, 167.5, 150.6, 148.7, 133.8, 129.6, 125.1, 124.7, 121.4, 119.2, 112.7, 66.7, 61.4, 56.1, 52.8, 46.4. HRMS-ESI (*m*/*z*) calculated for C_19_H_23_N_2_O_4_S_2_ [M + H]^+^: 407.1099, found: 407.1086.

##### 4.1.3.2 (*Z*)-3-Allyl-5-{4-hydroxy-3-methoxy-5-[(4-methylpiperazin-1-yl)methyl]benzylidene}-2-thioxothiazolidin-4-one (2)

Yellow solid, yield: 76%, mp: 137–139 °C. FTIR (cm^−1^): *ν*_max_ 2935, 1706, 1579, 1254, 1133, 1037. ^1^H NMR (400 MHz, CDCl_3_) *δ* 7.63 (s, 1H), 6.93 (s, 1H), 6.83 (s, 1H), 5.92–5.84 (m, 1H), 5.31–5.24 (m, 2H), 4.75 (d, *J* = 5.5 Hz, 2H), 3.94 (s, 3H), 3.80 (s, 2H), 2.64 (s, 8H), 2.32 (s, 3H). ^13^C NMR (100 MHz, CDCl_3_) *δ* 192.8, 167.5, 151.0, 148.7, 134.0, 129.6, 125.1, 124.4, 121.7, 119.2, 112.6, 60.9, 56.1, 54.7, 52.4, 46.4, 45.8. HRMS-ESI (*m*/*z*) calculated for C_20_H_26_N_3_O_3_S_2_ [M + H]^+^: 420.1416, found: 420.1403.

##### 4.1.3.3 (*Z*)-3-Allyl-5-{4-hydroxy-3-methoxy-5-[(4-phenylpiperazin-1-yl)methyl]benzylidene}-2-thioxothiazolidin-4-one (3)

Yellow solid, yield: 85%, mp: 207–209 °C. FTIR (cm^−1^): *ν*_max_ 2958, 1690, 1572, 1240, 1140, 1046. ^1^H NMR (400 MHz, CDCl_3_) *δ* 7.65 (s, 1H), 7.30–7.26 (m, 3H), 6.94–6.86 (m, 5H), 5.92–5.83 (m, 1H), 5.32–5.24 (m, 2H), 4.75 (d, *J* = 5.7 Hz, 2H), 3.95 (s, 3H), 3.86 (s, 2H), 3.26 (s, 4H), 2.79 (s, 4H). ^13^C NMR (100 MHz, CDCl_3_) *δ* 192.8, 167.5, 150.8, 148.7, 133.9, 129.7, 129.3, 125.1, 124.6, 121.7, 120.5, 119.2, 116.5, 112.7, 61.0, 56.1, 52.5, 49.2, 46.4. HRMS-ESI (*m*/*z*) calculated for C_25_H_28_N_3_O_3_S_2_ [M + H]^+^: 482.1572, found: 482.1559.

##### 4.1.3.4 (*Z*)-3-Allyl-5-{4-hydroxy-3-methoxy-5-[(4-ethoxycarbonylpiperazin-1-yl)methyl]benzylidene}-2-thioxothiazolidin-4-one (4)

Yellow solid, yield: 79%, mp: 151–153 °C. FTIR (cm^−1^): *ν*_max_ 2960, 1695, 1578, 1242, 1122, 1033. ^1^H NMR (400 MHz, CDCl_3_) *δ* 7.63 (s, 1H), 6.94 (s, 1H), 6.83 (s, 1H), 5.91–5.84 (m, 1H), 5.31–5.24 (m, 2H), 4.75 (d, *J* = 5.0 Hz, 2H), 4.16 (q, *J* = 6.8 Hz, 2H), 3.95 (s, 3H), 3.80 (s, 2H), 3.56 (s, 4H), 2.58 (s, 4H), 1.27 (t, *J* = 6.8 Hz, 3H). ^13^C NMR (100 MHz, CDCl_3_) *δ* 192.7, 167.5, 155.2, 150.4, 148.7, 133.7, 129.6, 125.0, 124.8, 121.5, 119.4, 119.2, 112.7, 61.7, 61.1, 56.1, 52.3, 46.4, 43.4, 14.6. HRMS-ESI (*m*/*z*) calculated for C_22_H_28_N_3_O_3_S_2_ [M + H]^+^: 478.1470, found: 478.1457.

##### 4.1.3.5 (*Z*)-3-Allyl-5-{4-hydroxy-3-methoxy-5-[(3-methylpiperidin-1-yl)methyl]benzylidene}-2-thioxothiazolidin-4-one (5)

Orange solid, yield: 73%, mp: 120–122 °C. FTIR (cm^−1^): *ν*_max_ 2932, 1707, 1576, 1254, 1133, 1094. ^1^H NMR (400 MHz, CDCl_3_) *δ* 7.64 (s, 1H), 6.92 (s, 1H), 6.81 (s, 1H), 5.92–5.84 (m, 1H), 5.31–5.24 (m, 2H), 4.74 (d, *J* = 5.3 Hz, 2H), 3.94 (s, 3H), 2.95–2.90 (m, 2H), 2.13–2.08 (m, 1H), 1.82–1.61 (m, 5H), 0.98–0.89 (m, 4H). ^13^C NMR (100 MHz, CDCl_3_) *δ* 192.9, 167.6, 151.9, 148.7, 134.3, 129.7, 125.0, 124.0, 122.0, 119.1, 118.5, 112.5, 61.5, 60.8, 56.0, 53.3, 46.4, 32.3, 31.1, 25.1, 19.3. HRMS-ESI (*m*/*z*) calculated for C_21_H_27_N_2_O_3_S_2_ [M + H]^+^: 419.1463, found: 419.1450.

##### 4.1.3.6 (*Z*)-3-Allyl-5-{3-[(3,4-dihydroisoquinolin-2(1*H*)-yl)methyl]-4-hydroxy-5-methoxybenzylidene}-2-thioxothiazolidin-4-one (6)

Orange solid, yield: 81%, mp: 157–159 °C. FTIR (cm^−1^): *ν*_max_ 2963, 1706, 1578, 1251, 1133, 1037. ^1^H NMR (400 MHz, CDCl_3_) *δ* 7.66 (s, 1H), 7.16–7.12 (m, 3H), 7.01 (d, *J* = 6.5 Hz, 1H), 6.95 (s, 1H), 6.88 (s, 1H), 5.91–5.84 (m, 1H), 5.32–5.24 (m, 2H), 4.75 (d, *J* = 5.3 Hz, 2H), 3.97 (s, 2H), 3.93 (s, 3H), 3.82 (s, 2H), 2.96 (dd, *J* = 19.3, 4.6 Hz, 4H). ^13^C NMR (100 MHz, CDCl_3_) *δ* 192.8, 167.6, 151.2, 148.8, 134.0, 133.1, 132.6, 129.7, 128.7, 126.9, 126.6, 126.2, 125.1, 124.5, 121.9, 119.2, 112.8, 60.6, 56.1, 55.2, 50.0, 46.4, 28.4. HRMS-ESI (*m*/*z*) calculated for C_24_H_25_N_2_O_3_S_2_ [M + H]^+^: 453.1307, found: 453.1291.

##### 4.1.3.7 (*Z*)-3-Allyl-5-(4-hydroxy-3-methoxy-5-{[4-(pyrimidin-2-yl)piperazin-1-yl]methyl}benzylidene)-2-thioxothiazolidin-4-one (7)

Yellow solid, yield: 85%, mp: 217–219 °C. FTIR (cm^−1^): *ν*_max_ 2834, 1694, 1577, 1252, 1136, 1050. ^1^H NMR (400 MHz, CDCl_3_) *δ* 8.32 (d, *J* = 4.7 Hz, 2H), 7.65 (s, 1H), 6.96 (s, 1H), 6.84 (s, 1H), 6.53 (t, *J* = 4.7 Hz, 1H), 5.91–5.84 (m, 1H), 5.31–5.24 (m, 2H), 4.75 (d, *J* = 5.6 Hz, 2H), 3.96 (s, 3H), 3.92 (s, 4H), 3.83 (s, 2H), 2.68 (s, 4H). ^13^C NMR (100 MHz, CDCl_3_) *δ* 192.8, 167.5, 157.8, 150.8, 148.8, 133.8, 129.7, 125.0, 124.7, 121.7, 119.2, 112.8, 110.4, 61.2, 56.1, 52.5, 46.4, 43.4. HRMS-ESI (*m*/*z*) calculated for C_23_H_26_N_5_O_3_S_2_ [M + H]^+^: 484.1477, found: 484.1462.

##### 4.1.3.8 (*Z*)-3-Allyl-5-[4-hydroxy-3-ethoxy-5-(morpholinomethyl)benzylidene]-2-thioxothiazolidin-4-one (8)

Orange solid, yield: 75%, mp: 145–147 °C. FTIR (cm^−1^): *ν*_max_ 2955, 1704, 1571, 1266, 1113, 1075. ^1^H NMR (400 MHz, CDCl_3_) *δ* 7.62 (s, 1H), 6.93 (s, 1H), 6.82 (s, 1H), 5.92–5.84 (m, 1H), 5.31–5.24 (m, 2H), 4.74 (d, *J* = 5.5 Hz, 2H), 4.14 (q, *J* = 6.9 Hz, 2H), 3.78 (s, 6H), 2.62 (s, 4H), 1.53 (t, *J* = 6.9 Hz, 3H). ^13^C NMR (100 MHz, CDCl_3_) *δ* 192.8, 167.5, 150.7, 148.0, 133.9, 129.6, 125.1, 124.6, 121.5, 119.2, 119.1, 113.8, 66.6, 64.5, 61.4, 52.8, 46.4, 14.8. HRMS-ESI (*m*/*z*) calculated for C_20_H_25_N_2_O_4_S_2_ [M + H]^+^: 421.1256, found: 421.1242.

##### 4.1.3.9 (*Z*)-3-Allyl-5-{4-hydroxy-3-ethoxy-5-[(4-ethoxycarbonylpiperazin-1-yl)methyl]benzylidene}-2-thioxothiazolidin-4-one (9)

Yellow solid, yield: 73%, mp: 158–160 °C. FTIR (cm^−1^): *ν*_max_ 2982, 1696, 1577, 1241, 1131, 1072. ^1^H NMR (400 MHz, CDCl_3_) *δ* 7.63 (s, 1H), 6.94 (s, 1H), 6.82 (s, 1H), 5.92–5.84 (m, 1H), 5.31–5.24 (m, 2H), 4.74 (d, *J* = 5.3 Hz, 2H), 4.16–4.12 (m, 4H), 3.79 (s, 2H), 3.56 (s, 4H), 2.58 (s, 4H), 1.53 (t, *J* = 6.9 Hz, 3H), 1.27 (t, *J* = 7.0 Hz, 3H). ^13^C NMR (100 MHz, CDCl_3_) *δ* 192.8, 167.5, 167.4, 155.2, 150.6, 148.0, 133.9, 129.6, 125.0, 124.7, 121.6, 119.2, 113.8, 64.6, 61.7, 61.1, 52.3, 46.4, 43.3, 14.7. HRMS-ESI (*m*/*z*) calculated for C_23_H_30_N_3_O_5_S_2_ [M + H]^+^: 492.1627, found: 492.1614.

##### 4.1.3.10 (*Z*)-3-Allyl-5-{4-hydroxy-3-ethoxy-5-[(4-phenylpiperazin-1-yl)methyl]benzylidene}-2-thioxothiazolidin-4-one (10)

Yellow solid, yield: 82%, mp: 171–173 °C. FTIR (cm^−1^): *ν*_max_ 2988, 1693, 1574, 1240, 1138, 1048. ^1^H NMR (400 MHz, CDCl_3_) *δ* 7.64 (s, 1H), 7.30–7.23 (m, 3H), 6.95–6.85 (m, 4H), 5.92–5.84 (m, 1H), 5.32–5.24 (m, 2H), 4.75 (d, *J* = 5.1 Hz, 2H), 4.15 (q, *J* = 6.8 Hz, 2H), 3.84 (s, 2H), 3.26 (s, 4H), 2.79 (s, 4H), 1.53 (t, *J* = 6.9 Hz, 3H). ^13^C NMR (100 MHz, CDCl_3_) *δ* 192.9, 167.6, 151.0, 150.8, 148.0, 134.0, 129.7, 129.3, 125.1, 124.5, 121.8, 120.5, 119.2, 119.0, 116.5, 113.8, 64.6, 61.1, 52.6, 49.2, 46.4, 14.8. HRMS-ESI (*m*/*z*) calculated for C_26_H_30_N_3_O_3_S_2_ [M + H]^+^: 496.1729, found: 496.1714.

##### 4.1.3.11 (*Z*)-3-Allyl-5-{3-[(3,4-dihydroisoquinolin-2(1*H*)-yl)methyl]-4-hydroxy-5-ethoxybenzylidene}-2-thioxothiazolidin-4-one (11)

Orange solid, yield: 80%, mp: 158–160 °C. FTIR (cm^−1^): *ν*_max_ 2978, 1702, 1576, 1273, 1133, 1011. ^1^H NMR (400 MHz, CDCl_3_) *δ* 7.65 (s, 1H), 7.17–7.11 (m, 3H), 7.00 (d, *J* = 6.1 Hz, 1H), 6.95 (s, 1H), 6.87 (s, 1H), 5.93–5.84 (m, 1H), 5.32–5.24 (m, 2H), 4.75 (d, *J* = 5.6 Hz, 2H), 4.13 (q, *J* = 6.8 Hz, 2H), 3.96 (s, 2H), 3.80 (s, 2H), 2.97 (dd, *J* = 16.2, 5.1 Hz, 4H), 1.50 (t, *J* = 6.9 Hz, 3H). ^13^C NMR (100 MHz, CDCl_3_) *δ* 192.9, 167.6, 151.5, 148.1, 134.1, 133.1, 132.6, 129.7, 128.7, 126.8, 126.6, 126.1, 125.1, 124.4, 122.0, 119.2, 114.0, 64.6, 60.7, 55.1, 50.2, 46.4, 28.4, 14.8. HRMS-ESI (*m*/*z*) calculated for C_25_H_27_N_2_O_3_S_2_ [M + H]^+^: 467.1463, found: 467.1451.

##### 4.1.3.12 (*Z*)-3-Allyl-5-[3-bromo-4-hydroxy-5-(morpholinomethyl)benzylidene]-2-thioxothiazolidin-4-one (12)

Orange solid, yield: 78%, mp: 227–229 °C. FTIR (cm^−1^): *ν*_max_ 2930, 1705, 1581, 1258, 1118, 1022. ^1^H NMR (400 MHz, CDCl_3_) *δ* 7.62 (s, 1H), 7.56 (s, 1H), 7.10 (s, 1H), 5.91–5.83 (m, 1H), 5.31–5.24 (m, 2H), 4.74 (d, *J* = 5.3 Hz, 2H), 3.80 (s, 6H), 2.65 (s, 4H). ^13^C NMR (100 MHz, CDCl_3_) *δ* 192.4, 167.4, 157.4, 135.2, 131.7, 130.1, 129.5, 125.8, 122.4, 120.7, 119.4, 111.6, 66.5, 61.6, 52.7, 46.4. HRMS-ESI (*m*/*z*) calculated for C_18_H_20_BrN_2_O_3_S_2_ [M + H]^+^: 455.0099, found: 457.0063 (^81^Br).

##### 4.1.3.13 (*Z*)-3-Allyl-5-[4-hydroxy-3,5-bis(morpholinomethyl)benzylidene]-2-thioxothiazolidin-4-one (13)

Yellow solid, yield: 75%, mp: 165–167 °C. FTIR (cm^−1^): *ν*_max_ 2949, 1701, 1578, 1299, 1157, 1046. ^1^H NMR (400 MHz, CDCl_3_) *δ* 7.66 (s, 1H), 7.29 (s, 2H), 5.93–5.84 (m, 1H), 5.31–5.24 (m, 2H), 4.75 (d, *J* = 5.2 Hz, 2H), 3.77 (s, 8H), 3.68 (s, 4H), 2.57 (s, 8H). ^13^C NMR (100 MHz, CDCl_3_) *δ* 193.0, 167.6, 159.3, 133.8, 131.9, 129.7, 124.4, 123.7, 119.4, 119.2, 66.8, 59.0, 53.3, 46.4. HRMS-ESI (*m*/*z*) calculated for C_23_H_30_N_3_O_4_S_2_ [M + H]^+^: 476.1678, found: 476.1667.

### 4.2. AChE and BChE inhibition assay

AChE derived from *Electrophorus electricus* (electric eel; specific activity: 200–1000 U per mg protein) and BChE isolated from equine serum (≥10 U per mg protein) were obtained from Sigma-Aldrich (St. Louis, MO, USA). Acetylthiocholine chloride (ATChCl) and butyrylthiocholine iodide (BTChI) were utilized as the respective substrates for AChE and BChE assays. All reagent solutions were freshly prepared in 0.1 M Tris–HCl buffer at pH 8.0. The enzymatic activities were assessed spectrophotometrically following a modified protocol based on Ellman's colorimetric method.^46^ The reactions were initiated by the addition of substrate, and the formation of the colored product was monitored at 412 nm using a UV-vis spectrophotometer. To determine the inhibition constant (*K*_i_), a range of inhibitor concentrations was tested under steady-state conditions. Enzyme kinetic parameters were derived by measuring initial velocities at varying substrate concentrations both in the presence and absence of inhibitors. The *K*_i_ values were obtained through Lineweaver–Burk double-reciprocal plots, where 1/*V* was plotted against 1/[S], allowing for graphical estimation based on the shift in intercepts.^47,48^

#### 4.2.1. Pearson correlation and heatmap analysis

Pearson correlation and linear regression analysis between the *K*_i_ values of AChE and BChE was conducted using R software (version 4.5, R Core Team), and the plot was generated with the ggplot2 package. A confidence interval of 95% was shaded in the plot to indicate the regression line's reliability. The *K*_i_ values were as such compared using molecular modeling of acetylcholinesterase and butyrylcholinesterase, made comfortable with ggplot2 configuration in R version 4.5 by R Core Team. A gradient scale representing increasing *K*_i_ values was employed to depict the color intensities where dark blue (complete inhibition) and light yellow (minimal inhibition) were the ends of the scale. The heat map gave an easy-to-see summary of the inhibition trends for both enzymes and made it quick to point out the dual potent inhibitors in comparison with the reference compound tacrine.^49,50^

### 4.3. Cytotoxicity

#### 4.3.1. Cell cultures

HUVEC cells (ATCC CRL-1730) were used as a control to evaluate the non-toxic effects of the compounds on healthy tissue. The cell line was cultured in DMEM-F12 medium (Gibco, MD, USA) supplemented with 10% fetal bovine serum (FBS) (Gibco, MD, USA) and 1% penicillin/streptomycin (Gibco, MD, USA) under a 5% CO_2_ humidified atmosphere at 37 °C.

#### 4.3.2. MTT cell viability assay

The MTT proliferation test was conducted to determine the impact of the compounds on cell viability in the HUVEC cell line. The cells were seeded in 96-well plates (1 × 104 cells per well) and subsequently allowed to grow for 24 hours. After the cells were treated for 24 hours with the seven different concentrations of the compounds (3.125, 6.25, 12.5, 25, 50, 100, and 200 µM), they were subjected to further treatment with DMSO as a solvent control, ensuring that its final concentration did not exceed 1%. Following the treatment duration, MTT solution was added at a final concentration of 0.1 mg mL^−1^, and the cells were incubated for 4 hours at 37 °C. Then, the supernatant was discarded, DMSO was added to allow the formazan crystals to dissolve, and the plates were incubated in the dark at room temperature for 30 minutes. The absorbance at 570 nm was determined using a microplate reader (BioTek Instruments, Inc., USA). All cytotoxicity assays were done in three times (three independent experiments). IC_50_ values are given as mean ± standard deviation (SD). The SD values that are comparatively low indicate that the assay was carried out under highly precise and reproducible conditions. The error bars in all the figures show the experimental SD. For instance, compound 4 showed an IC_50_ of 73.13 ± 0.50 µM (mean ± SD, *n* = 3) and this is proof of the same activity all over the independent experiments.

### 4.4. Computational studies

Molecular docking study was carried out with Schrödinger Molecular Modeling Software (2025-1) using Maestro interface (v14.3) and Desmond. The preparation of protein and ligand was done in accordance with the protocols established by the group.^51^ The 3D structure of AChE (PDB ID: 4EY7) and BChE (PDB ID: 5NN0) were obtained from the Protein Data Bank and optimized with Schrödinger's Protein Preparation Wizard. Docking employed Glide XP and Induced Fit Docking (IFD) to consider receptor flexibility.^52,53^ For each ligand, 20 poses were generated, and top poses were selected based on IFD scores. Prime MM-GBSA calculations using the VSGB solvation model estimated binding free energies.^54^

All MD simulations were conducted using Desmond (Schrödinger Release 2024-3). The docking studies provided the protein–ligand complexes that were prepared by elimination of crystallographic water molecules that were farther than 10 Å from the ligand, proper protonation states at pH 7.0 were assigned, and hydrogen bonding networks were optimized. Each complex was placed into an orthorhombic simulation box with a 10 Å buffer added in all directions, TIP4P explicit water model was used for solvation, and the system was rendered neutral by adding counter ions (Na^+^ or Cl^−^) wherever necessary. For the purpose of imitating the physiological ionic strength, a further 0.15 M NaCl was added. The equilibrated system was heated slowly from 0 K to 300 K over a period of 200 ps under *NVT* ensemble using the Nosé–Hoover thermostat with a relaxation time of 1.0 ps. After that, there was pressure equilibration under *NPT* ensemble at 1.01325 bar employing the Martyna–Tobias–Klein barostat (relaxation time 2 ps) for 1 ns with positional restraints on protein backbone atoms. A production run of 250 ns was then performed at 300 K and 1.01325 bar in the *NPT* ensemble, with a 2 fs time step, long-range electrostatics treated using the particle mesh Ewald (PME) method and van der Waals interactions cutoff set at 9 Å. Trajectories were recorded every 100 ps. System stability and conformational changes were monitored using root mean square deviation (RMSD) and root mean square fluctuation (RMSF) analyses for both protein backbone and ligand heavy atoms. During system-wide trajectory monitoring, hydrogen bond interactions as well as ligand binding conformations were analyzed to determine the binding stability.^55^

## Conflicts of interest

The authors declare that they have no known competing financial interests or personal relationships that could have appeared to influence the work reported in this paper.

## Supplementary Material

RA-015-D5RA07416A-s001

## Data Availability

The data used for the manuscript entitled “New Mannich-type arylidenerhodanines as potent inhibitors of AChE and BChE: synthesis, biological evaluation, cytotoxicity and molecular modeling” will be included in an supplementary information (SI), available online on *RSC Advances* web site. Supplementary information: NMR (^1^H, ^13^C-APT), HRMS and FTIR spectra of new compounds. In addition, cell viability and cytotoxicity IC_50_ graphs, as well as Lineweaver–Burk graphs. See DOI: https://doi.org/10.1039/d5ra07416a.
