# Supplementary material for: New Mannich-type arylidenerhodanines as potent inhibitors of AChE and BChE: synthesis, biological evaluation, cytotoxicity and molecular modeling
Source: RSC Adv. 2025 Dec 15;15(58):50186–205. doi: 10.1039/d5ra07416a (PMC12704249; doi:10.1039/d5ra07416a)
Supplement: RA-015-D5RA07416A-s001 [file RA-015-D5RA07416A-s001.pdf]

Supplementary Information (SI) for RSC Advances.
This journal is © The Royal Society of Chemistry 2025

SUPPORTING INFORMATION

CONTENTS **Page**

Figure S1. ^{1}H NMR Spectrum of Compound 1…… **3**
Figure S2. ^{13}C NMR Spectrum of Compound 1…… **3**
Figure S3. FTIR Spectrum of Compound 1…… **4**
Figure S4. HRMS Spectrum of Compound 1…… **4**
Figure S5. ^{1}H NMR Spectrum of Compound 2…… **5**
Figure S6. ^{13}C NMR Spectrum of Compound 2…… **5**
Figure S7. FTIR Spectrum of Compound 2…… **6**
Figure S8. HRMS Spectrum of Compound 2…… **6**
Figure S9. ^{1}H NMR Spectrum of Compound 3…… **7**
Figure S10. ^{13}C NMR Spectrum of Compound 3…… **7**
Figure S11. FTIR Spectrum of Compound 3…… **8**
Figure S12. HRMS Spectrum of Compound 3…… **8**
Figure S13. ^{1}H NMR Spectrum of Compound 4…… **9**
Figure S14. ^{13}C NMR Spectrum of Compound 4…… **9**
Figure S15. FTIR Spectrum of Compound 4…… **10**
Figure S16. HRMS Spectrum of Compound 4…… **10**
Figure S17. ^{1}H NMR Spectrum of Compound 5…… **11**
Figure S18. ^{13}C NMR Spectrum of Compound 5…… **11**
Figure S19. FTIR Spectrum of Compound 5…… **12**
Figure S20. HRMS Spectrum of Compound 5…… **12**
Figure S21. ^{1}H NMR Spectrum of Compound 6…… **13**
Figure S22. ^{13}C NMR Spectrum of Compound 6…… **13**
Figure S23. FTIR Spectrum of Compound 6…… **14**
Figure S24. HRMS Spectrum of Compound 6…… **14**
Figure S25. ^{1}H NMR Spectrum of Compound 7…… **15**
Figure S26. ^{13}C NMR Spectrum of Compound 7…… **15**
Figure S27. FTIR Spectrum of Compound 7…… **16**
Figure S28. HRMS Spectrum of Compound 7…… **16**
Figure S29. ^{1}H NMR Spectrum of Compound 8…… **17**
Figure S30. ^{13}C NMR Spectrum of Compound 8…… **17**
Figure S31. FTIR Spectrum of Compound 8…… **18**
Figure S32. HRMS Spectrum of Compound 8…… **18**
Figure S33. ^{1}H NMR Spectrum of Compound 9…… **19**
Figure S34. ^{13}C NMR Spectrum of Compound 9…… **19**
Figure S35. FTIR Spectrum of Compound 9…… **20**
Figure S36. HRMS Spectrum of Compound 9…… **20**
Figure S37. ^{1}H NMR Spectrum of Compound 10…… **21**
Figure S38. ^{13}C NMR Spectrum of Compound 10…… **21**
Figure S39. FTIR Spectrum of Compound 10…… **22**
Figure S40. HRMS Spectrum of Compound 10…… **22**
Figure S41. ^{1}H NMR Spectrum of Compound 11…… **23**
Figure S42. ^{13}C NMR Spectrum of Compound 11…… **23**
Figure S43. FTIR Spectrum of Compound 11…… **24**
Figure S44. HRMS Spectrum of Compound 11…… **24**
Figure S45. ^{1}H NMR Spectrum of Compound 12…… **25**
Figure S46. ^{13}C NMR Spectrum of Compound 12…… **25**
Figure S47. FTIR Spectrum of Compound 12…… **26**
Figure S48. HRMS Spectrum of Compound 12…… **26**
Figure S49. ^{1}H NMR Spectrum of Compound 13…… **27**

Figure S50. ^{13}C NMR Spectrum of Compound 13………………………………………………….. **27**
Figure S51. FTIR Spectrum of Compound 13…………………………………………………… **28**
Figure S52. HRMS Spectrum of Compound 13………………………………………………….. **28**
Figure S53. Lineweaver-Burk plots of compound 1 for AChE…………………………………… **29**
Figure S54. Lineweaver-Burk plots of compound 2 for AChE…………………………………… **29**
Figure S55. Lineweaver-Burk plots of compound 3 for AChE…………………………………… **29**
Figure S56. Lineweaver-Burk plots of compound 4 for AChE…………………………………… **30**
Figure S57. Lineweaver-Burk plots of compound 5 for AChE…………………………………… **30**
Figure S58. Lineweaver-Burk plots of compound 6 for AChE…………………………………… **30**
Figure S59. Lineweaver-Burk plots of compound 7 for AChE…………………………………… **31**
Figure S60. Lineweaver-Burk plots of compound 8 for AChE…………………………………… **31**
Figure S61. Lineweaver-Burk plots of compound 9 for AChE…………………………………… **31**
Figure S62. Lineweaver-Burk plots of compound 10 for AChE………………………………….. **32**
Figure S63. Lineweaver-Burk plots of compound 11 for AChE………………………………….. **32**
Figure S64. Lineweaver-Burk plots of compound 12 for AChE………………………………….. **32**
Figure S65. Lineweaver-Burk plots of compound 13 for AChE………………………………….. **33**
Figure S66. Lineweaver-Burk plots of compound 1 for BChE…………………………………… **33**
Figure S67. Lineweaver-Burk plots of compound 2 for BChE…………………………………… **33**
Figure S68. Lineweaver-Burk plots of compound 3 for BChE…………………………………… **34**
Figure S69. Lineweaver-Burk plots of compound 4 for BChE…………………………………… **34**
Figure S70. Lineweaver-Burk plots of compound 5 for BChE…………………………………… **34**
Figure S71. Lineweaver-Burk plots of compound 6 for BChE…………………………………… **35**
Figure S72. Lineweaver-Burk plots of compound 7 for BChE…………………………………… **35**
Figure S73. Lineweaver-Burk plots of compound 8 for BChE…………………………………… **35**
Figure S74. Lineweaver-Burk plots of compound 9 for BChE…………………………………… **36**
Figure S75. Lineweaver-Burk plots of compound 10 for BChE………………………………….. **36**
Figure S76. Lineweaver-Burk plots of compound 11 for BChE………………………………….. **36**
Figure S77. Lineweaver-Burk plots of compound 12 for BChE………………………………….. **37**
Figure S78. Lineweaver-Burk plots of compound 13 for BChE………………………………….. **37**
Figure S79. IC_{50} Graph of compound 4 for HUVEC…………………………………………….. **38**
Figure S80. IC_{50} Graph of compound 6 for HUVEC…………………………………………….. **38**
Figure S81. IC_{50} Graph of compound 7 for HUVEC…………………………………………….. **38**
Figure S82. IC_{50} Graph of compound 10 for HUVEC…………………………………………… **39**
Figure S83. IC_{50} Graph of compound 11 for HUVEC…………………………………………… **39**

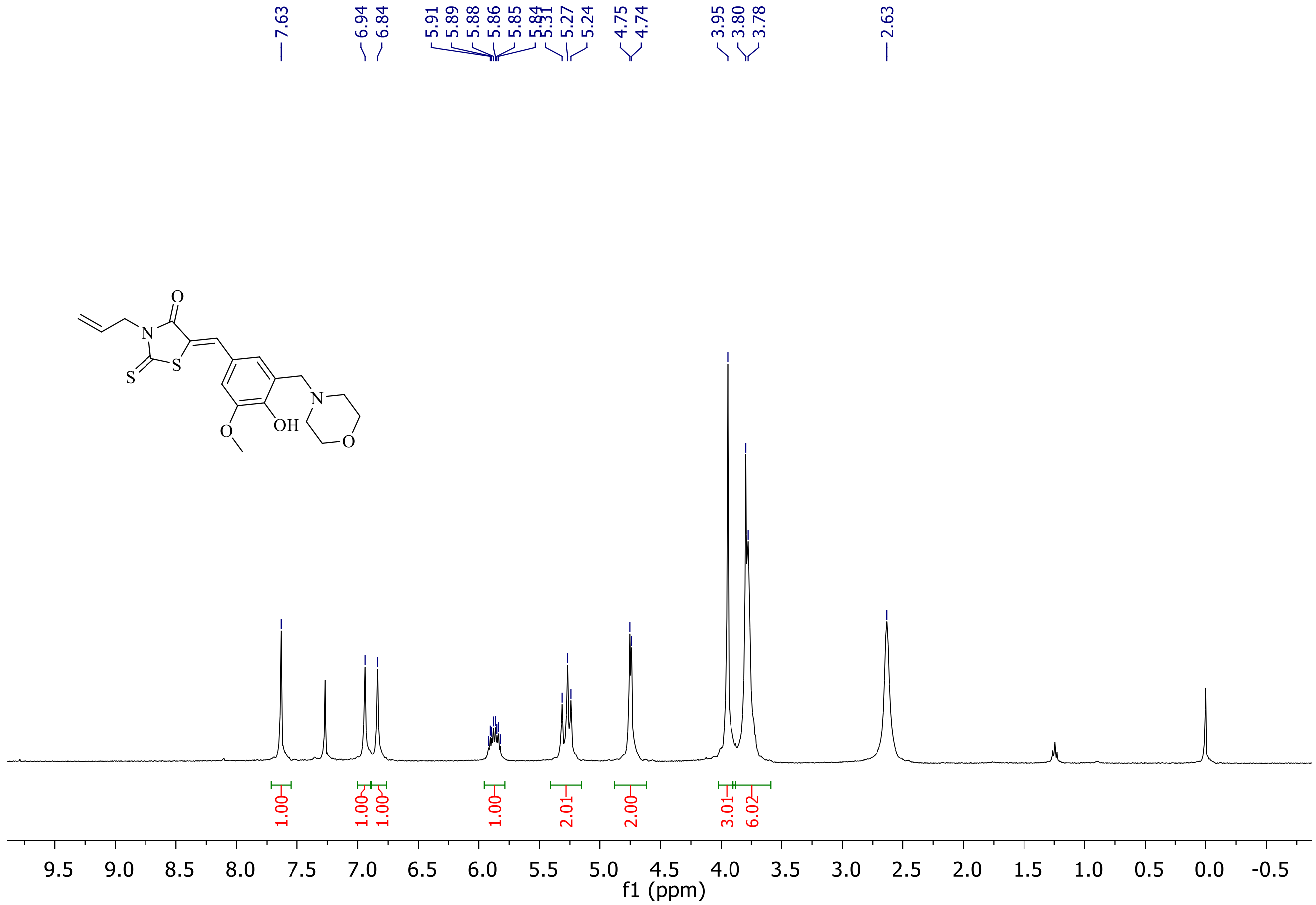

Figure S1. ^{1}H NMR Spectrum of Compound 1

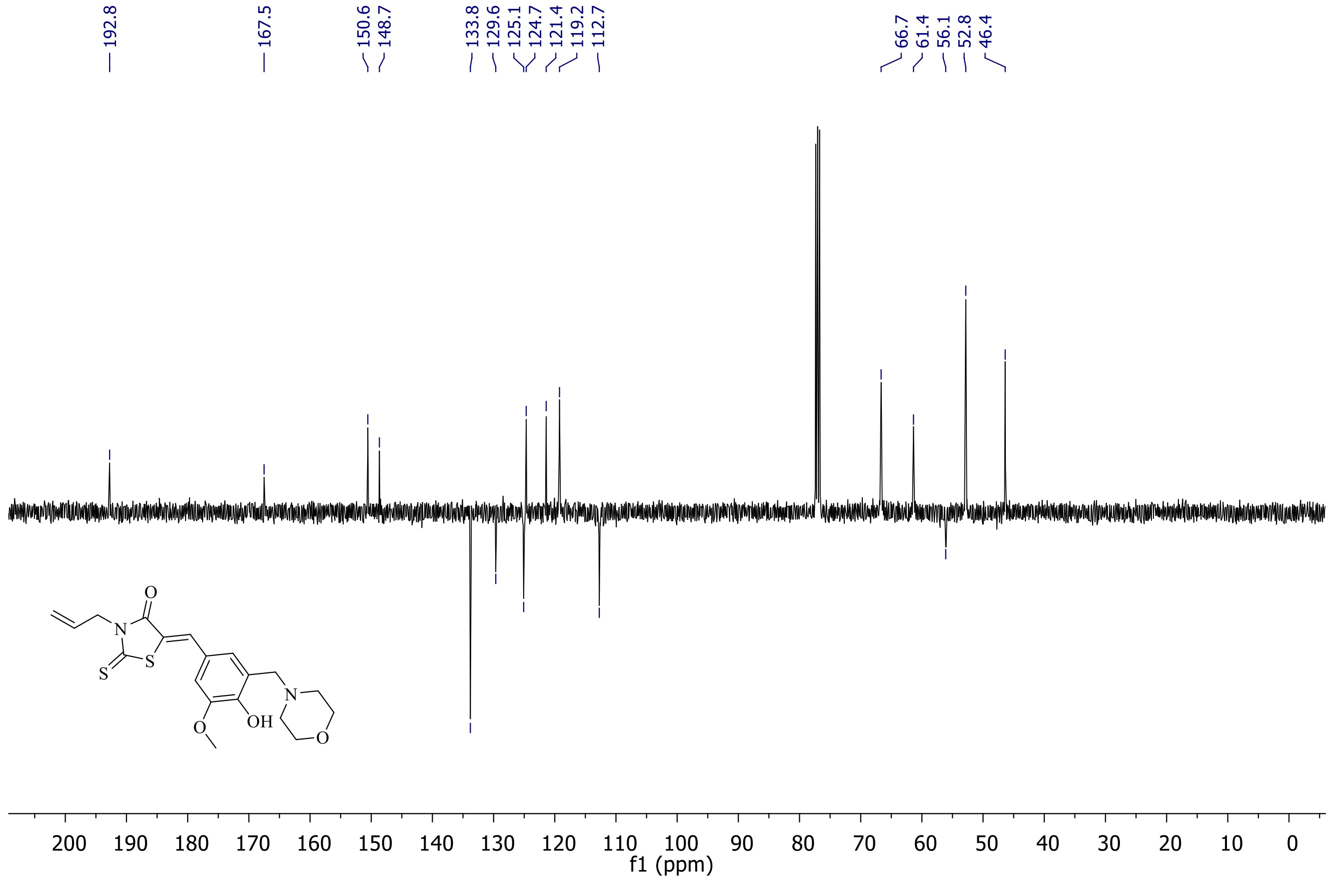

Figure S2. ^{13}C NMR Spectrum of Compound 1

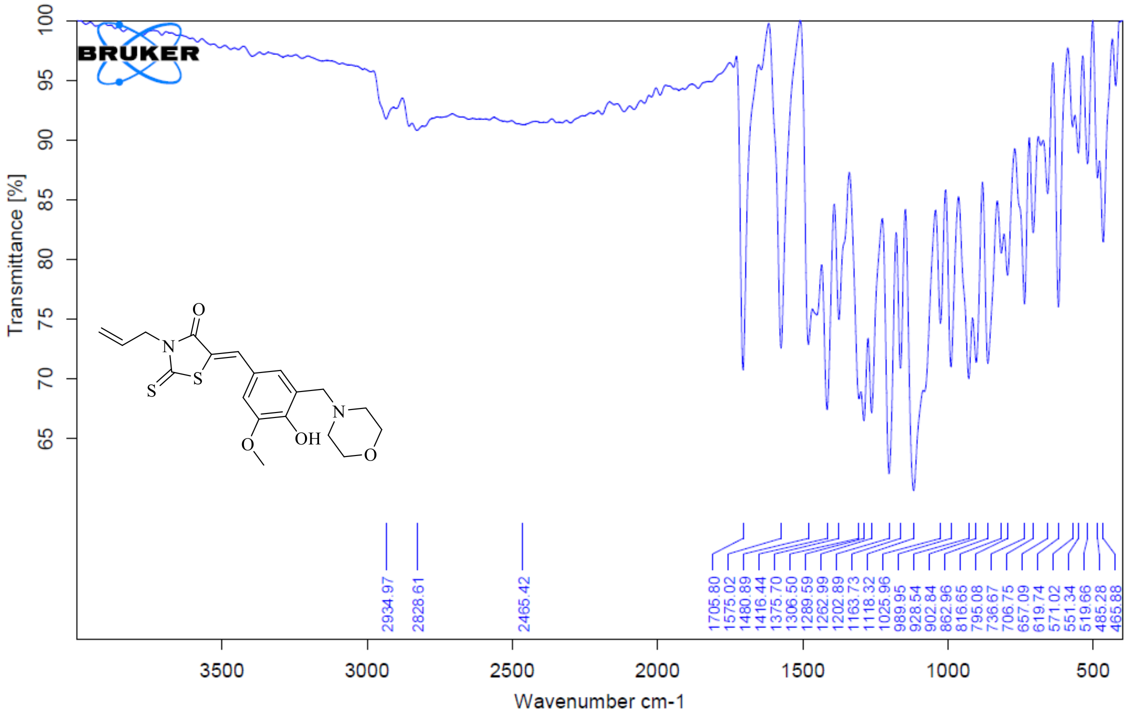

Figure S3. FTIR Spectrum of Compound 1

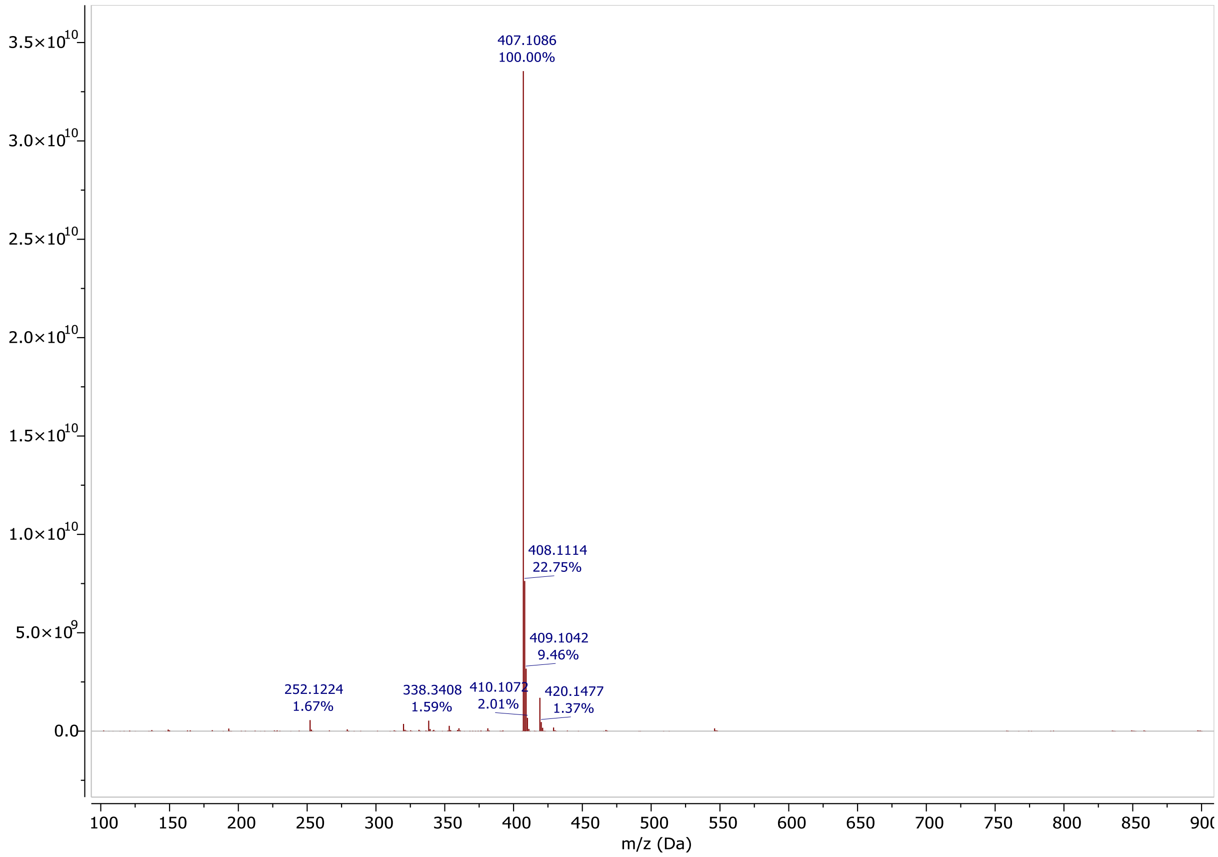

Figure S4. HRMS Spectrum of Compound 1

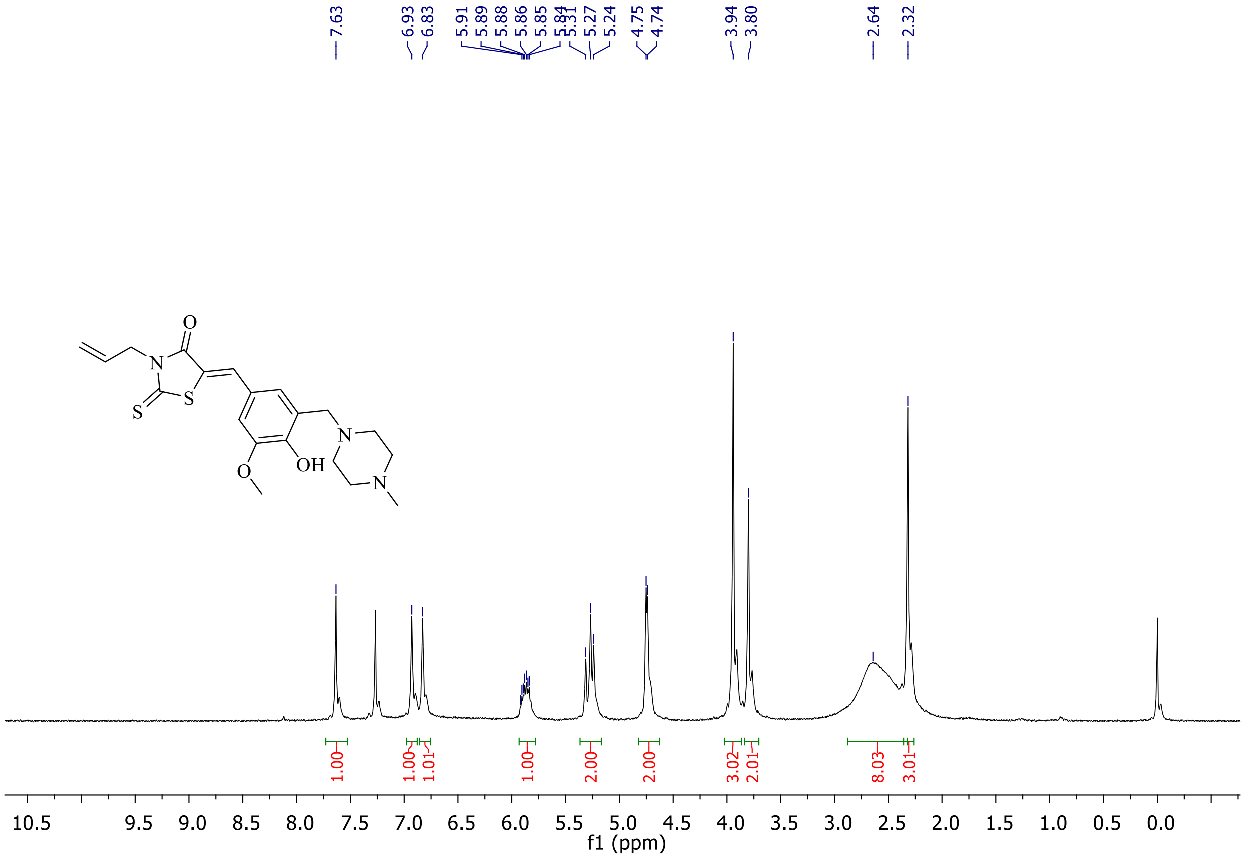

Figure S5. ^{1}H NMR Spectrum of Compound 2

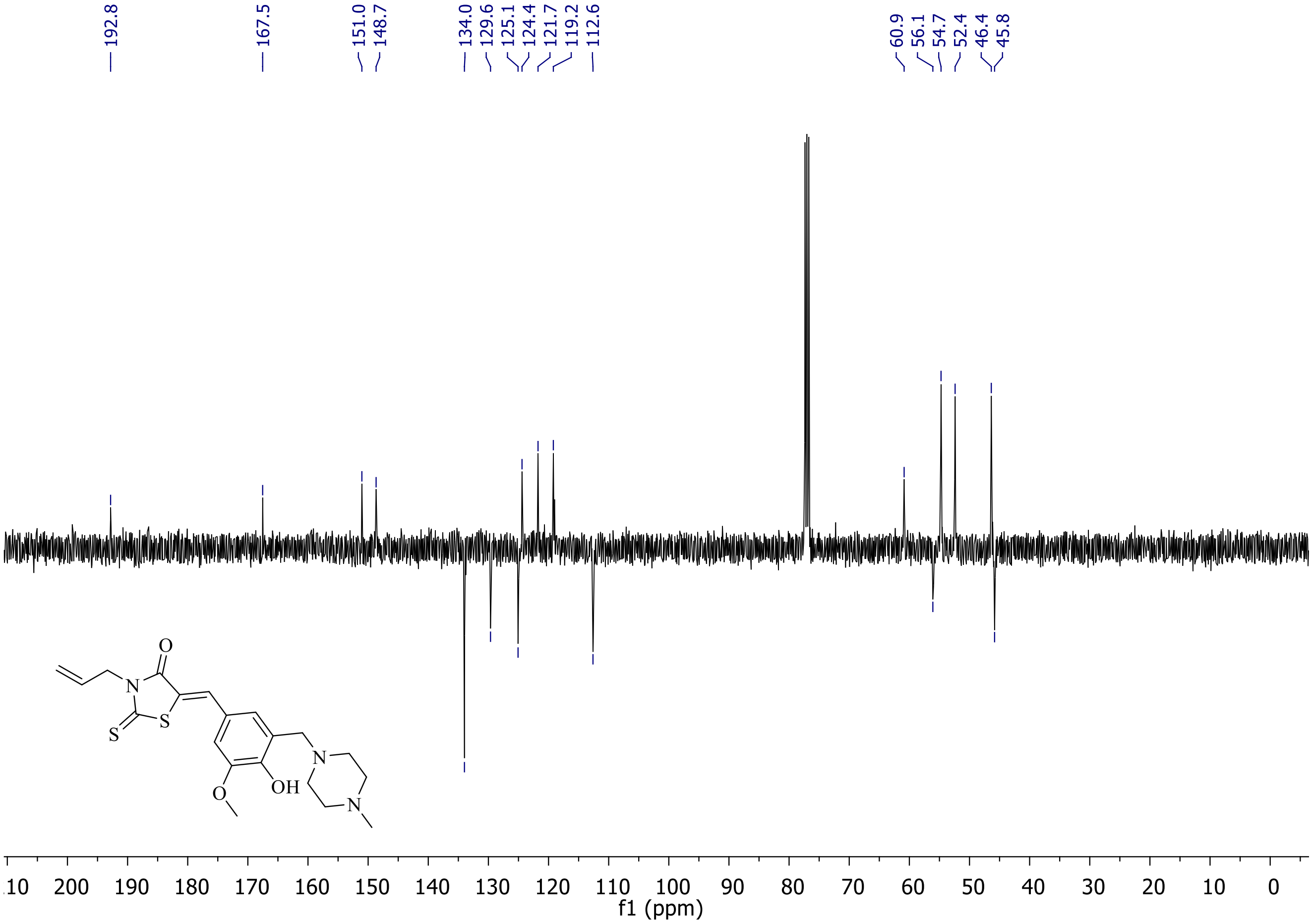

Figure S6. ^{13}C NMR Spectrum of Compound 2

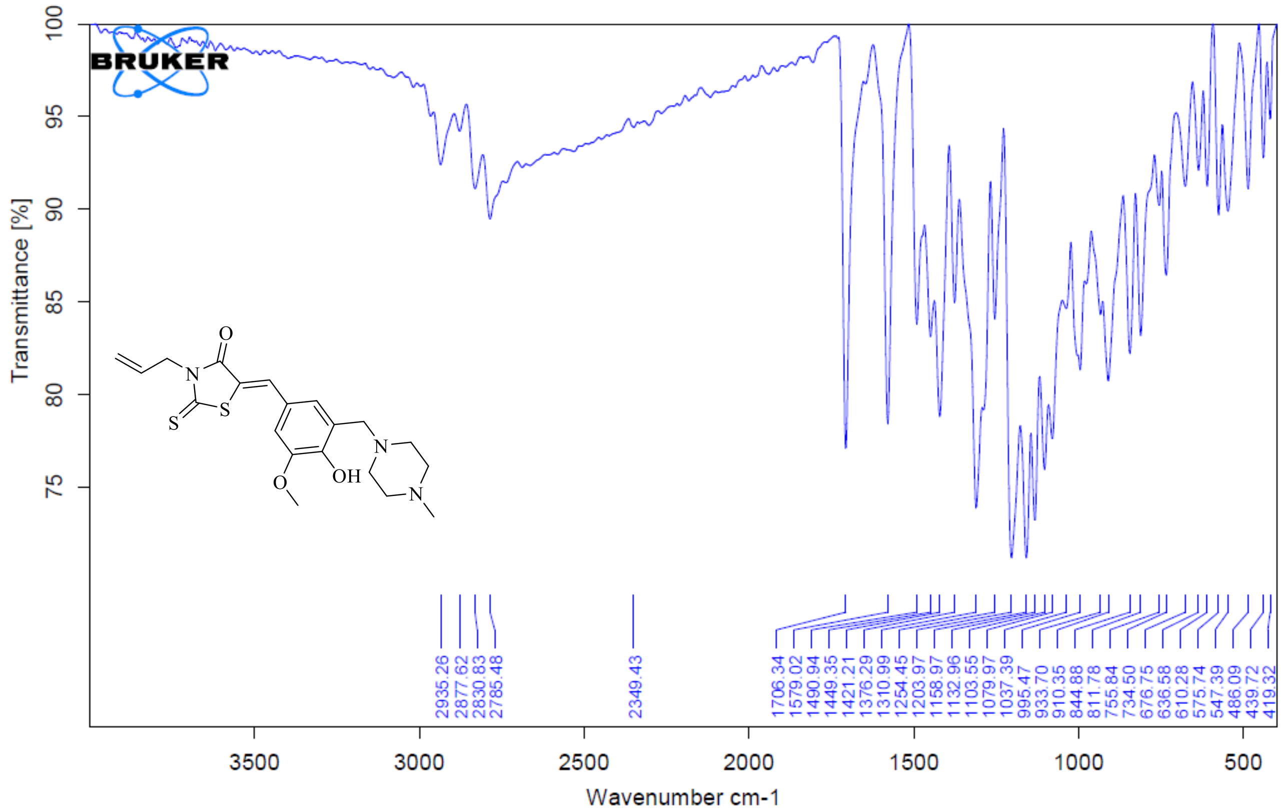

Figure S7. FTIR Spectrum of Compound 2

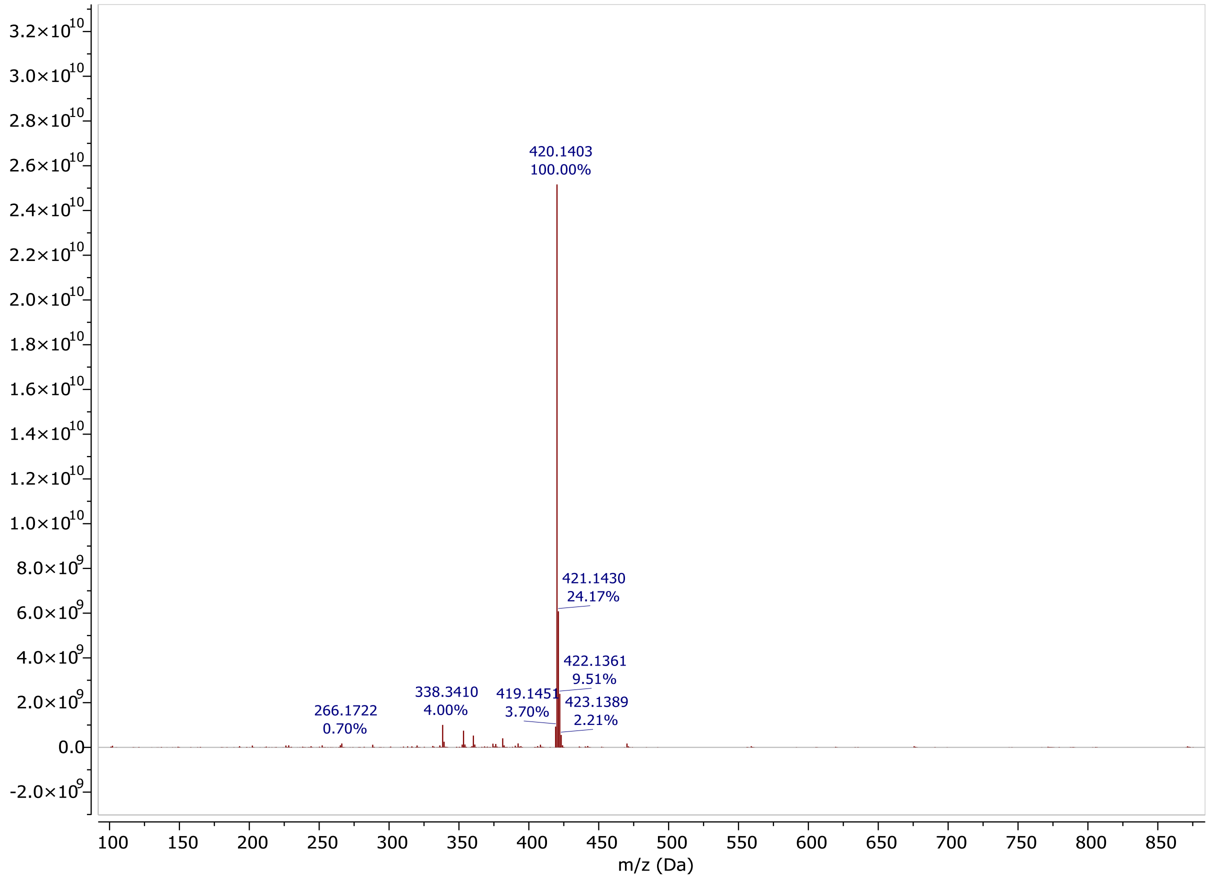

Figure S8. HRMS Spectrum of Compound 2

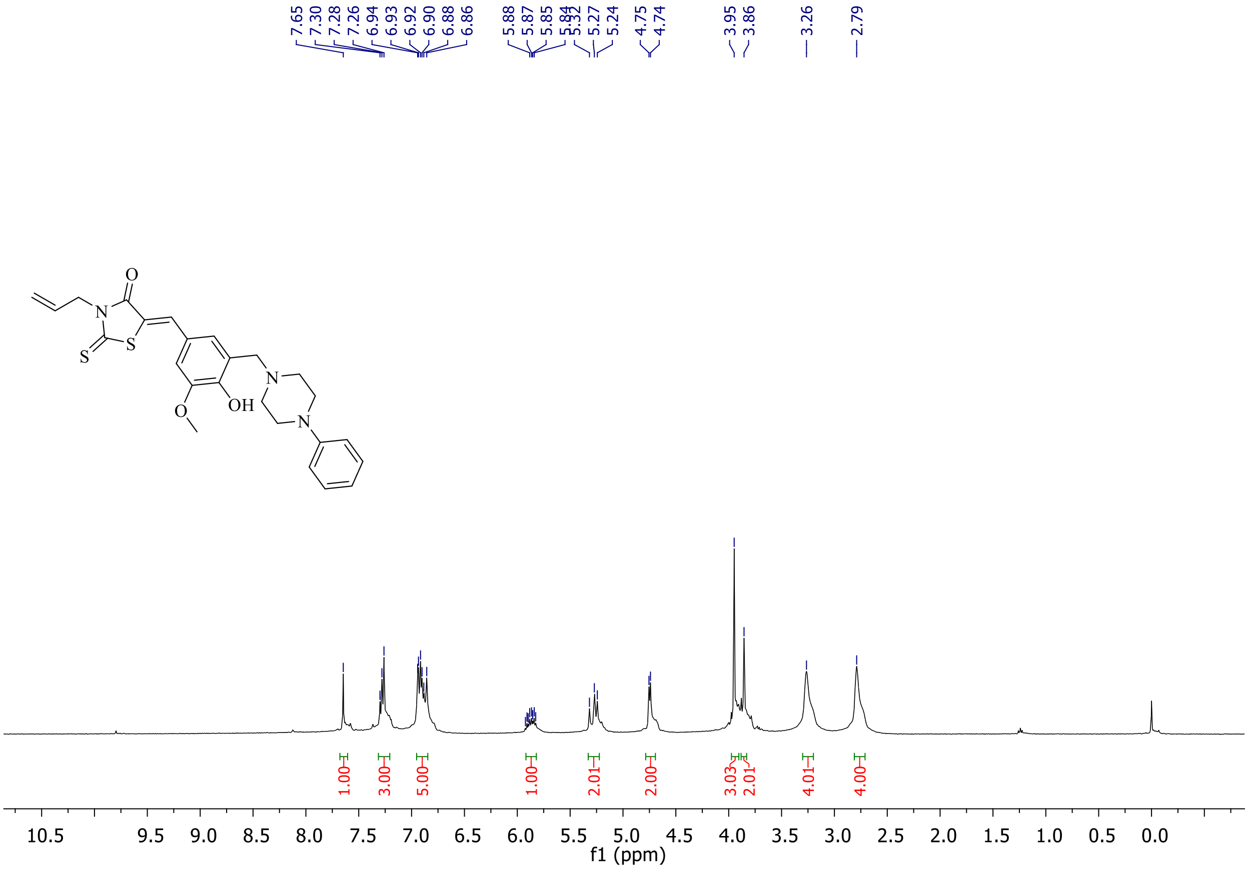

Figure S9. ^{1}H NMR Spectrum of Compound 3

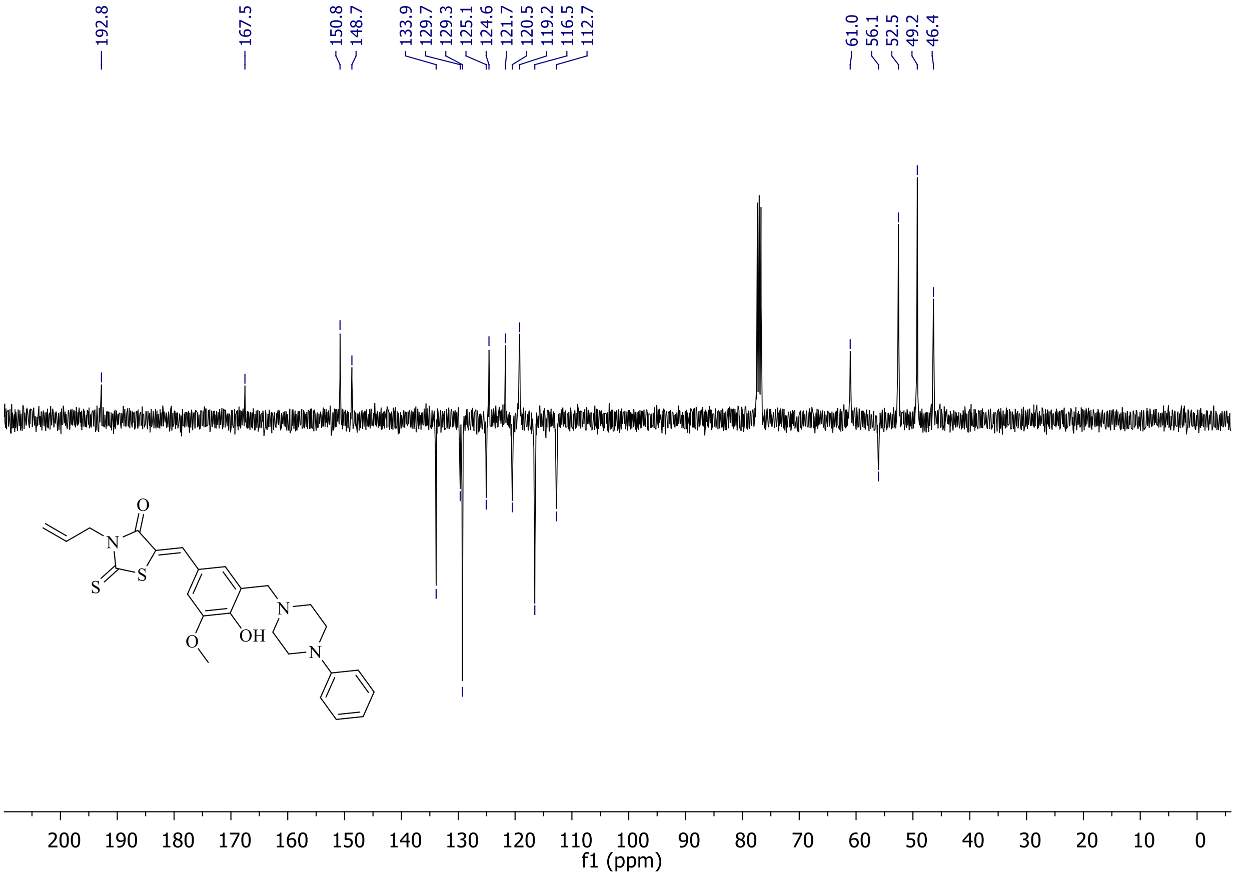

Figure S10. ^{13}C NMR Spectrum of Compound 3

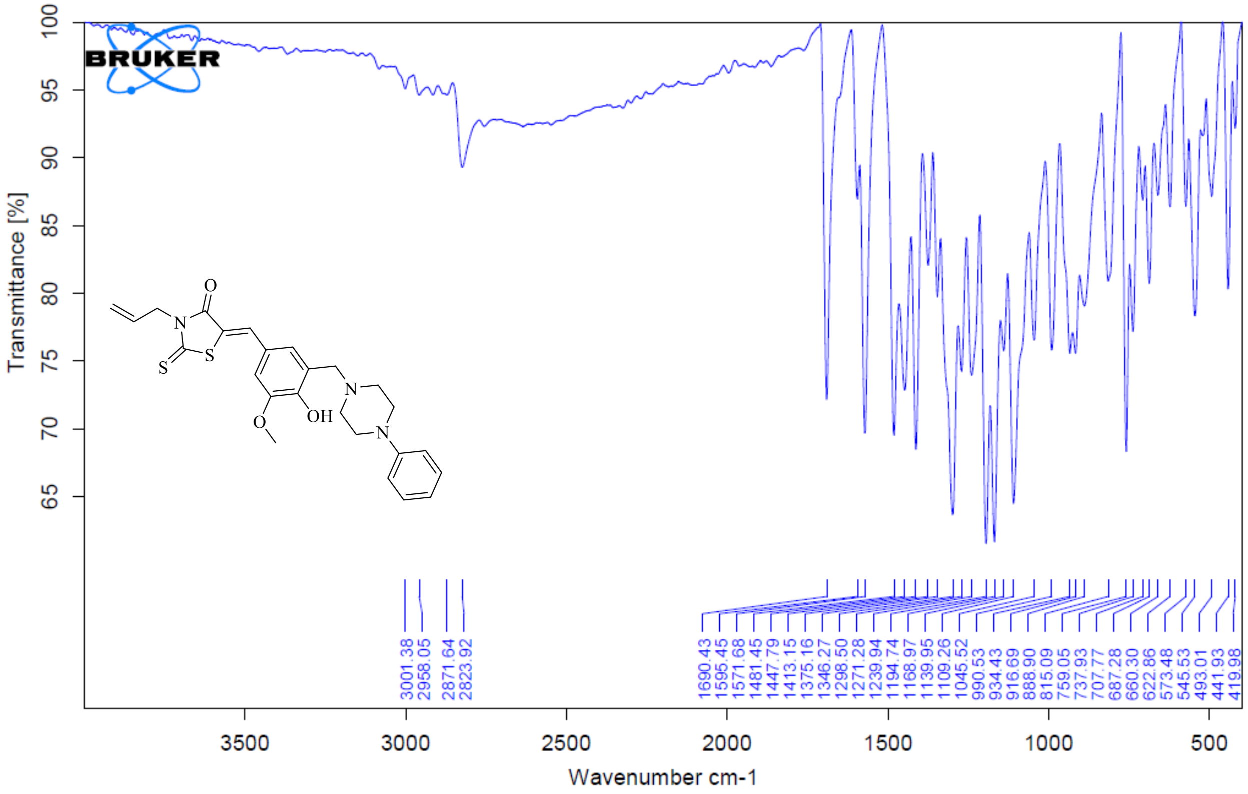

Figure S11. FTIR Spectrum of Compound 3

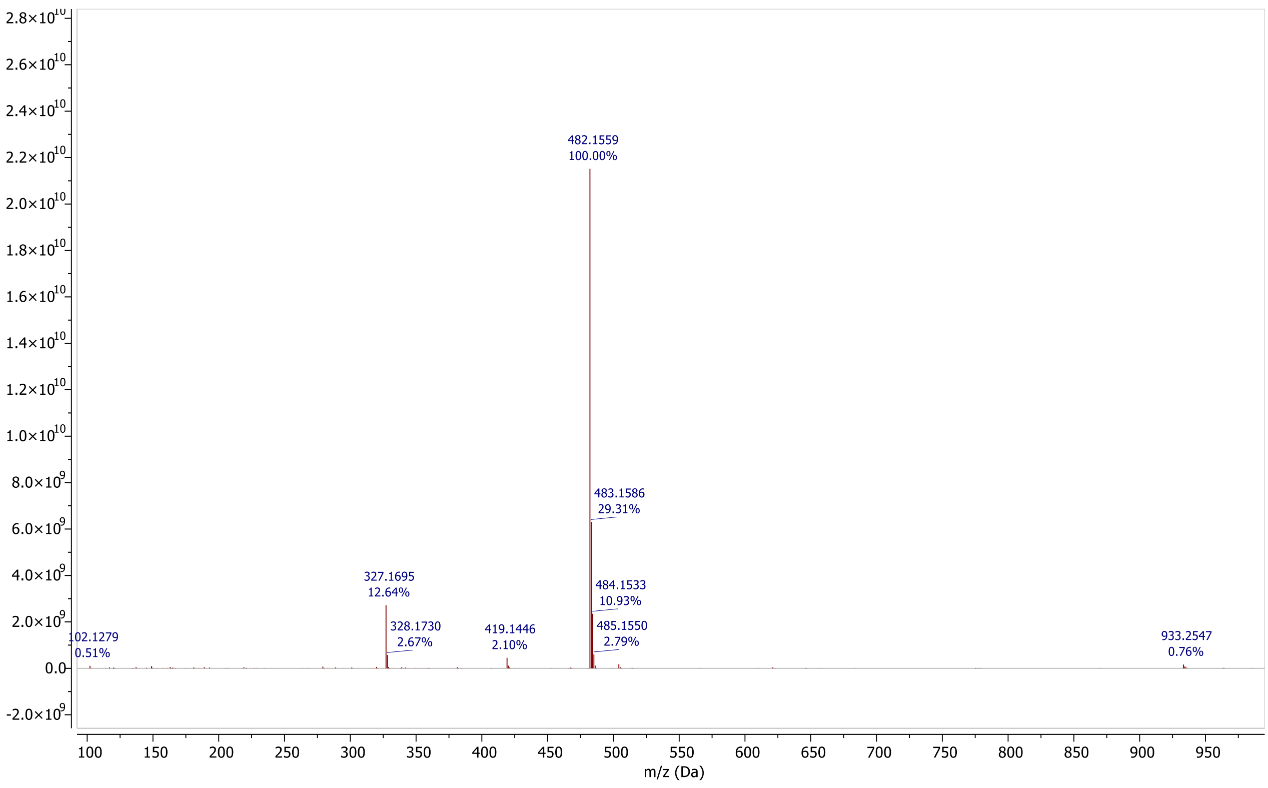

Figure S2. HRMS Spectrum of Compound 3

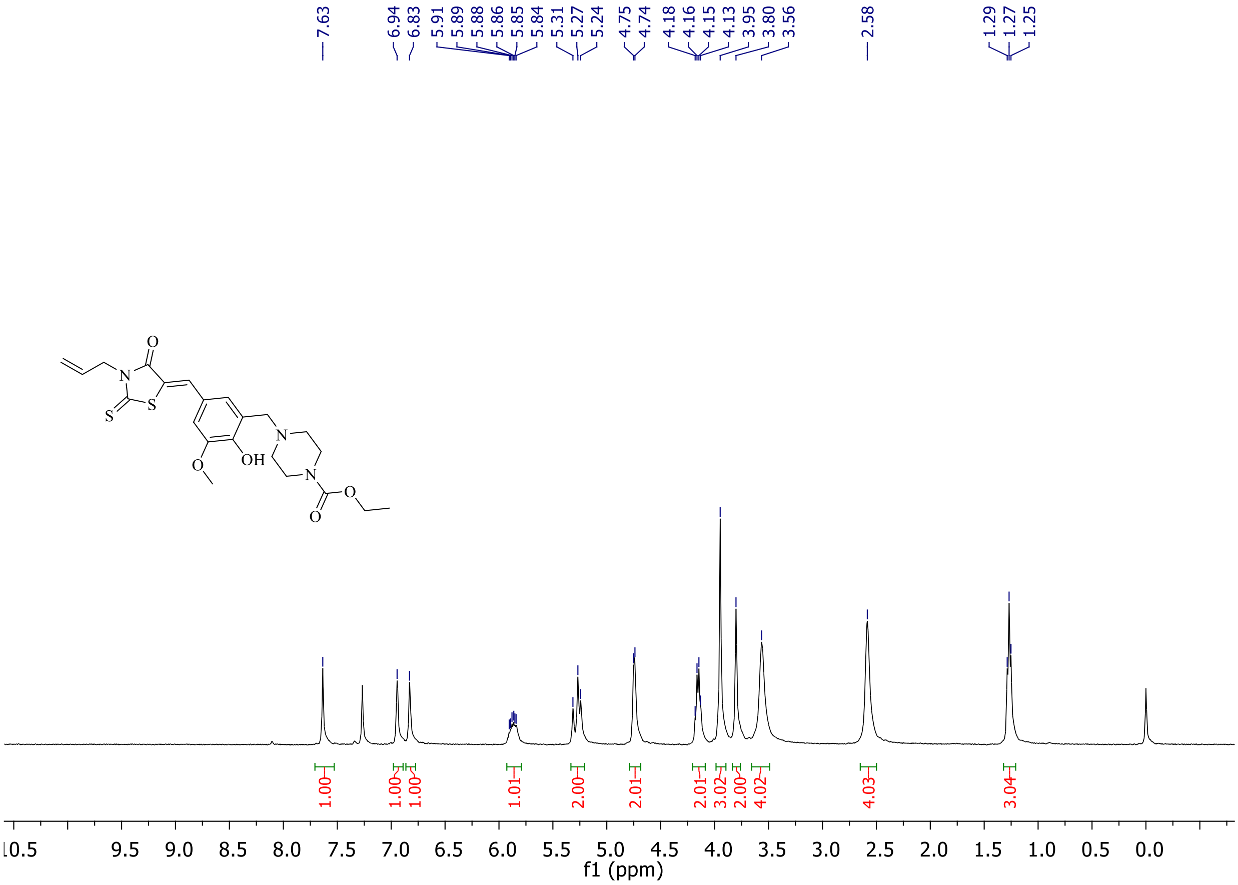

Figure S13. ^{1}H NMR Spectrum of Compound 4

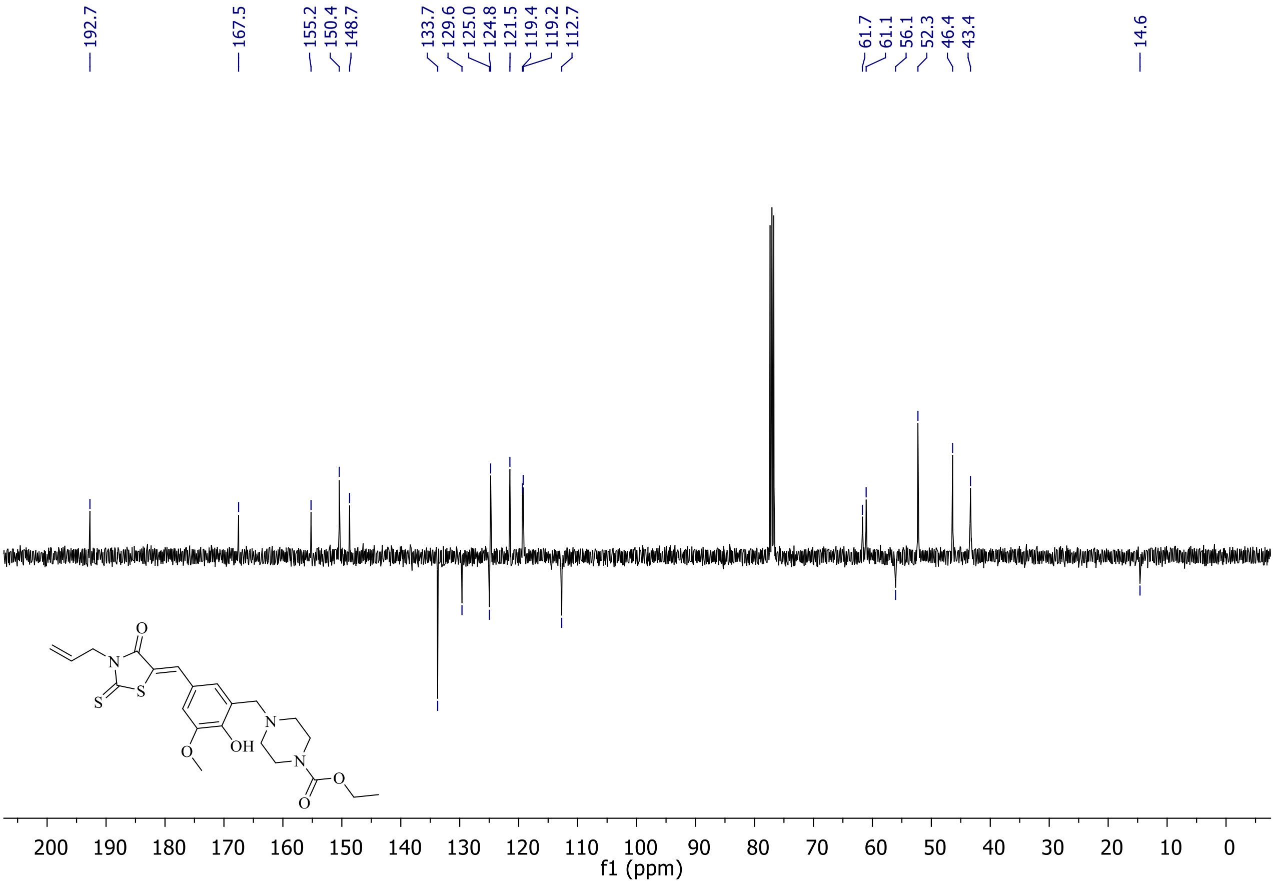

Figure S14. ^{13}C NMR Spectrum of Compound 4

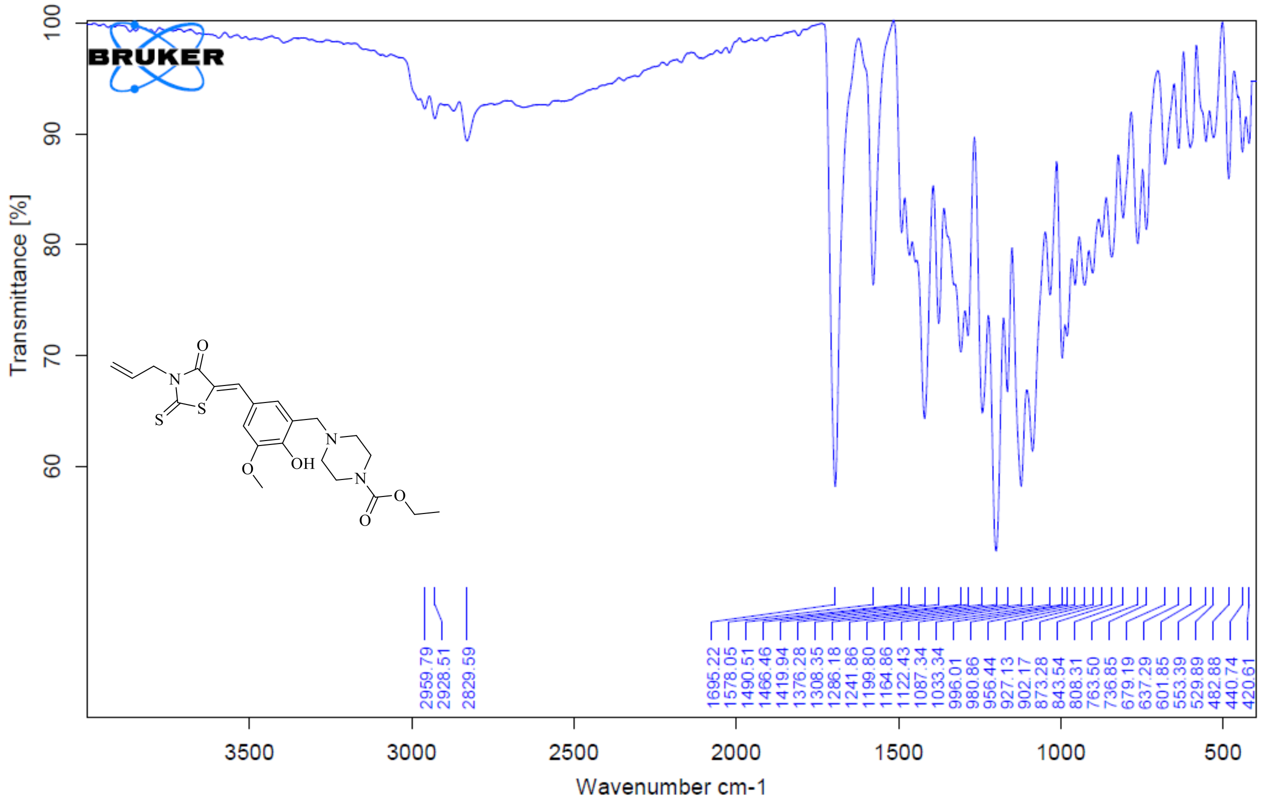

Figure S15. FTIR Spectrum of Compound 4

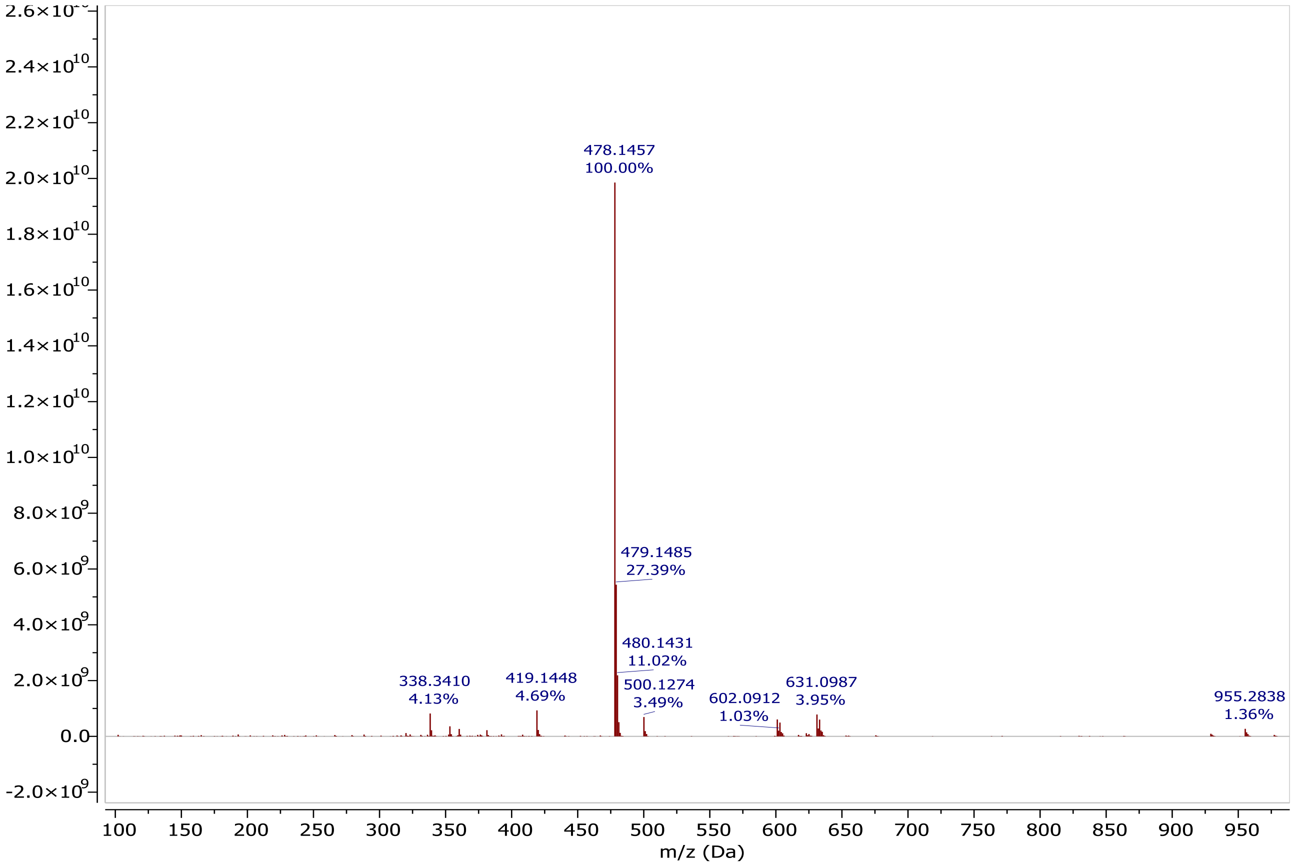

Figure S16. HRMS Spectrum of Compound 4

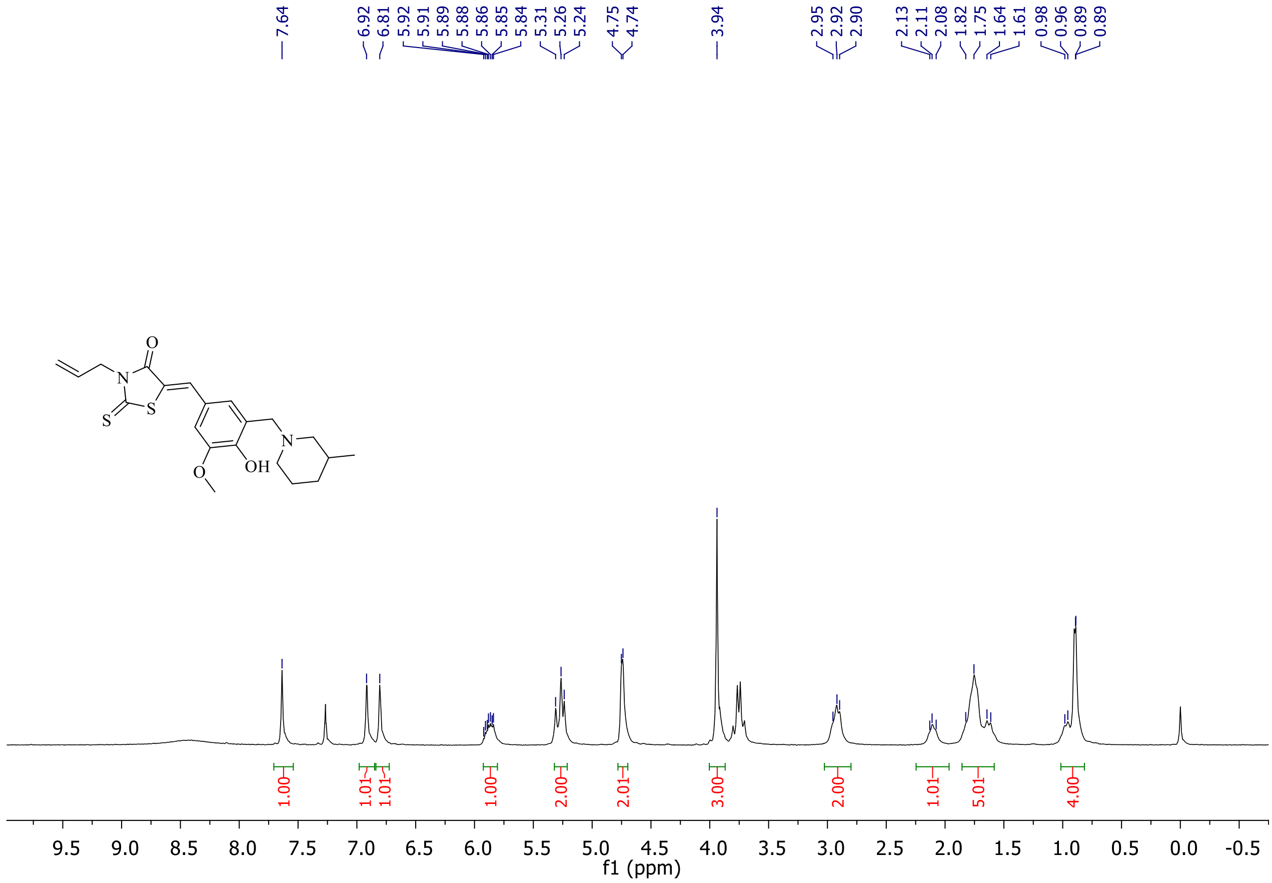

Figure S17. ^{1}H NMR Spectrum of Compound 5

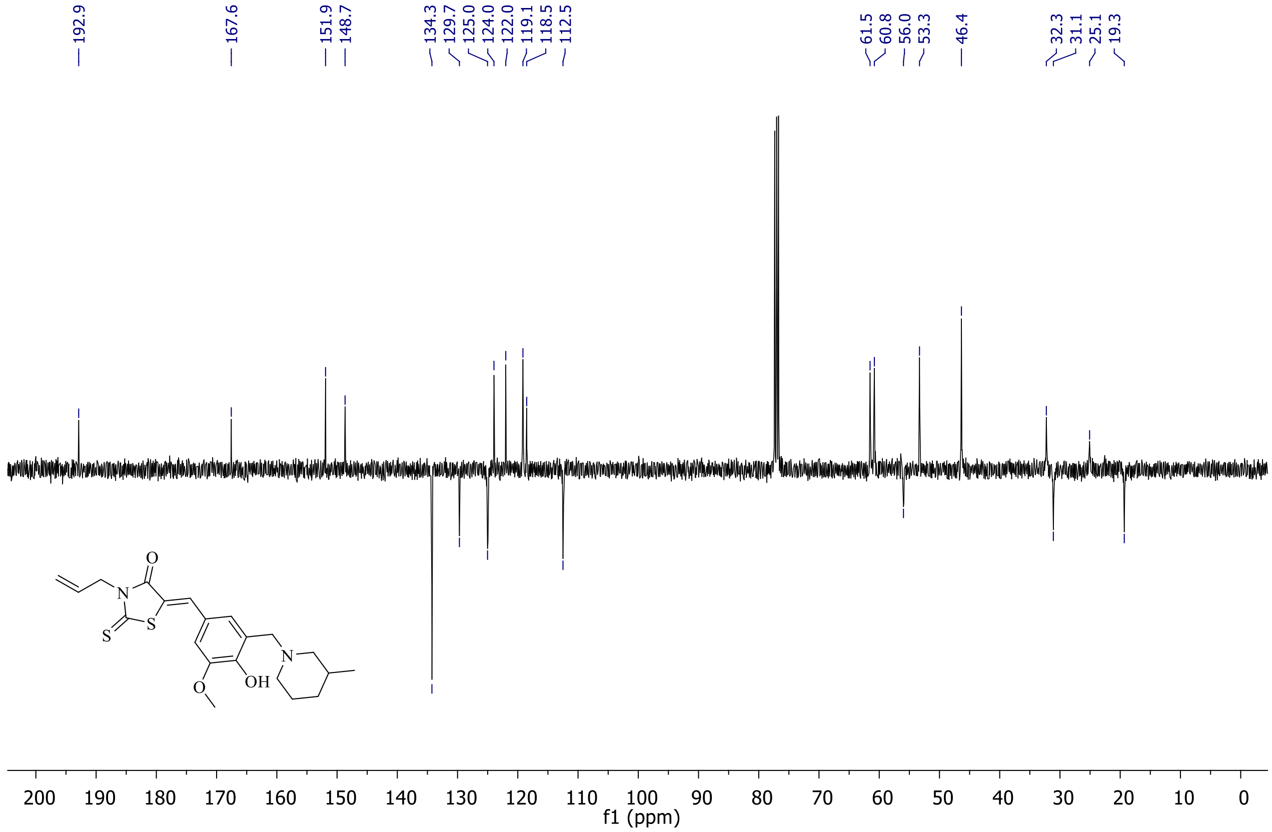

Figure S18. ^{13}C NMR Spectrum of Compound 5

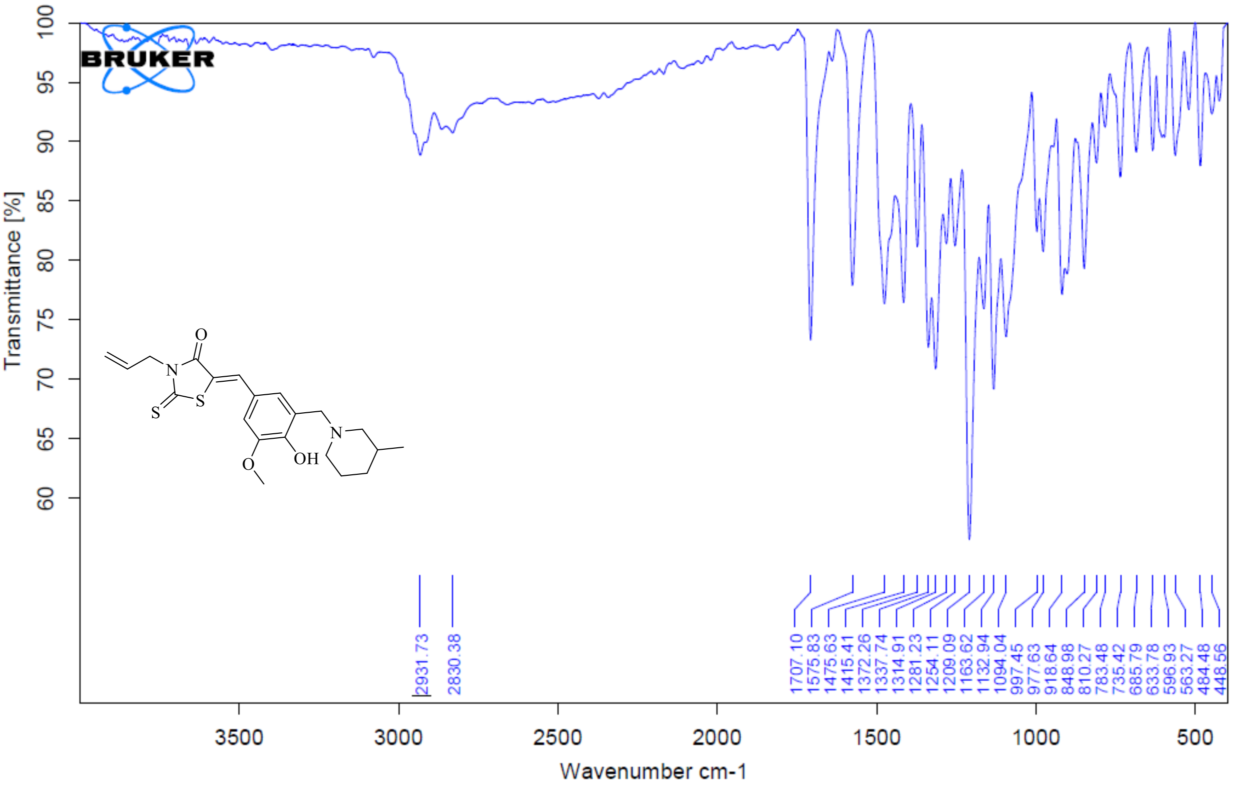

Figure S19. FTIR Spectrum of Compound 5

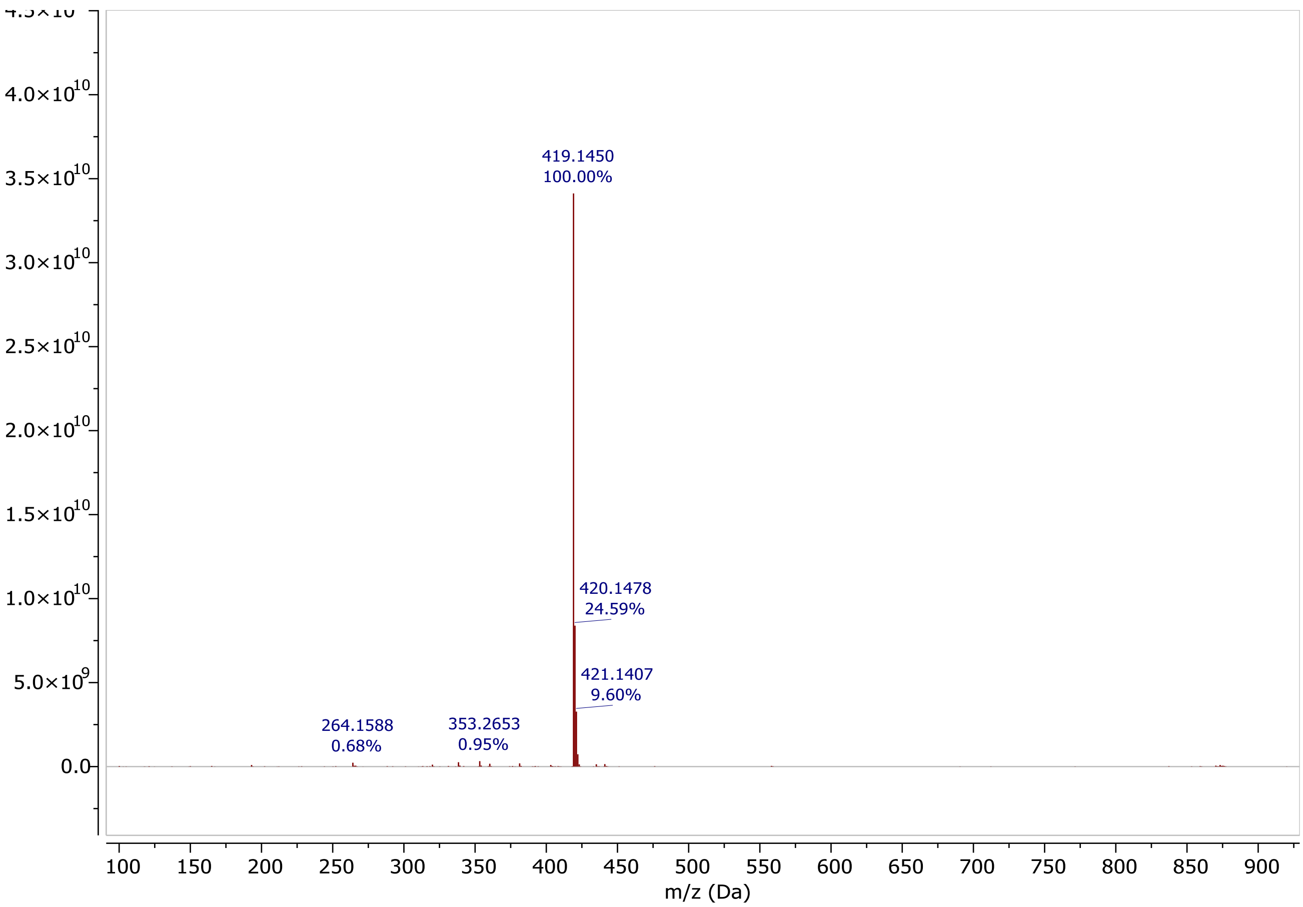

Figure S20. HRMS Spectrum of Compound 5

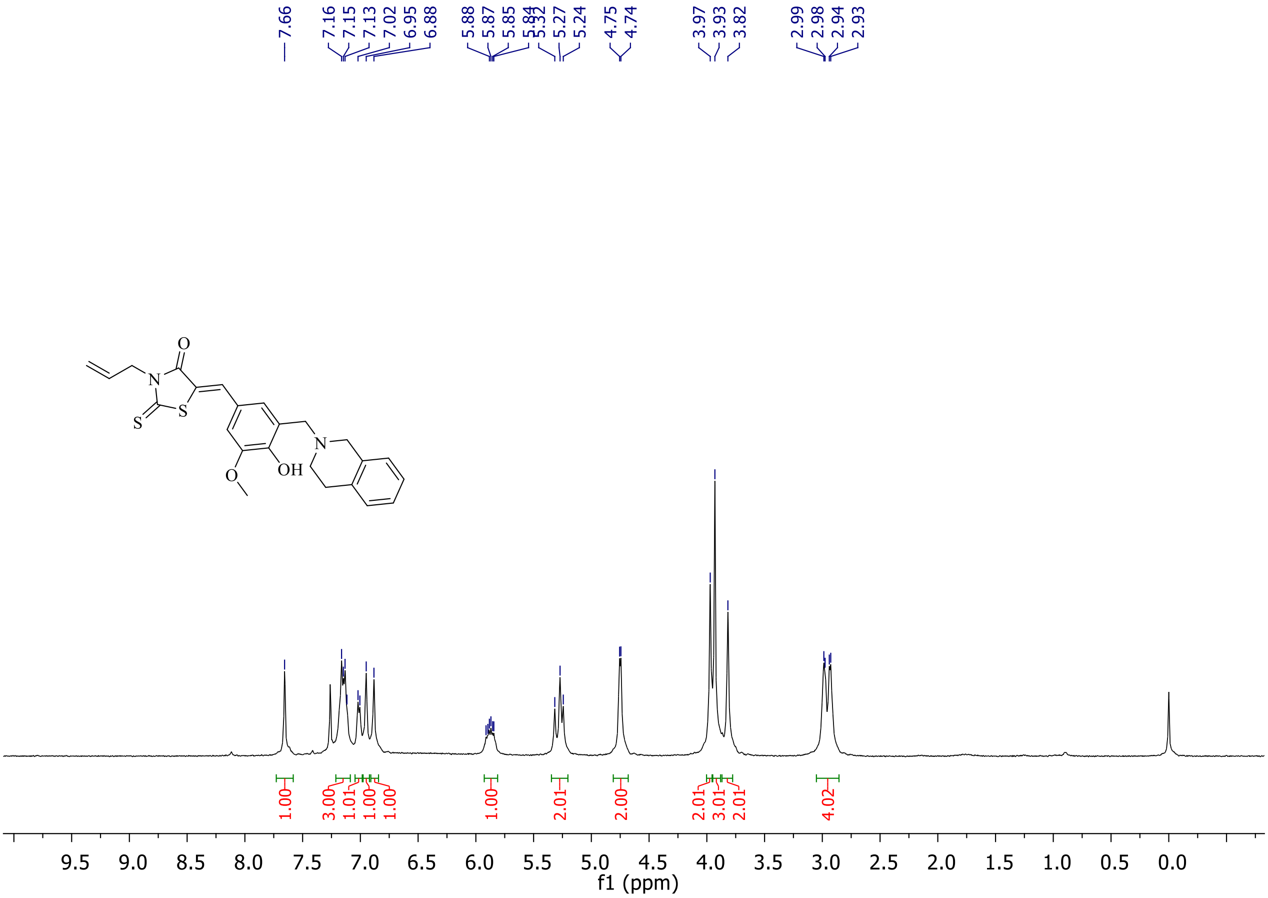

Figure S21. ^{1}H NMR Spectrum of Compound 6

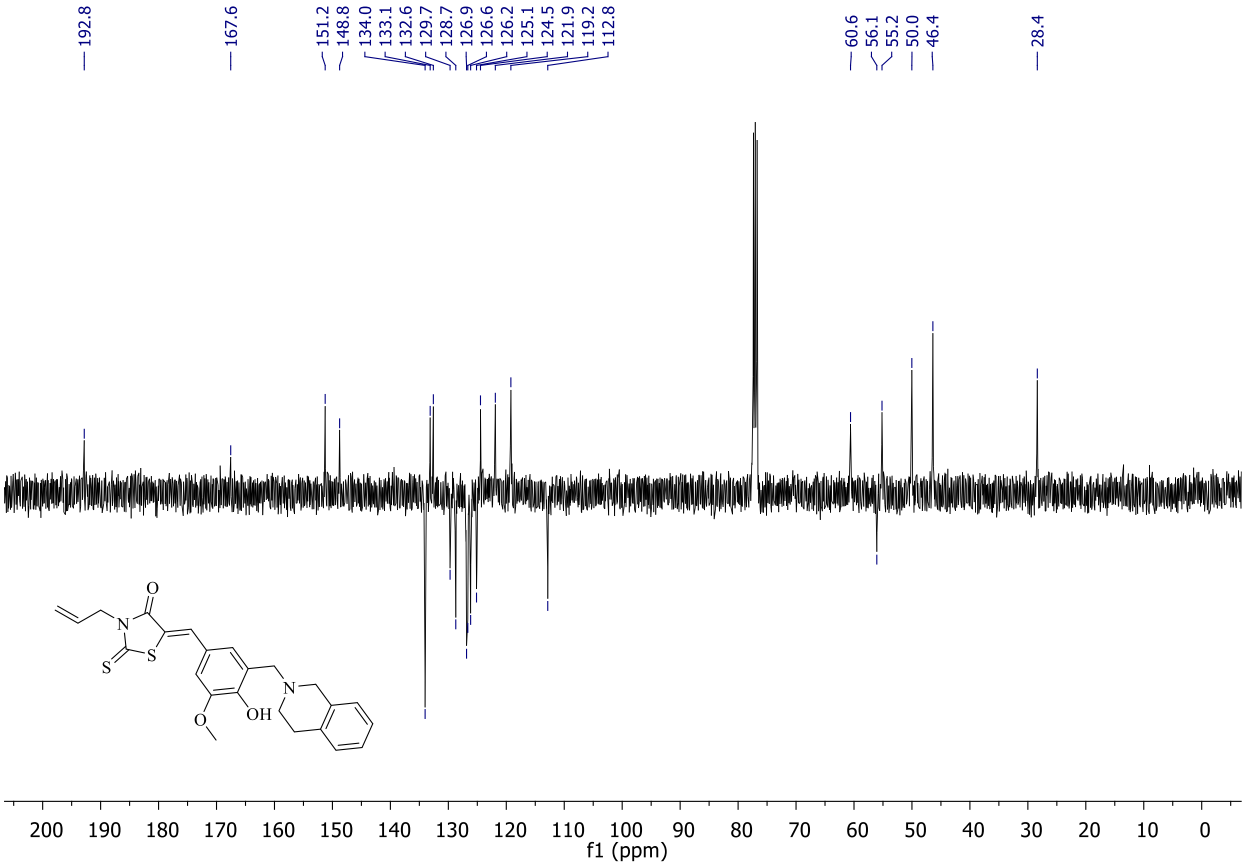

Figure S22. ^{13}C NMR Spectrum of Compound 6

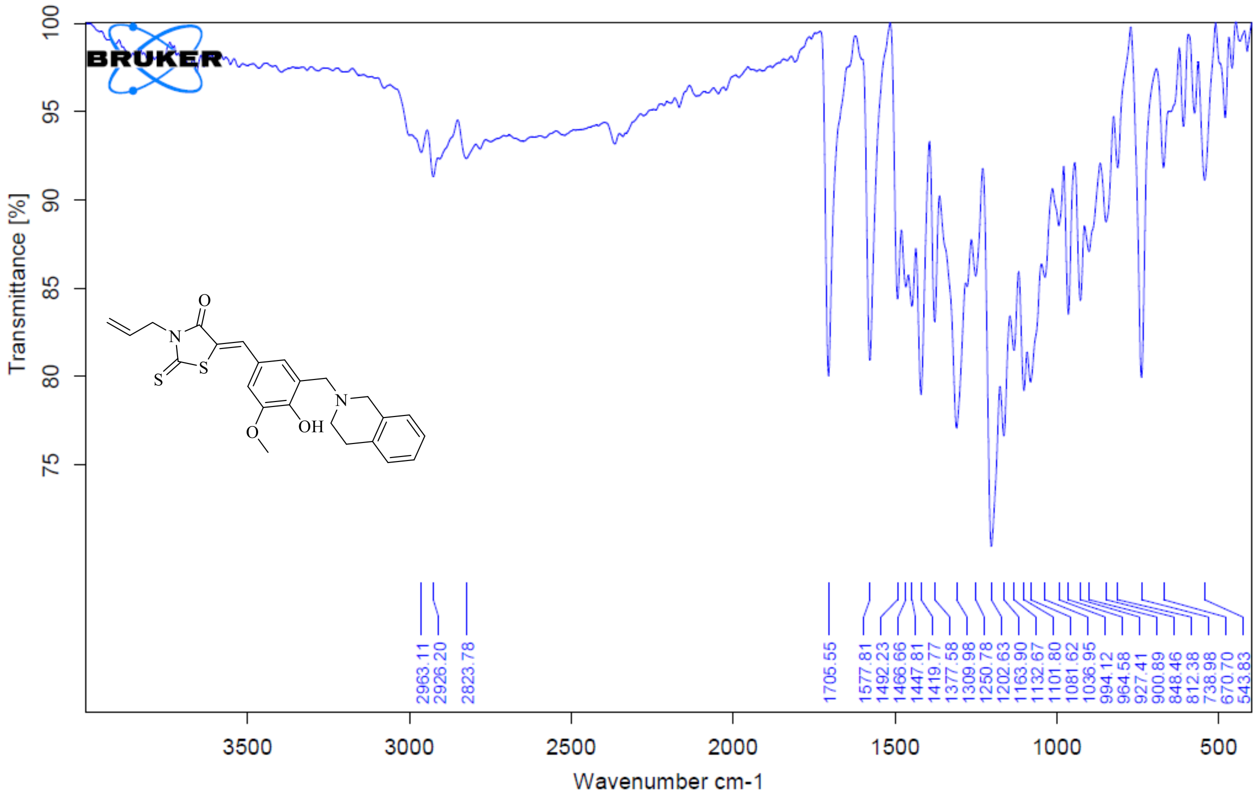

Figure S23. FTIR Spectrum of Compound 6

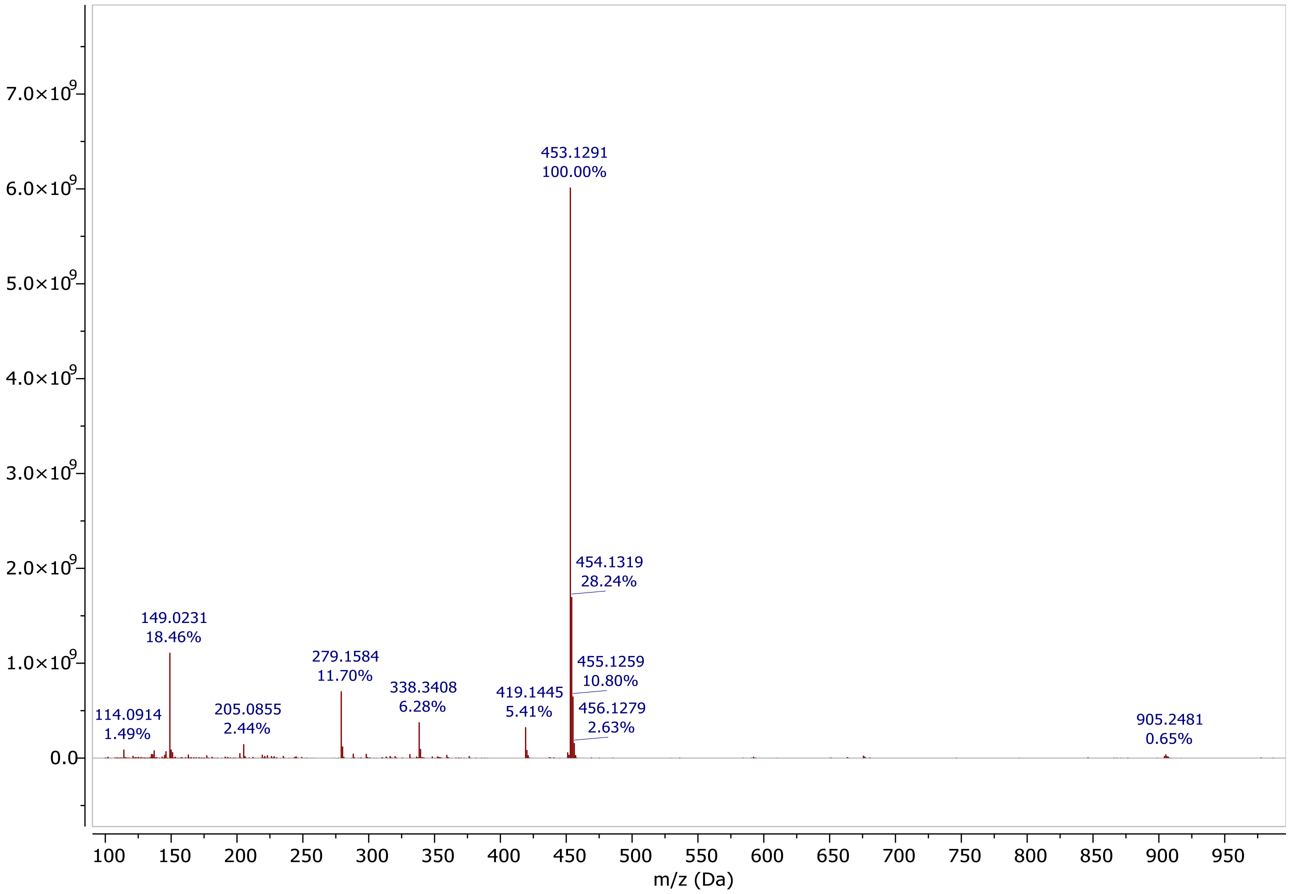

Figure S24. HRMS Spectrum of Compound 6

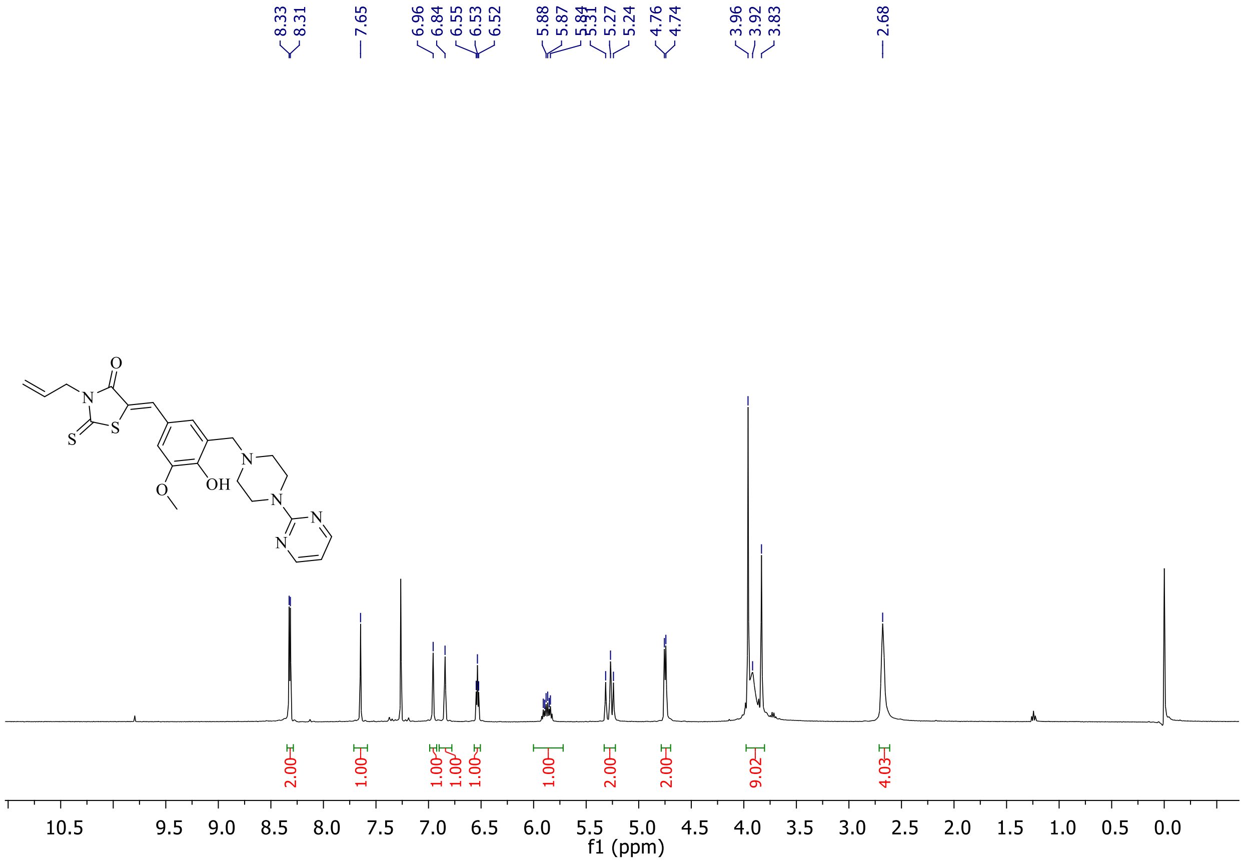

Figure S25. ^{1}H NMR Spectrum of Compound 7

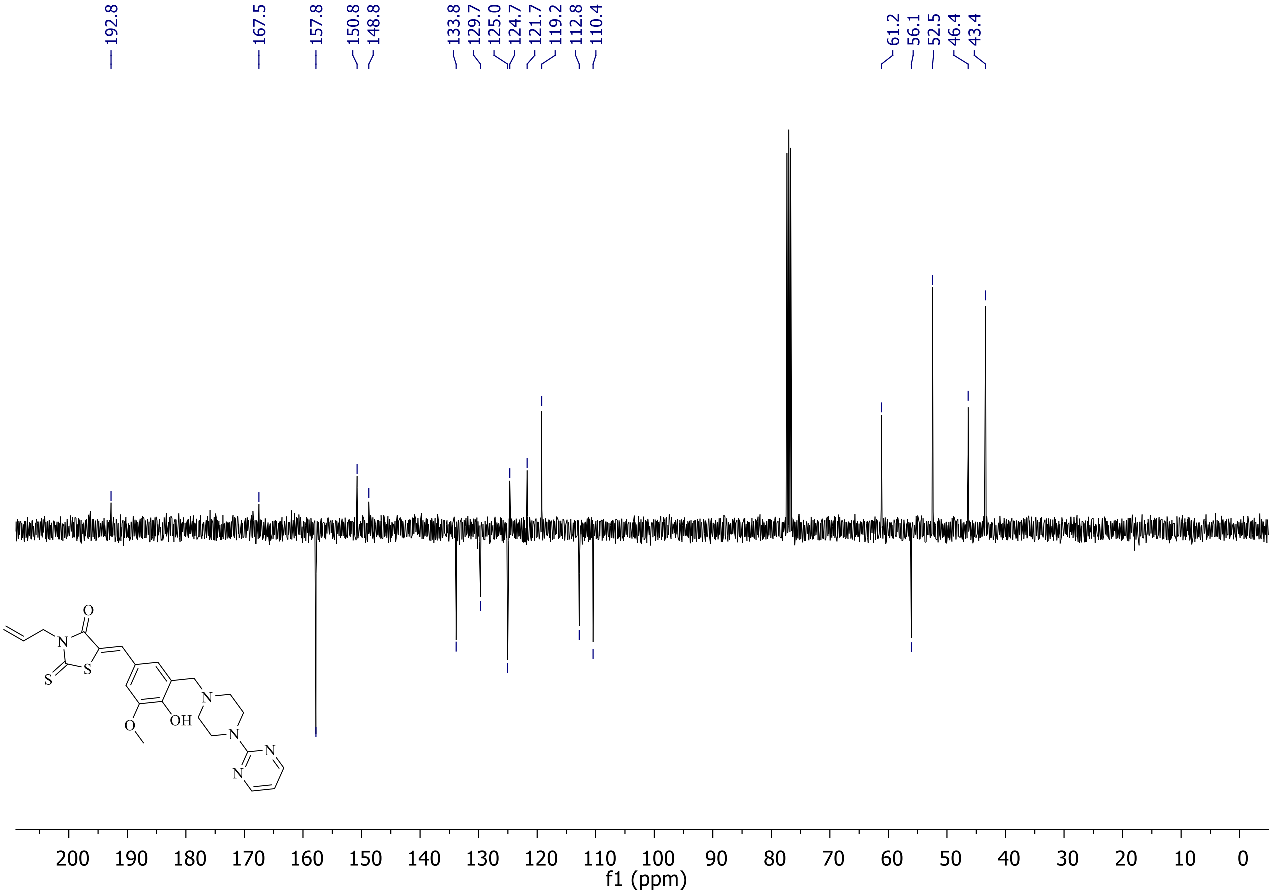

Figure S26. ^{13}C NMR Spectrum of Compound 7

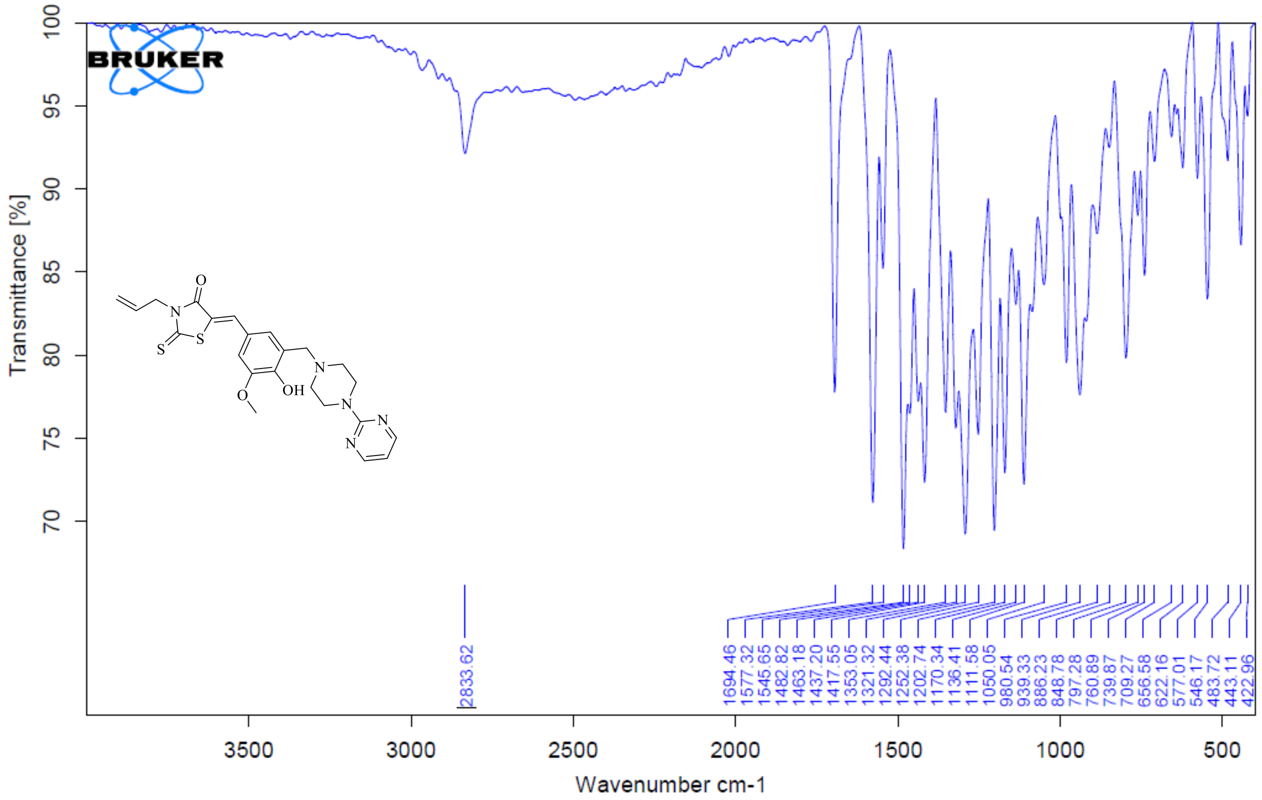

Figure S27. FTIR Spectrum of Compound 7

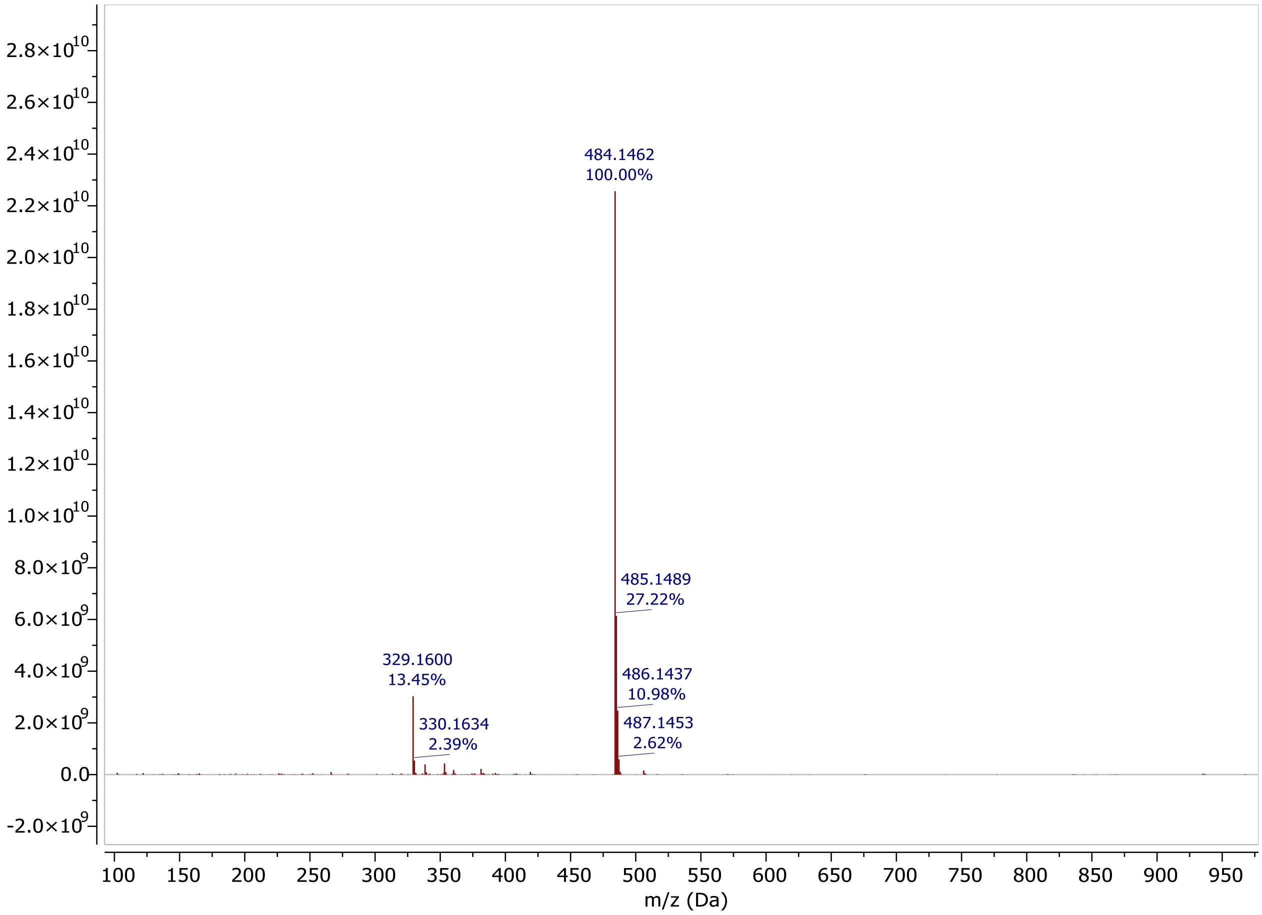

Figure S28. HRMS Spectrum of Compound 7

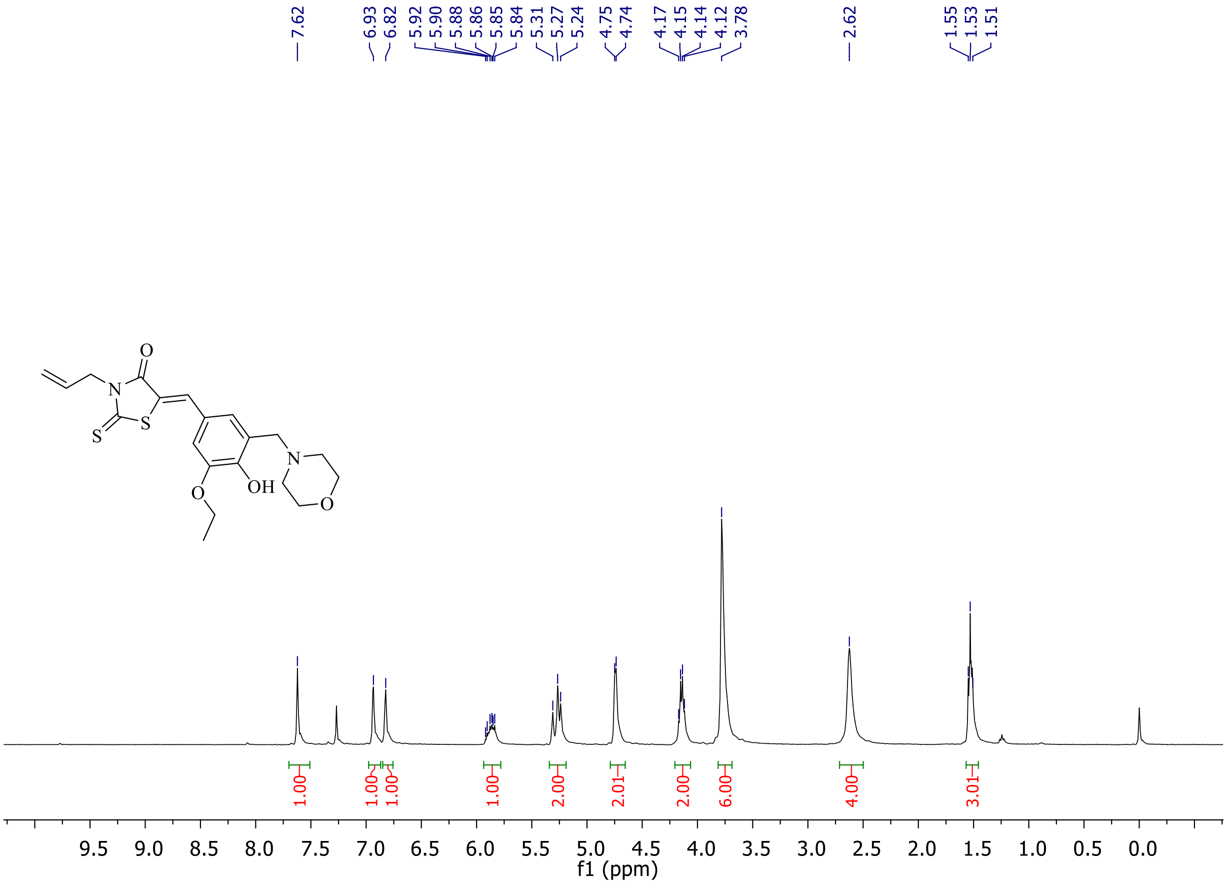

Figure S29. ^{1}H NMR Spectrum of Compound 8

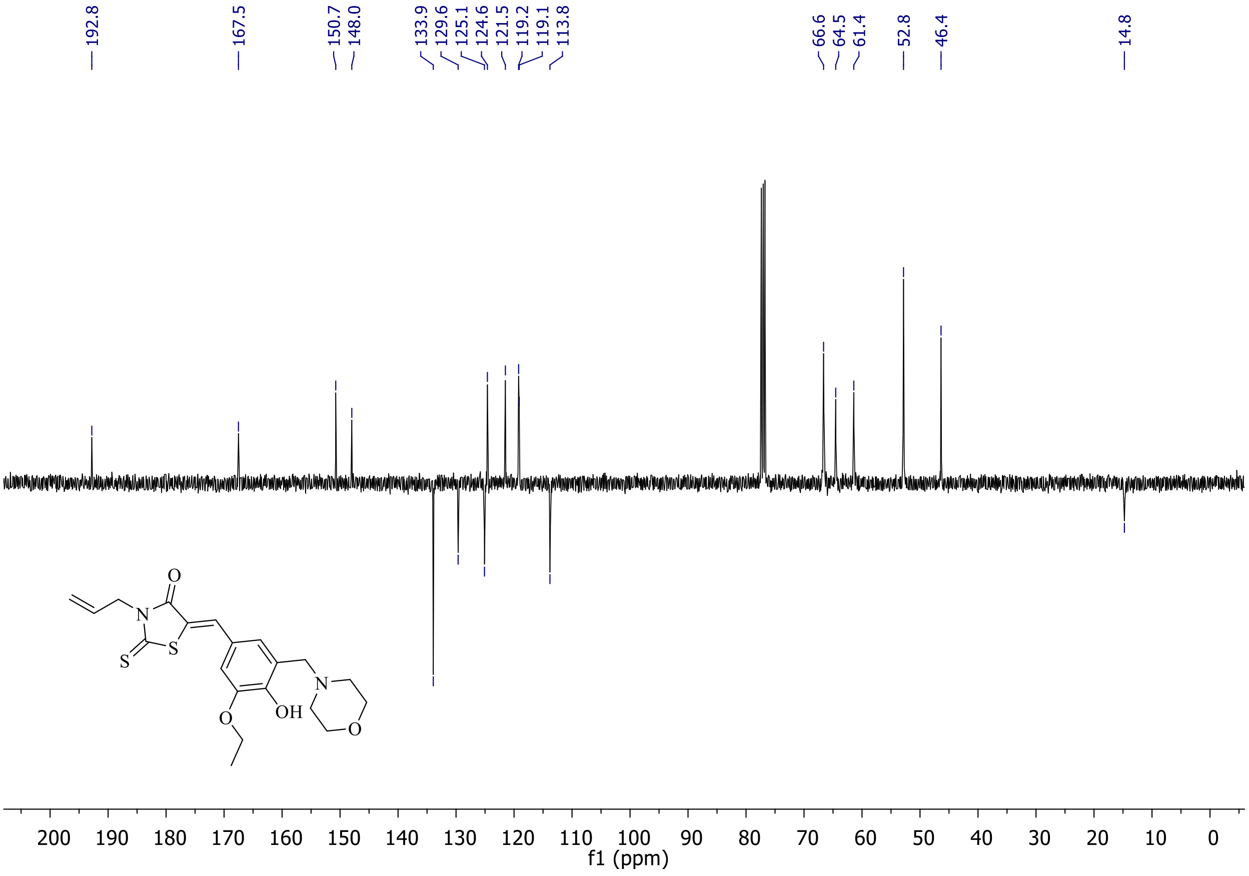

Figure S30. ^{13}C NMR Spectrum of Compound 8

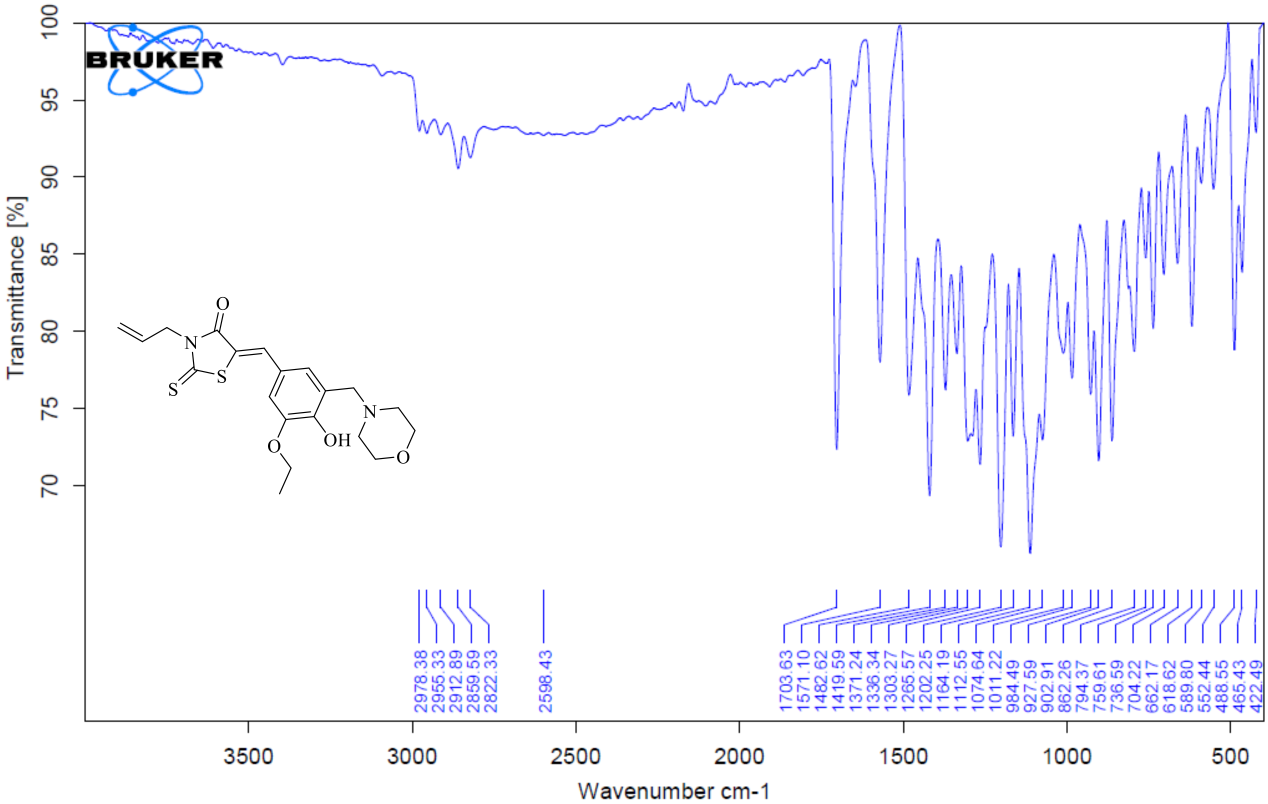

Figure S31. FTIR Spectrum of Compound 8

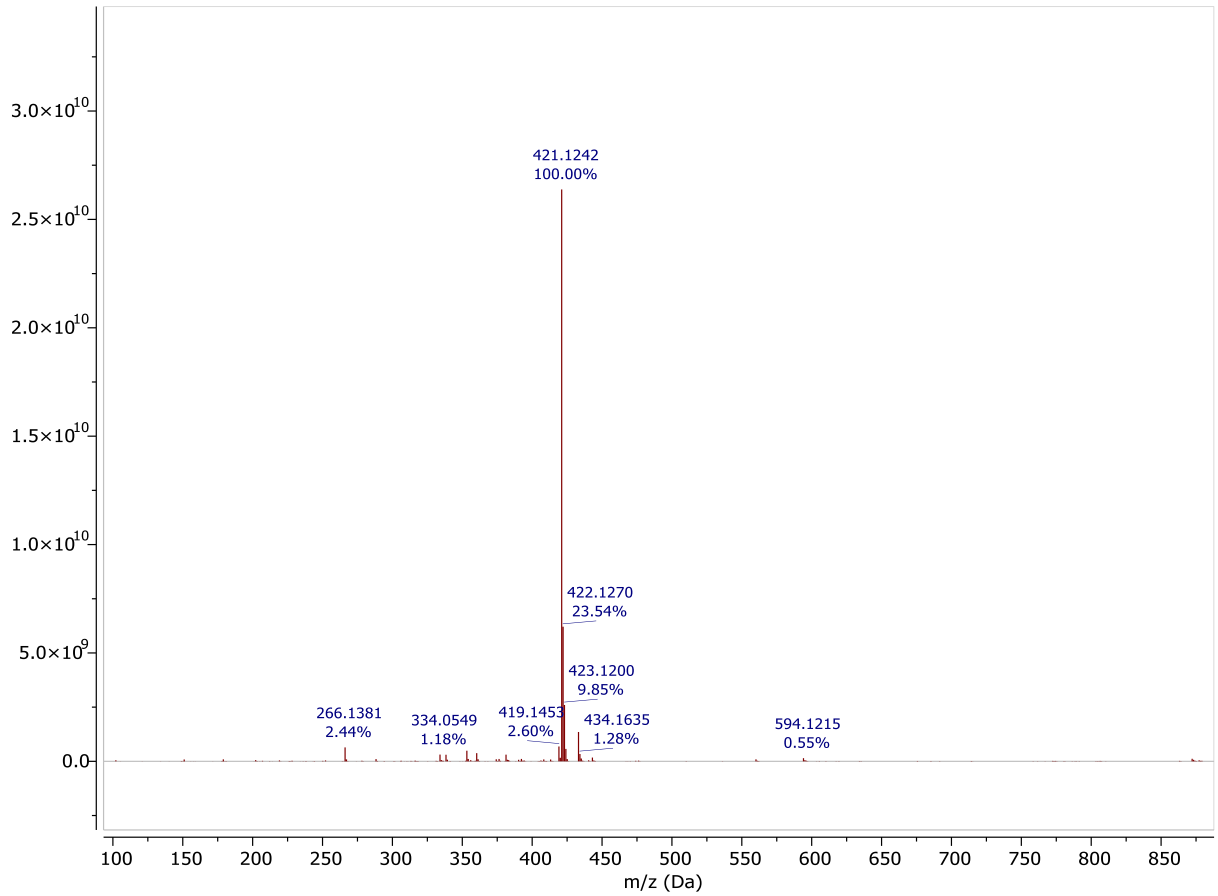

Figure S32. HRMS Spectrum of Compound 8

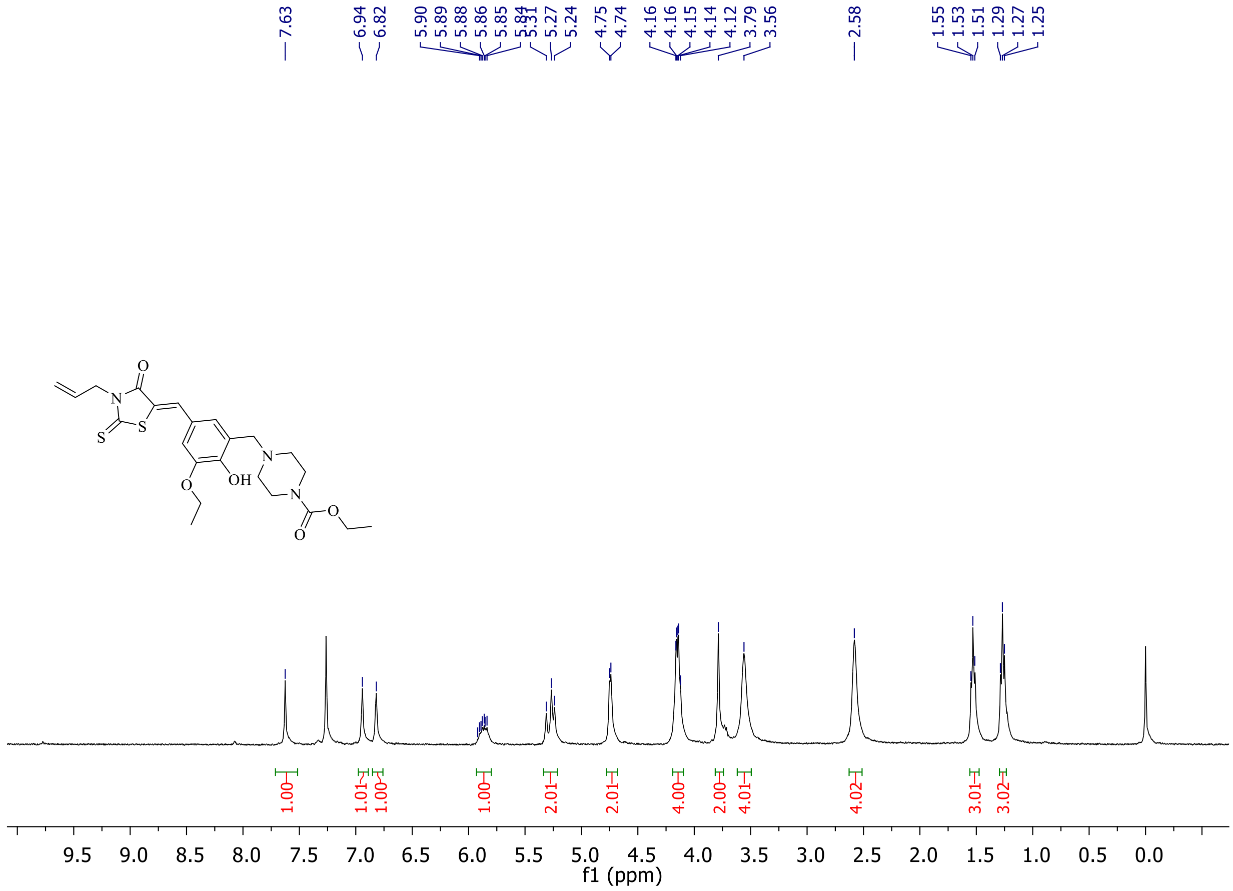

Figure S33. ^{1}H NMR Spectrum of Compound 9

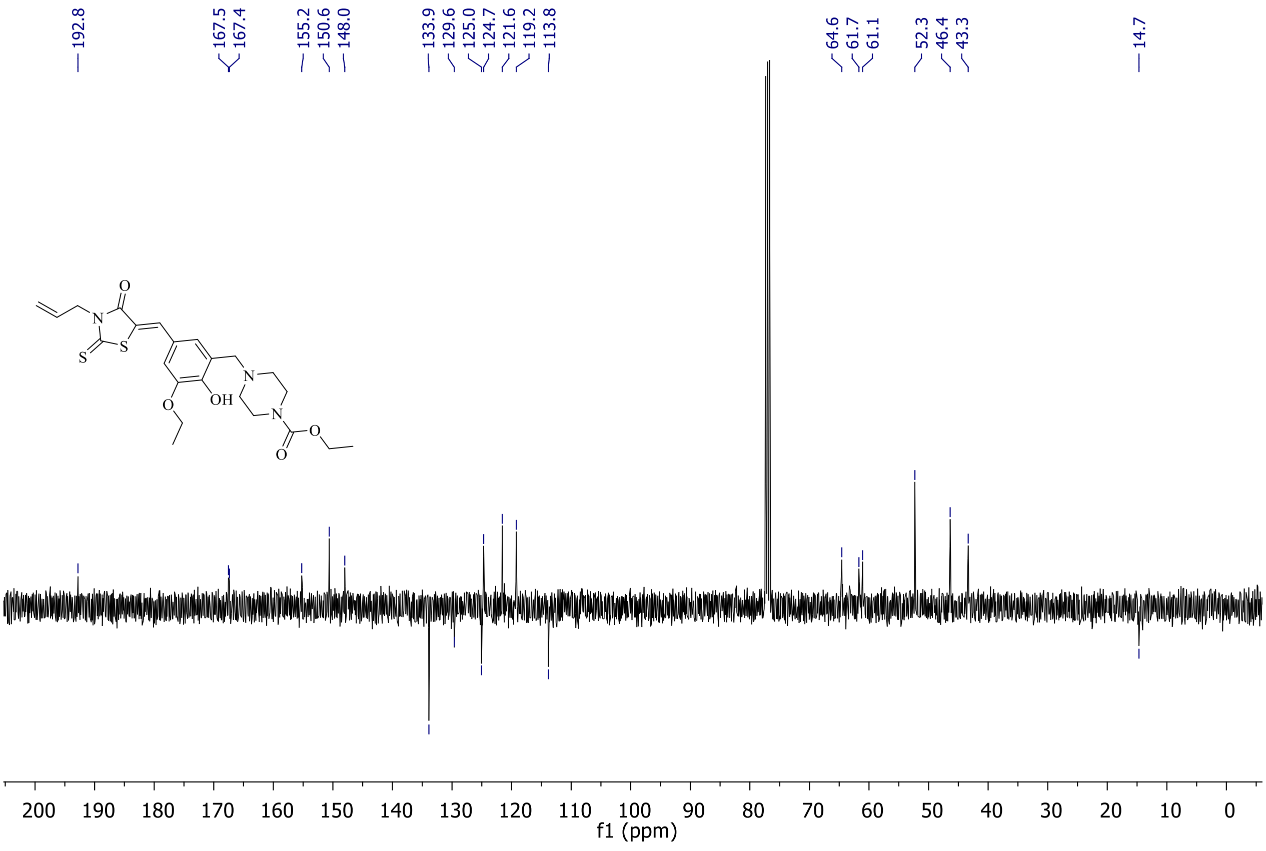

Figure S34. ^{13}C NMR Spectrum of Compound 9

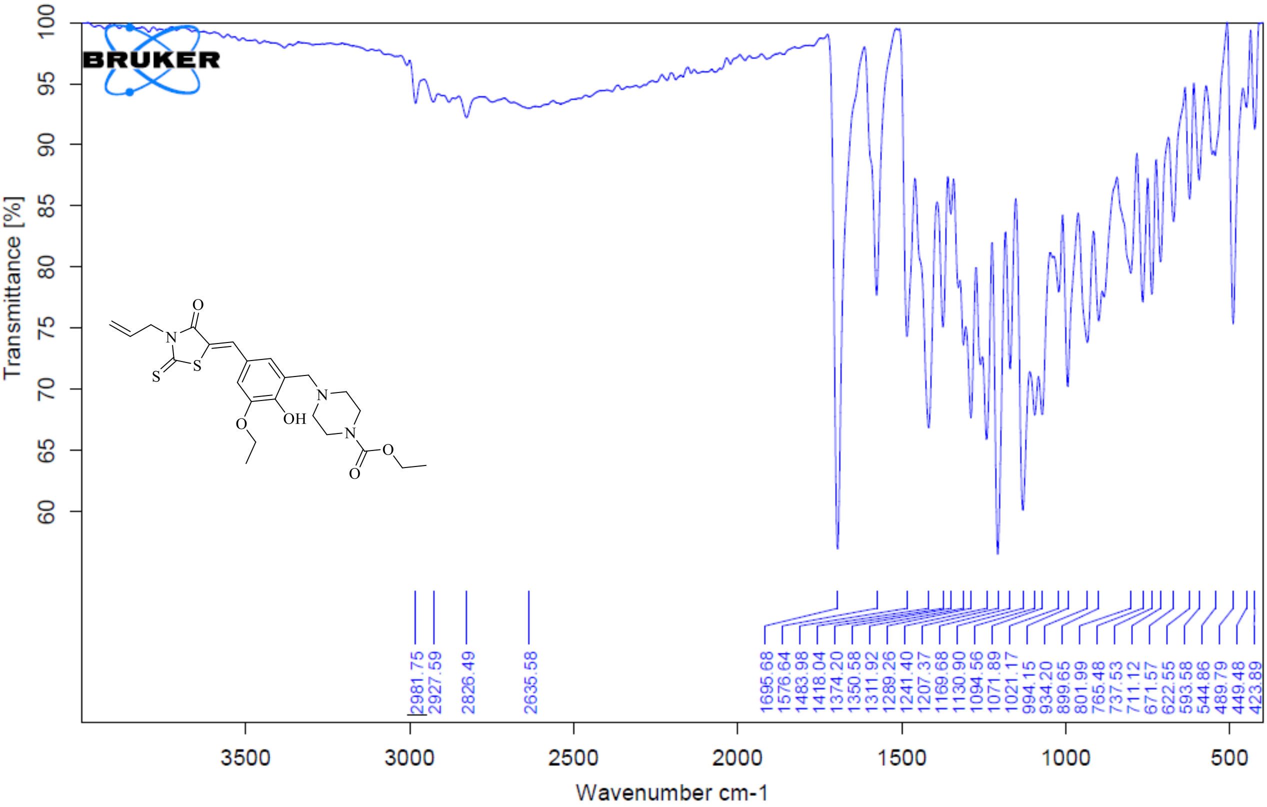

Figure S35. FTIR Spectrum of Compound 9

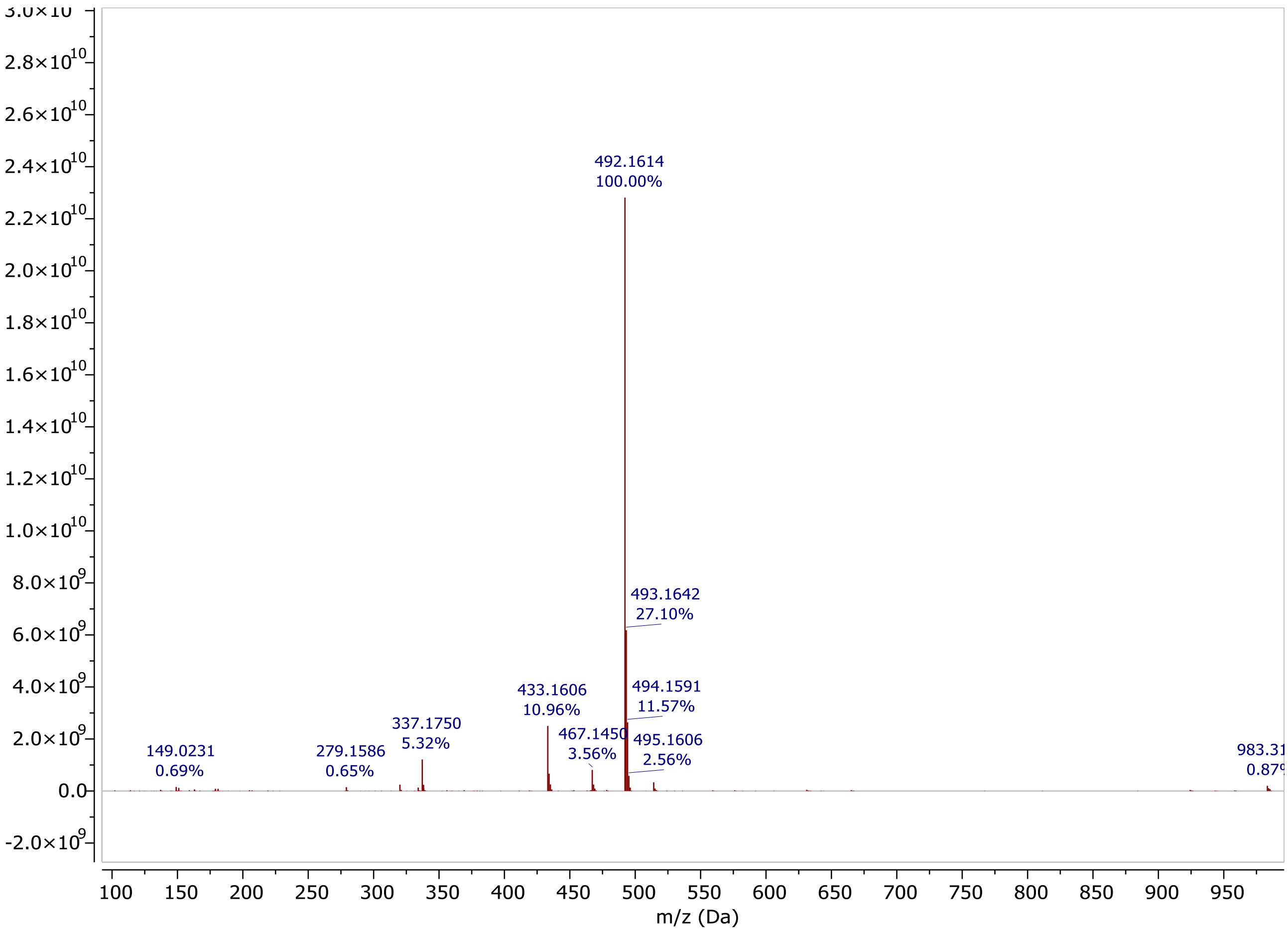

Figure S36. HRMS Spectrum of Compound 9

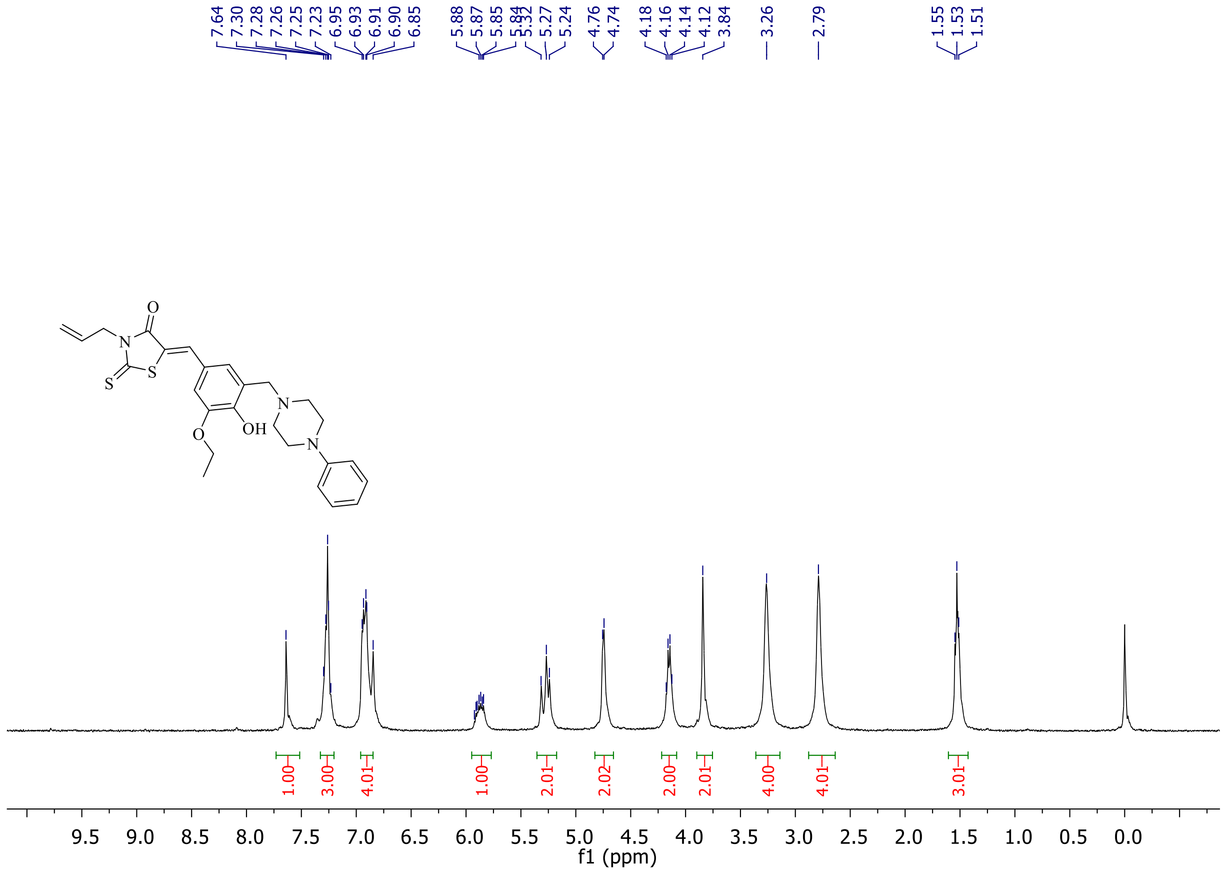

Figure S37. ^{1}H NMR Spectrum of Compound 10

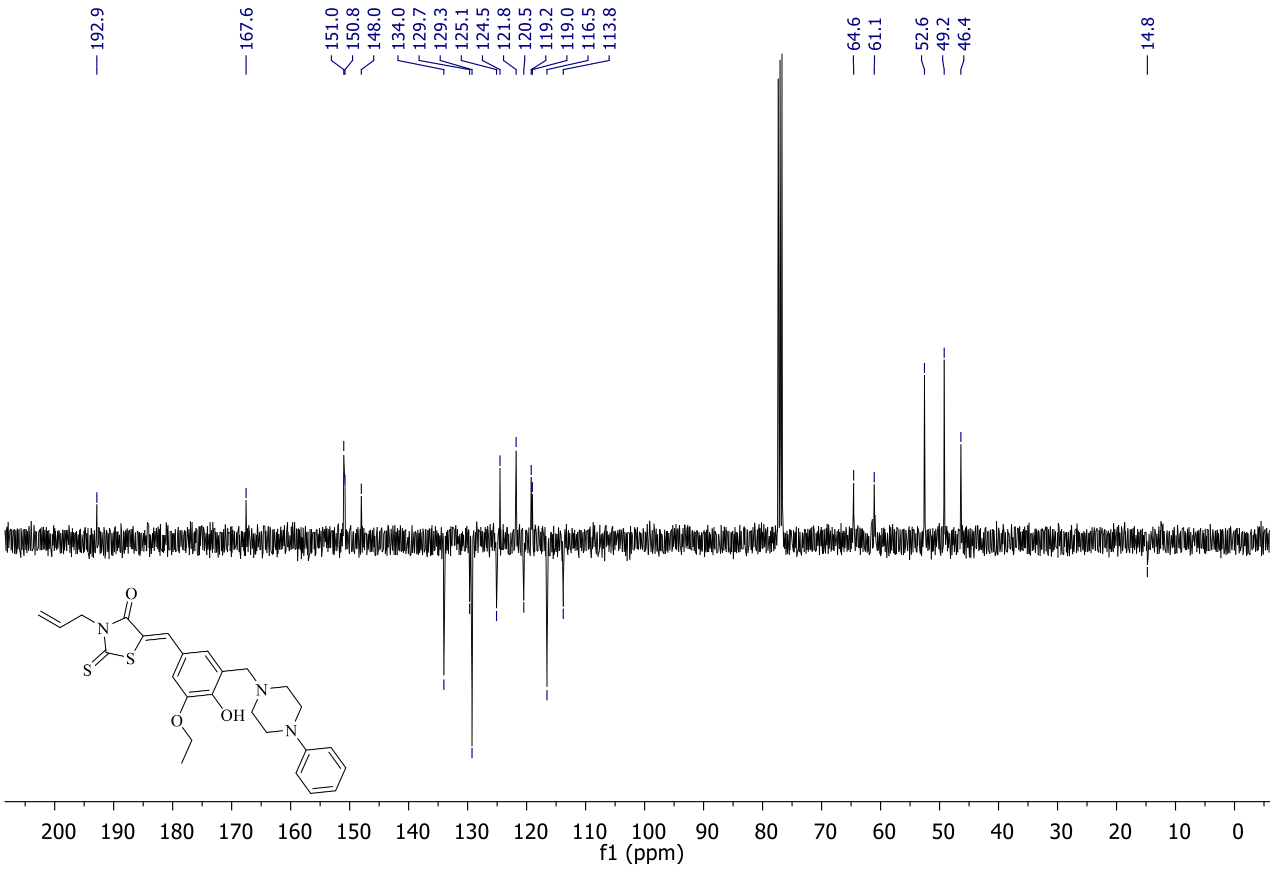

Figure S38. ^{13}C NMR Spectrum of Compound 10

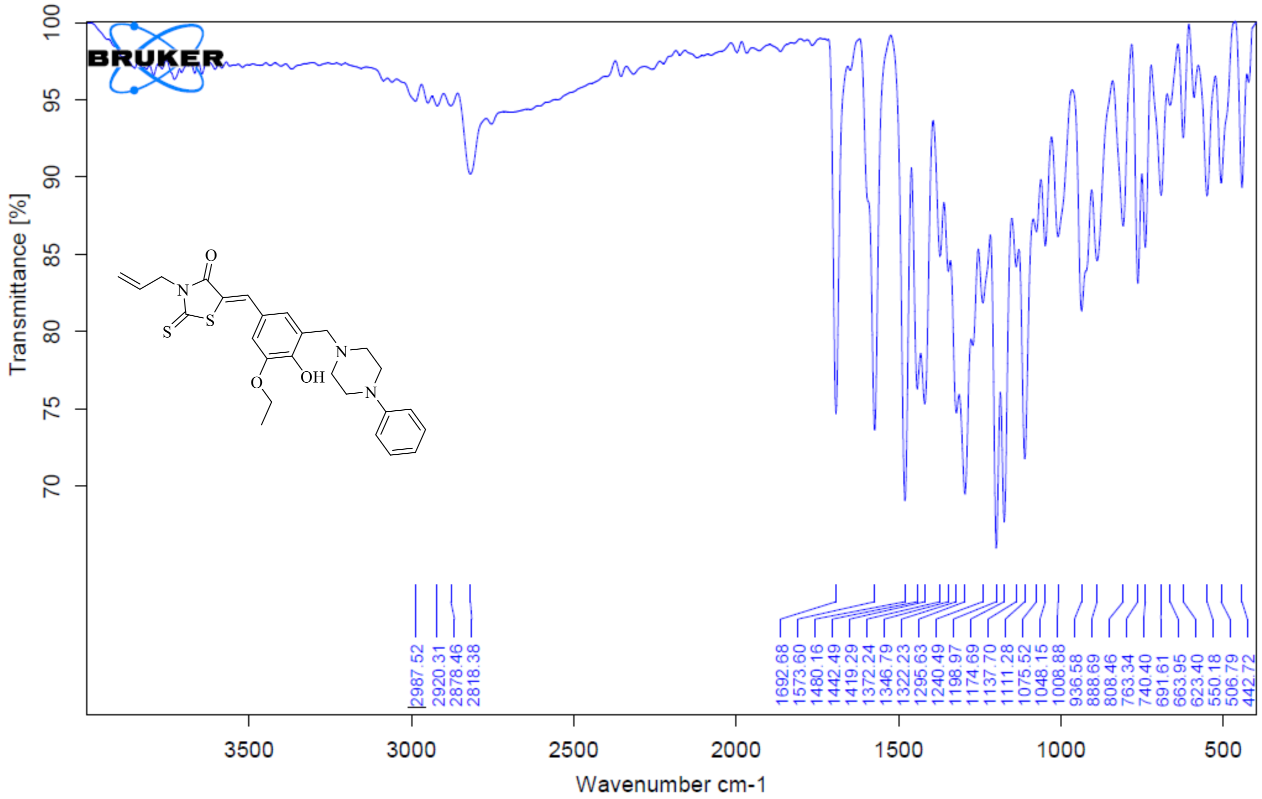

Figure S39. FTIR Spectrum of Compound 10

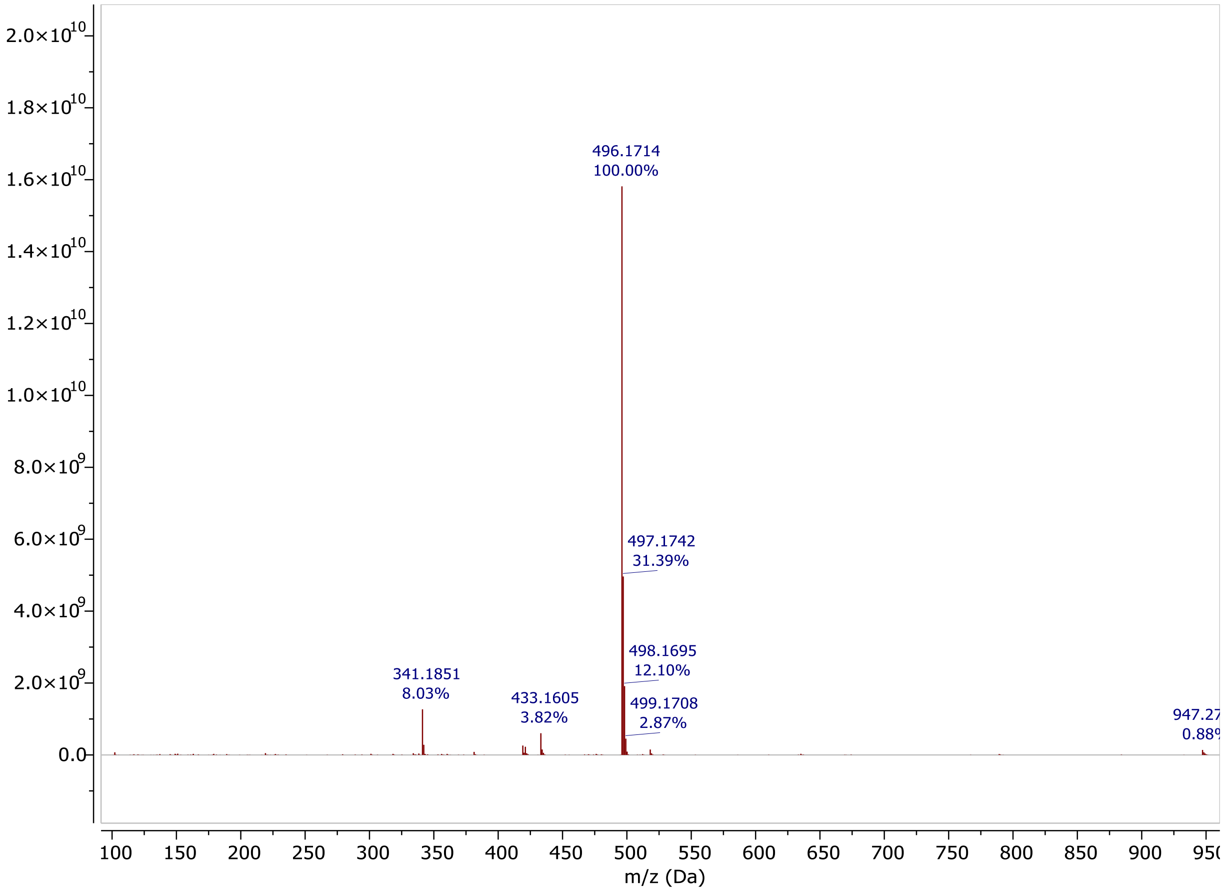

Figure S40. HRMS Spectrum of Compound 10

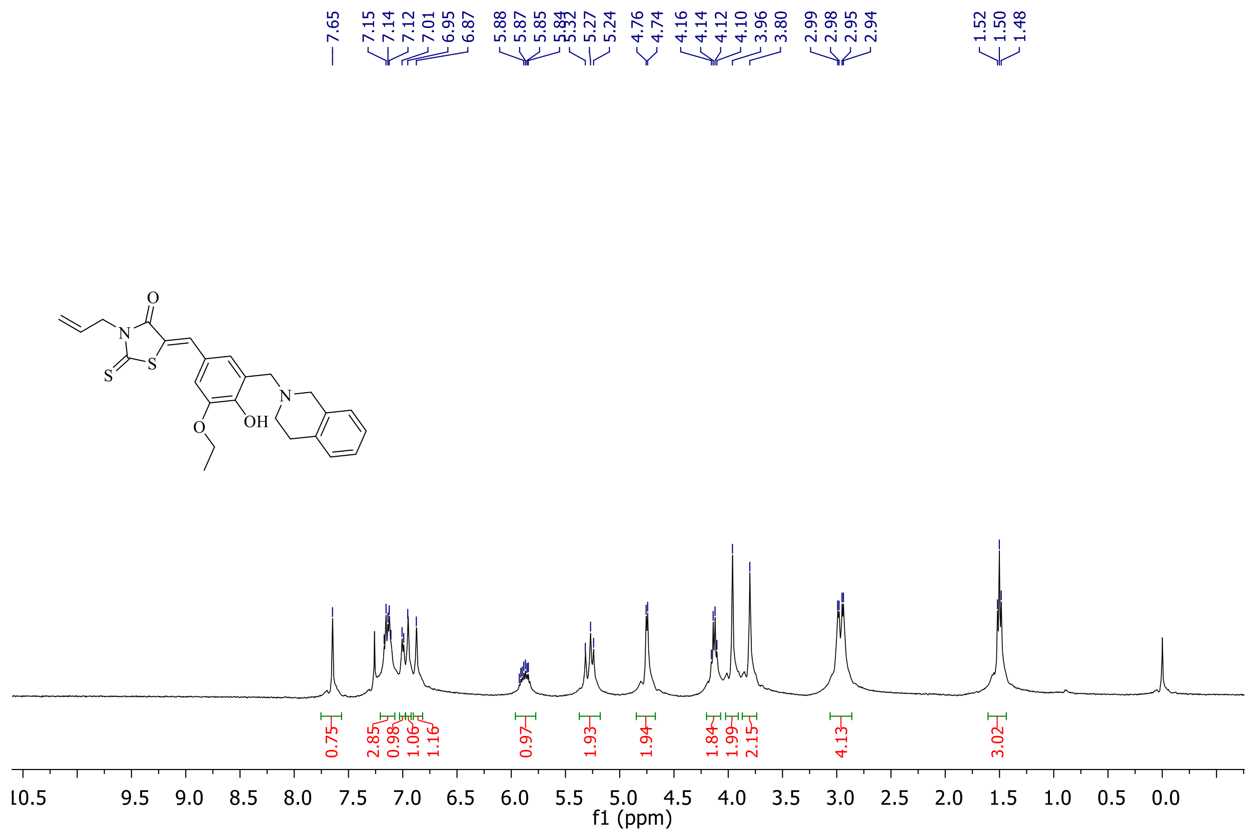

Figure S41. ^{1}H NMR Spectrum of Compound 11

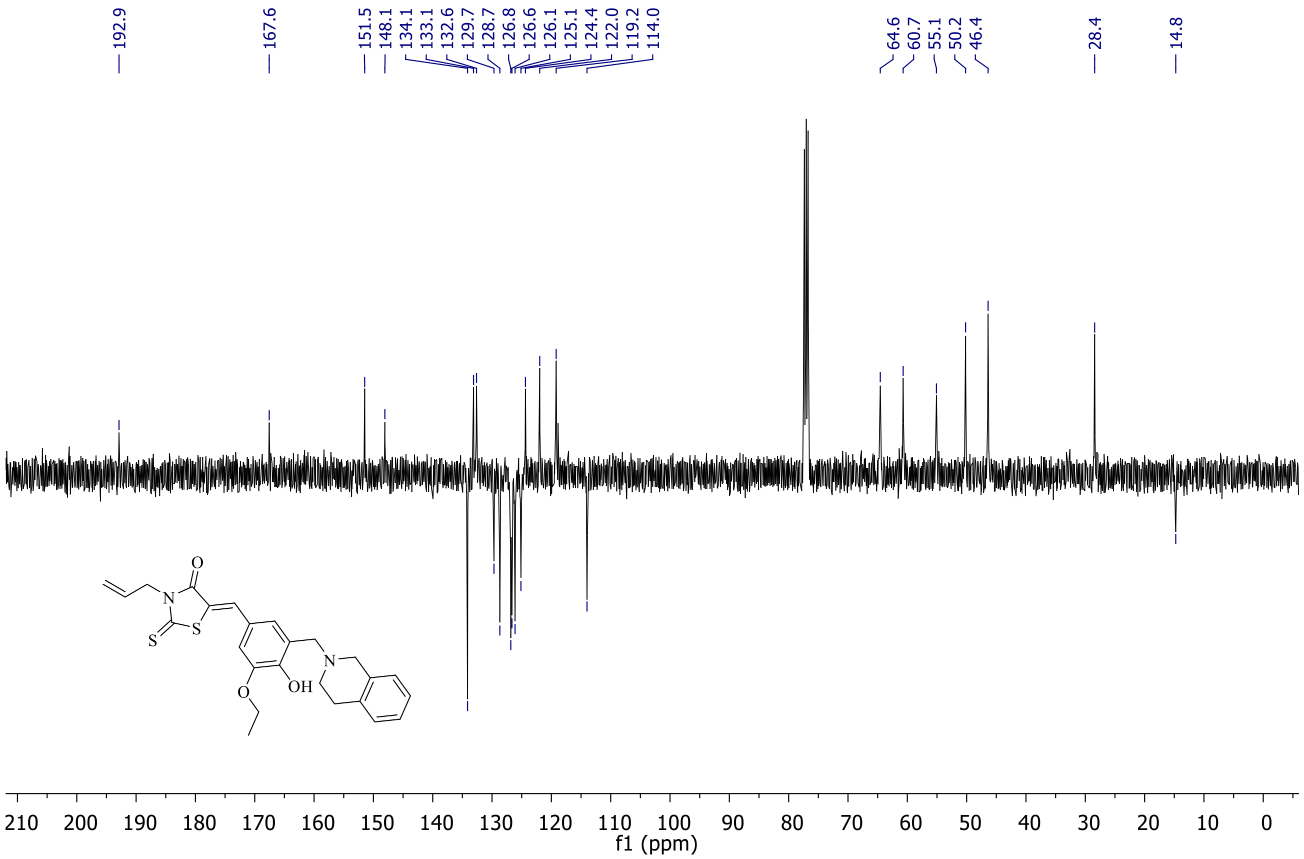

Figure S42. ^{13}C NMR Spectrum of Compound 11

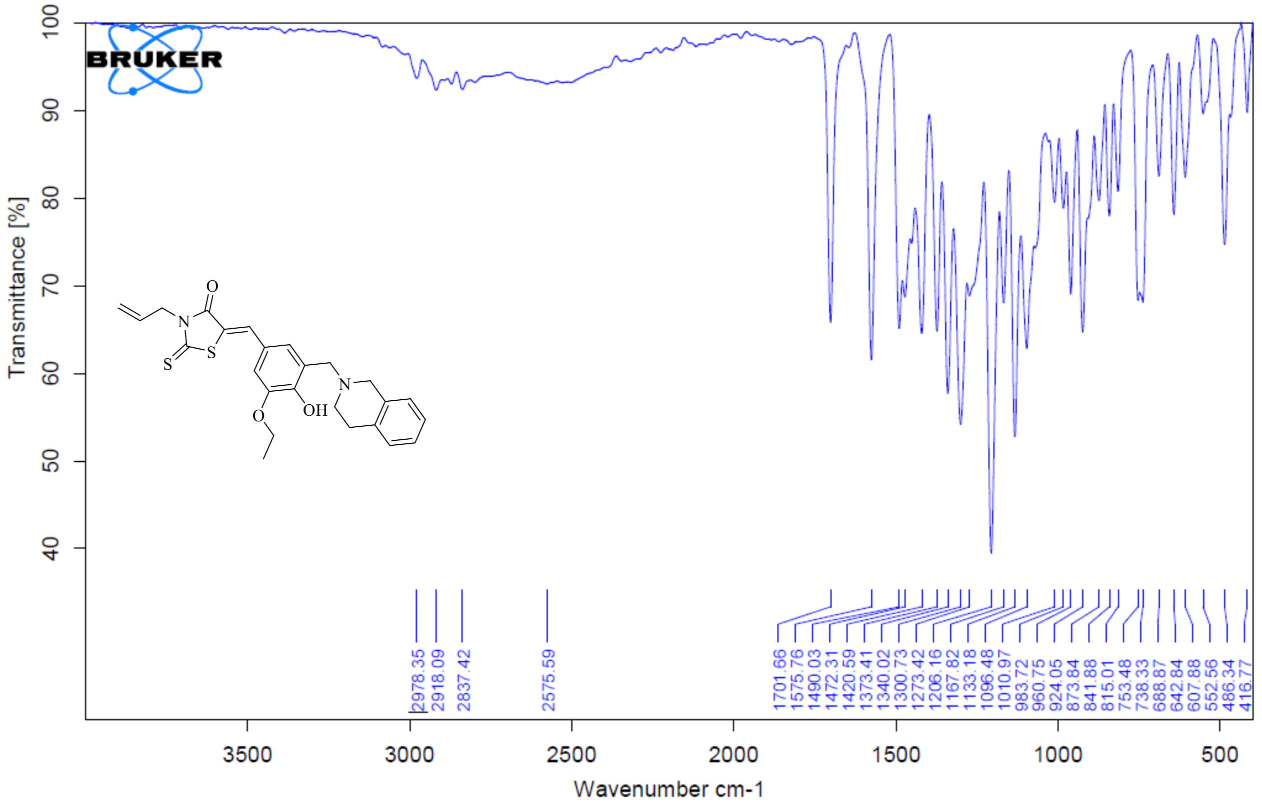

Figure S43. FTIR Spectrum of Compound 11

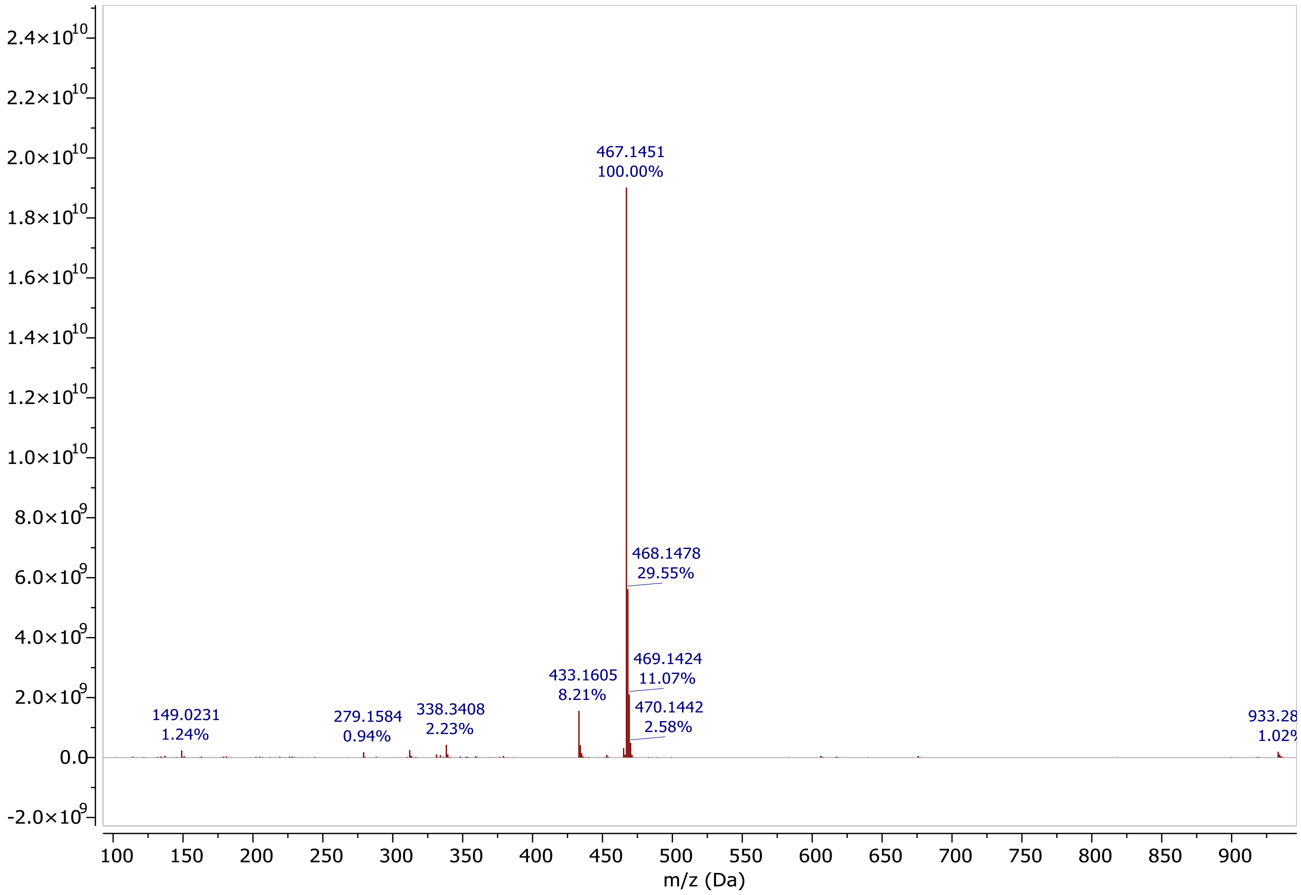

Figure S44. HRMS Spectrum of Compound 11

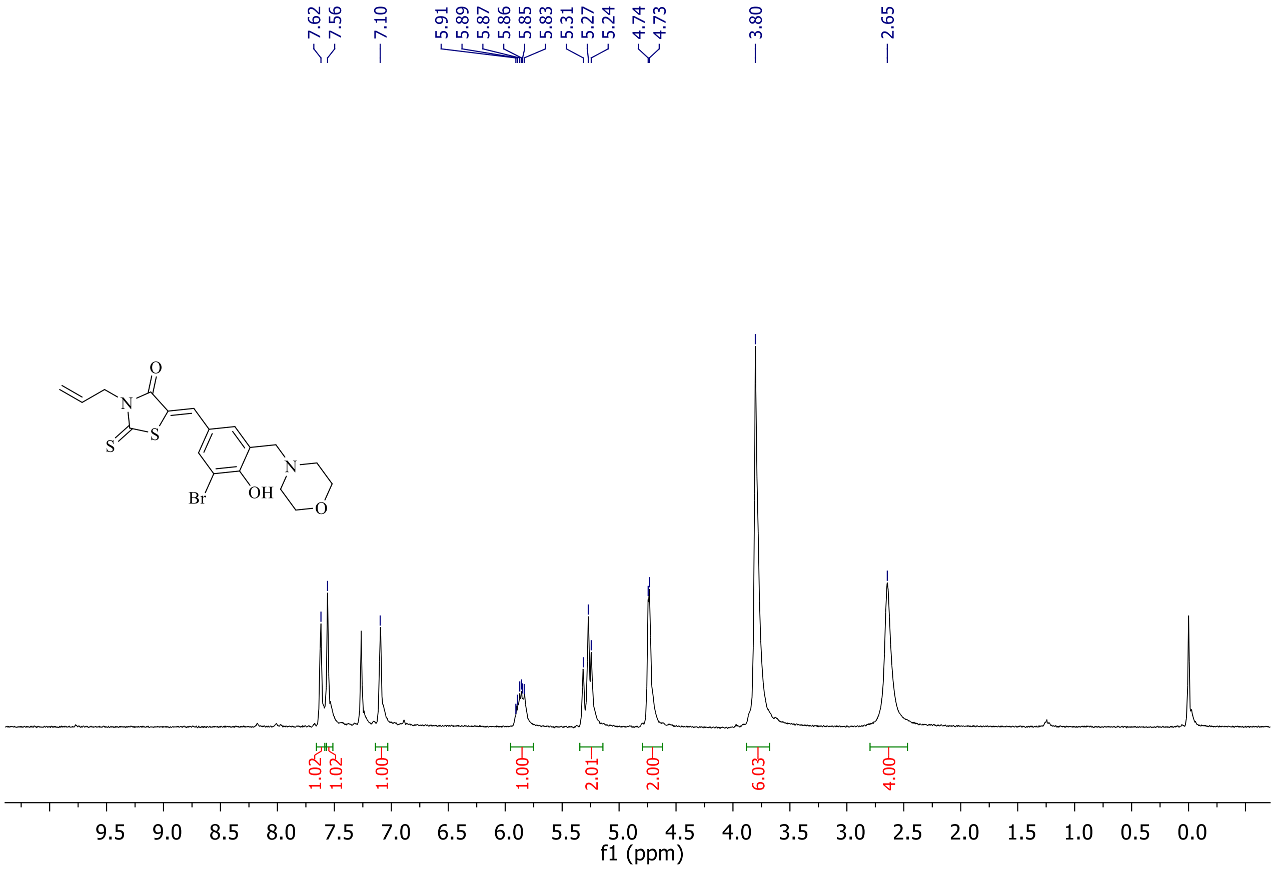

Figure S45. ^{1}H NMR Spectrum of Compound 12

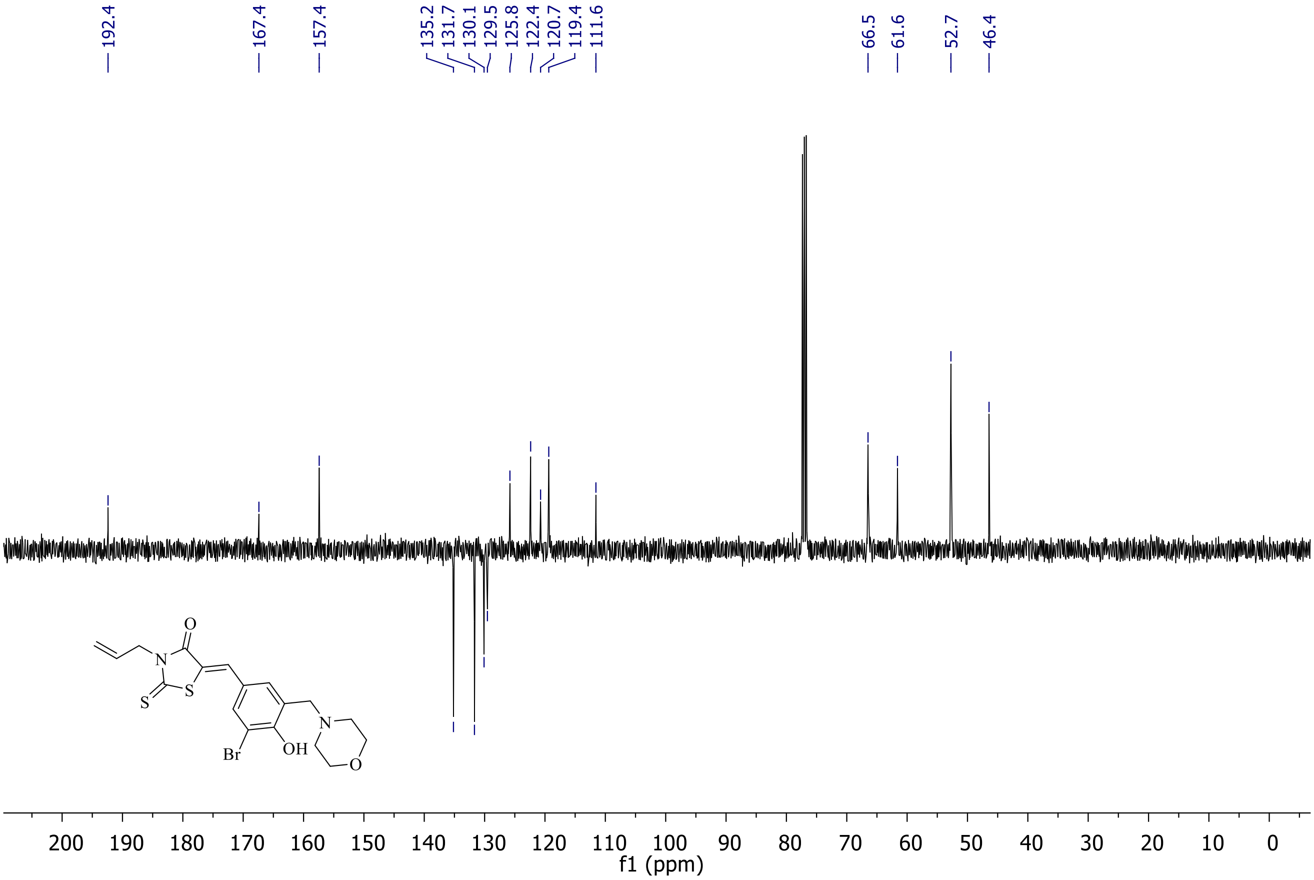

Figure S46. ^{13}C NMR Spectrum of Compound 12

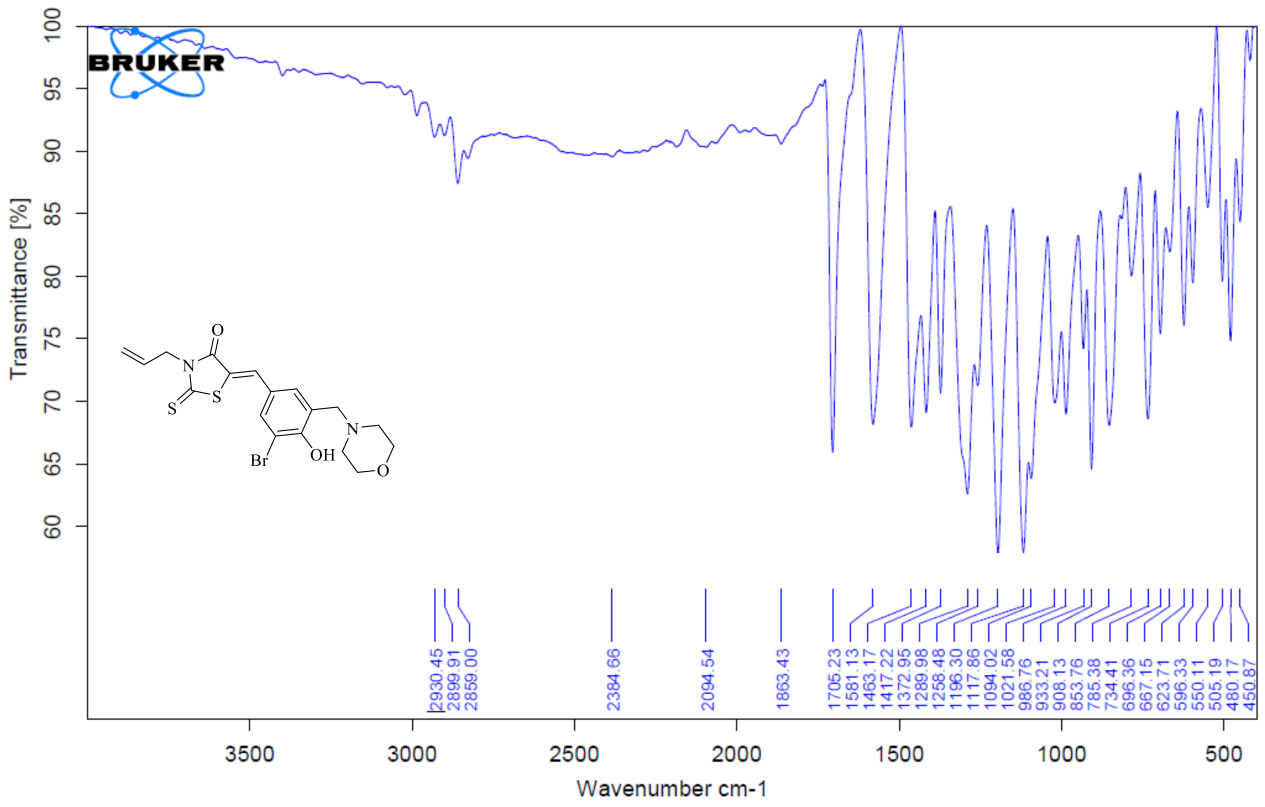

Figure S47. FTIR Spectrum of Compound 12

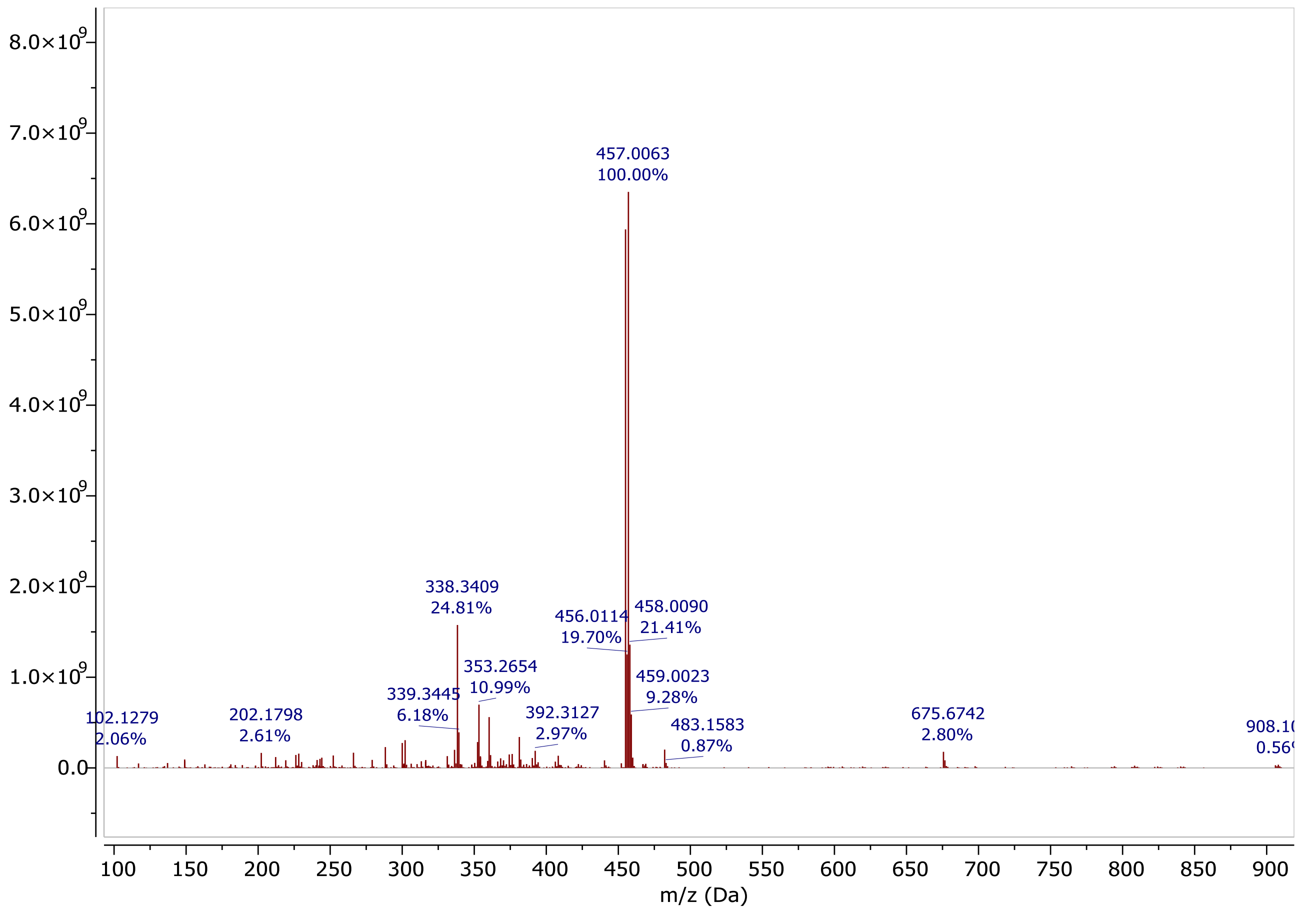

Figure S48. HRMS Spectrum of Compound 12

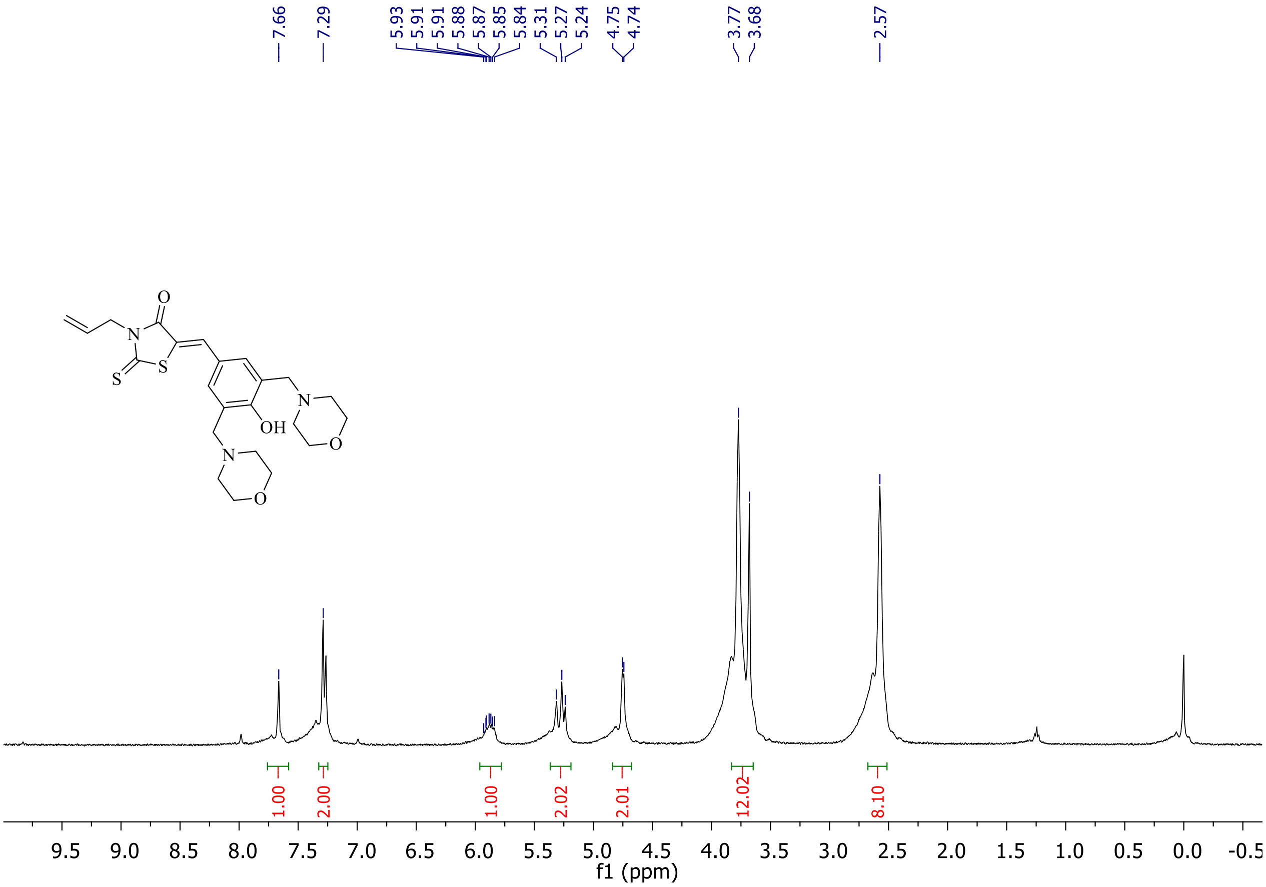

Figure S49. ^{1}H NMR Spectrum of Compound 13

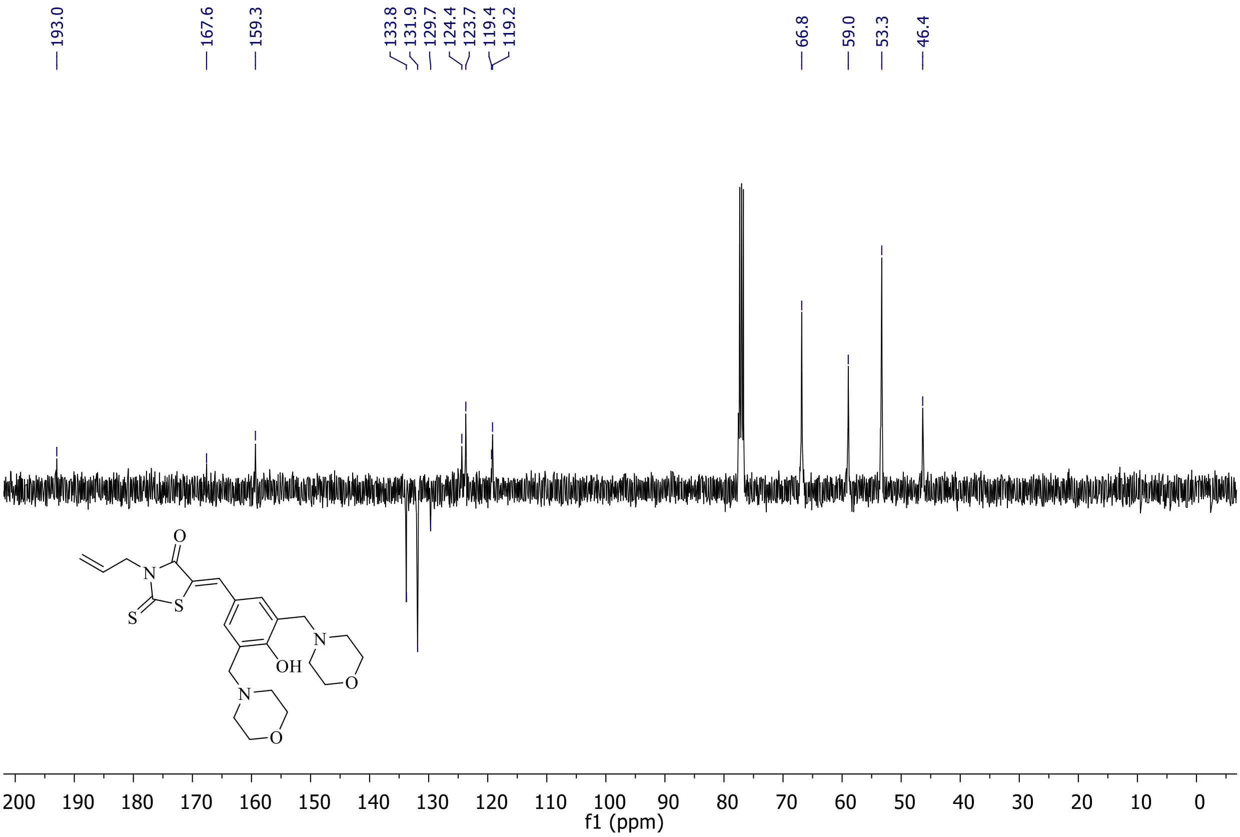

Figure S50. ^{13}C NMR Spectrum of Compound 13

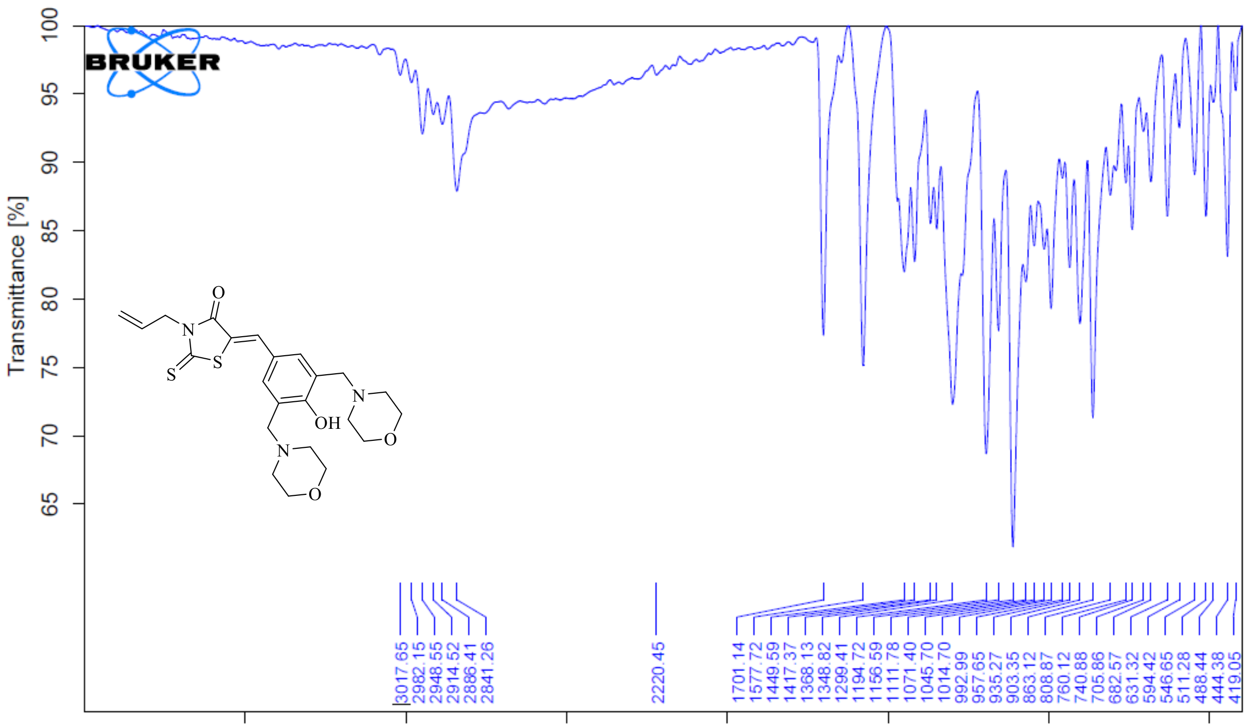

Figure S51. FTIR Spectrum of Compound 13

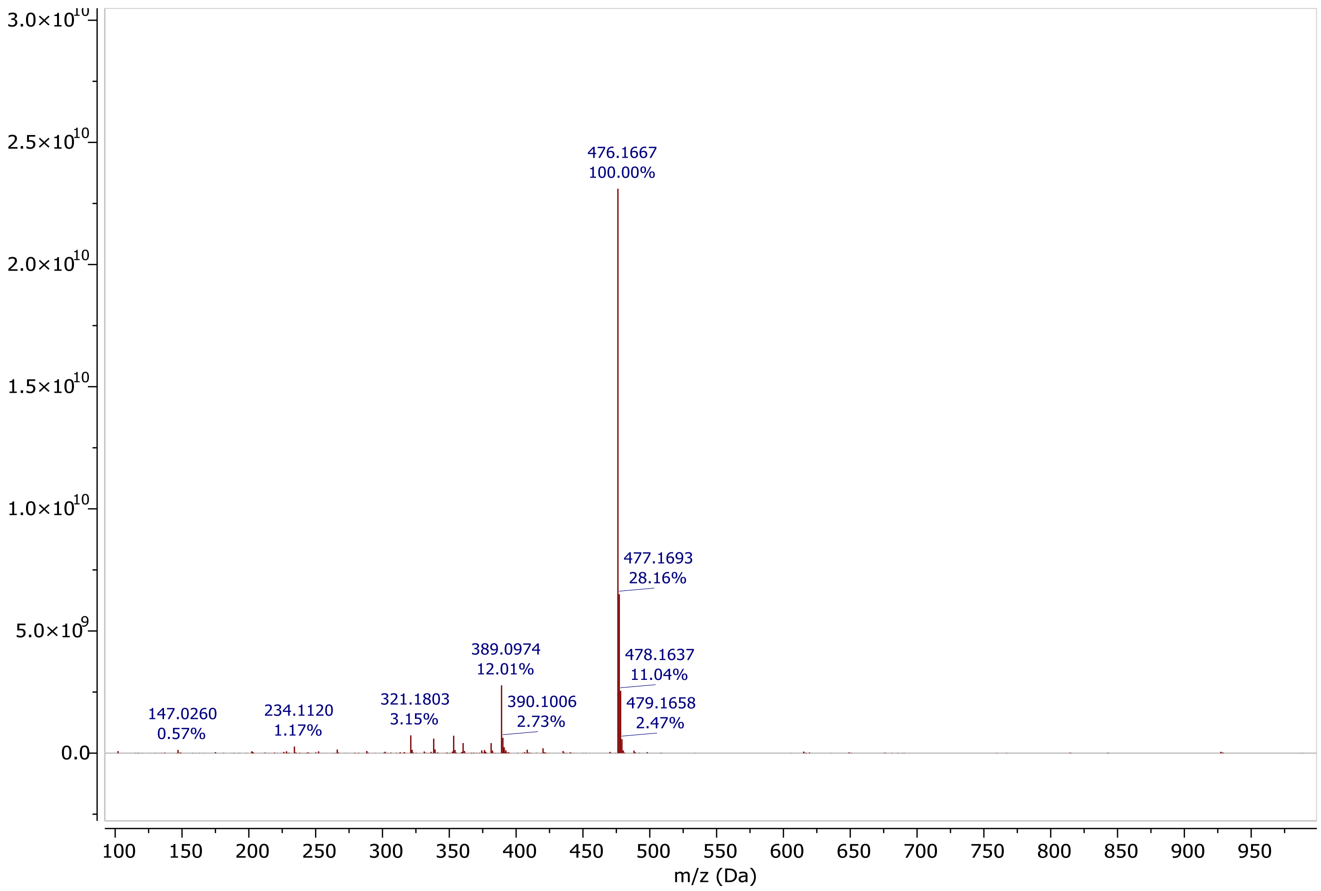

Figure S52. HRMS Spectrum of Compound 13

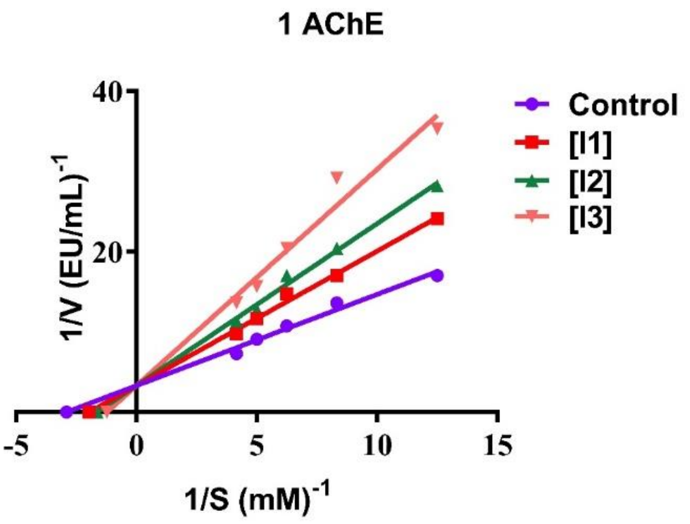

Figure S53. Lineweaver-Burk plots of compound 1 for AChE

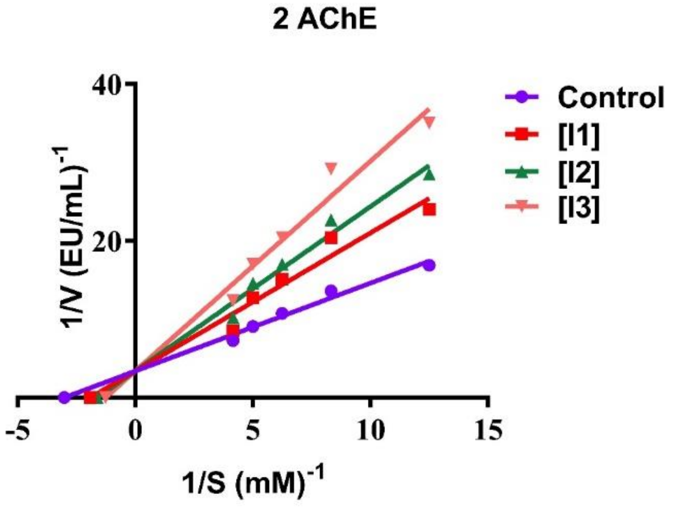

Figure S54. Lineweaver-Burk plots of compound 2 for AChE

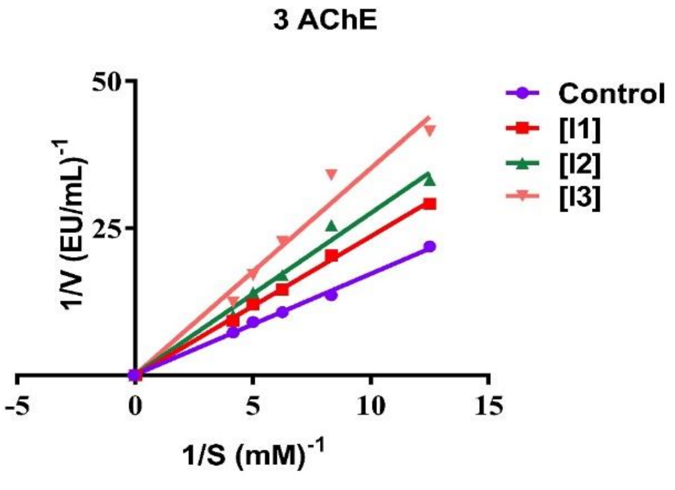

Figure S55. Lineweaver-Burk plots of compound 3 for AChE

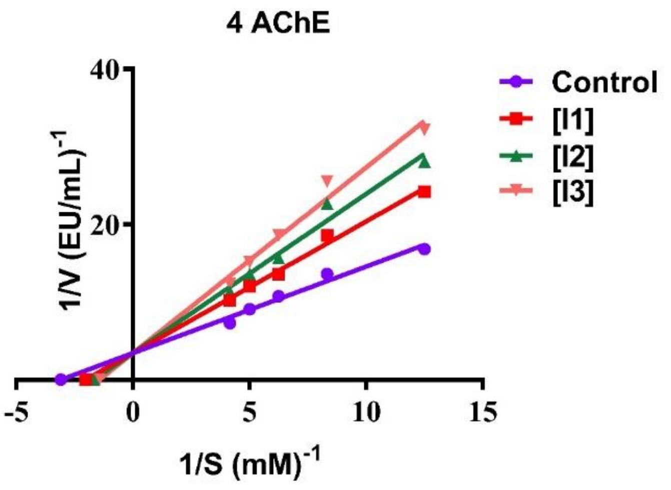

Figure S56. Lineweaver-Burk plots of compound 4 for AChE

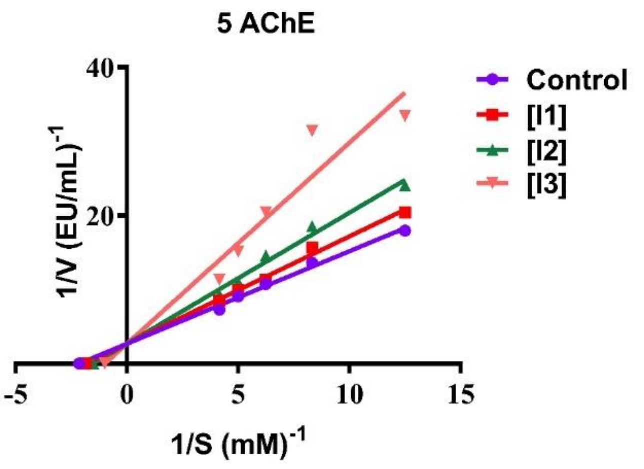

Figure S57. Lineweaver-Burk plots of compound 5 for AChE

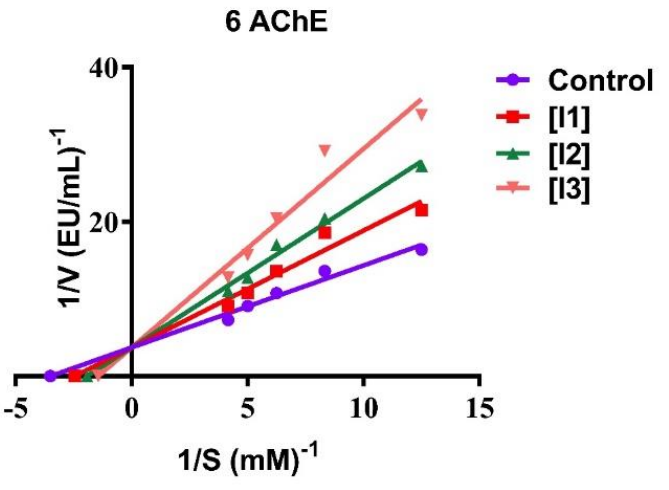

Figure S58. Lineweaver-Burk plots of compound 6 for AChE

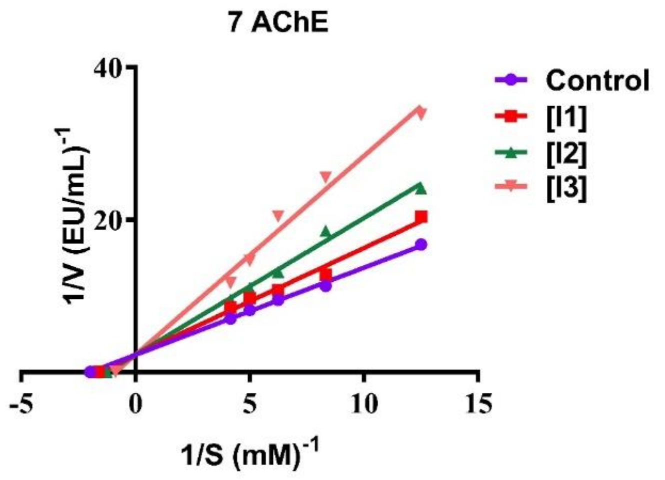

Figure S59. Lineweaver-Burk plots of compound 7 for AChE

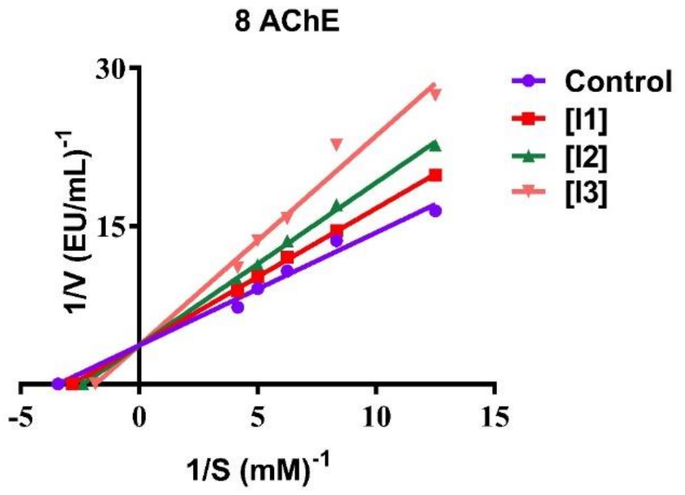

Figure S60. Lineweaver-Burk plots of compound 8 for AChE

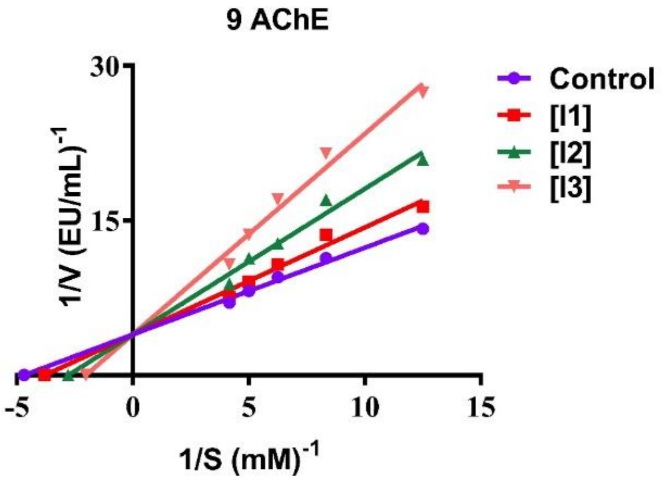

Figure S61. Lineweaver-Burk plots of compound 9 for AChE

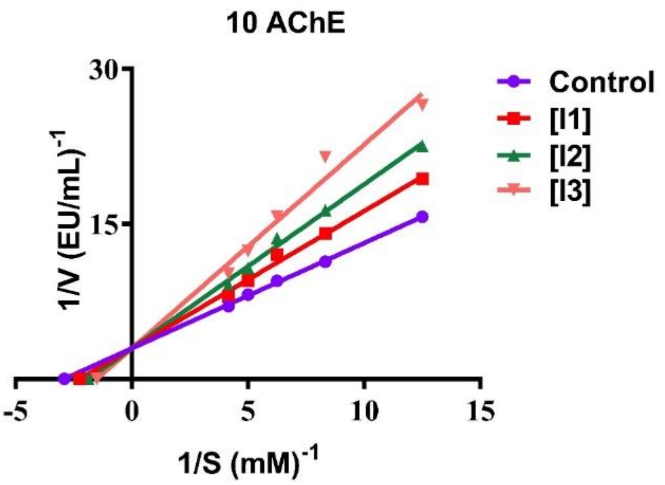

Figure S62. Lineweaver-Burk plots of compound 10 for AChE

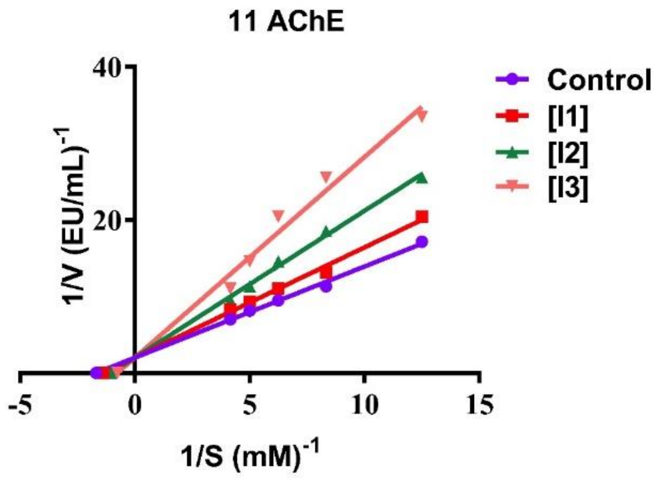

Figure S63. Lineweaver-Burk plots of compound 11 for AChE

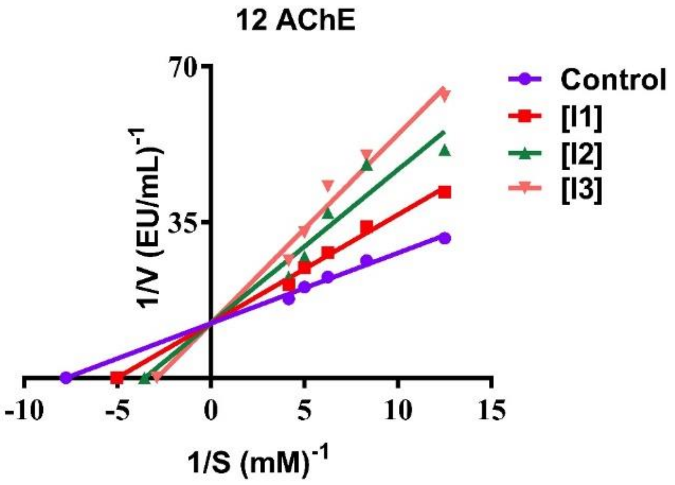

Figure S64. Lineweaver-Burk plots of compound 12 for AChE

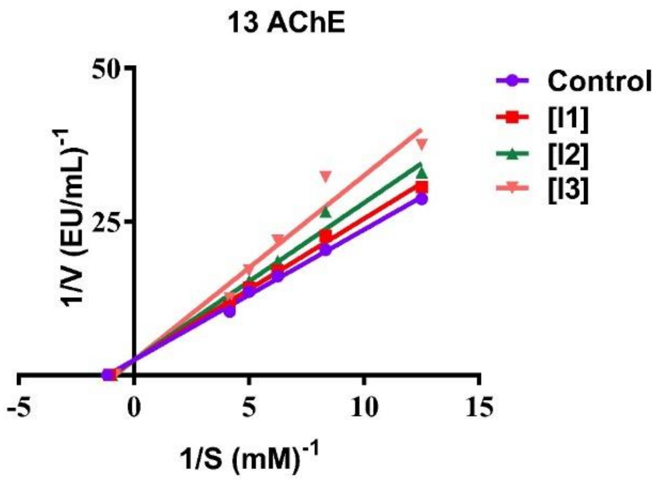

Figure S65. Lineweaver-Burk plots of compound 13 for AChE

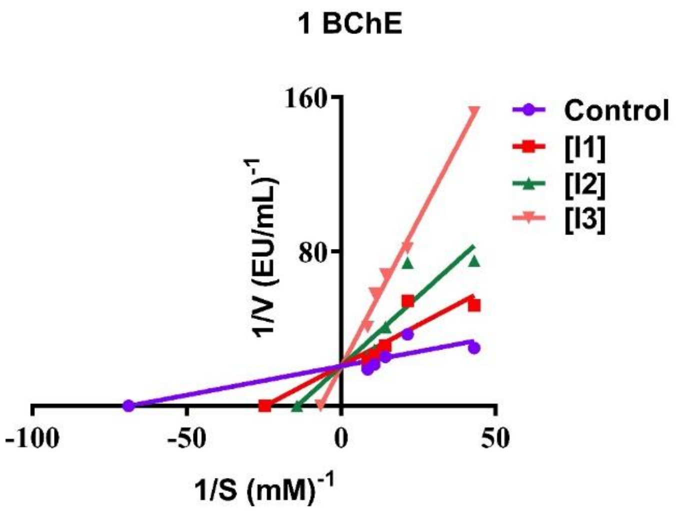

Figure S66. Lineweaver-Burk plots of compound 1 for BChE

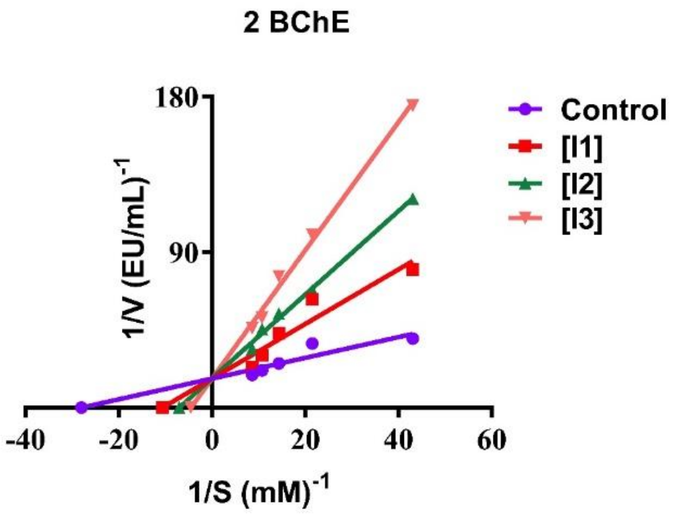

Figure S67. Lineweaver-Burk plots of compound 2 for BChE

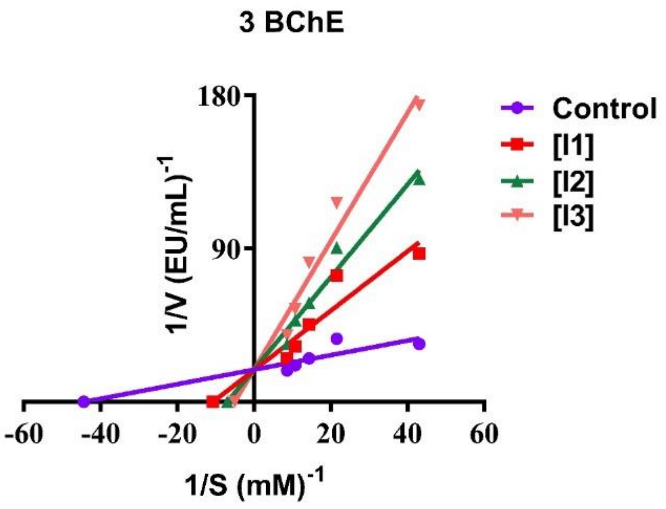

Figure S68. Lineweaver-Burk plots of compound 3 for BChE

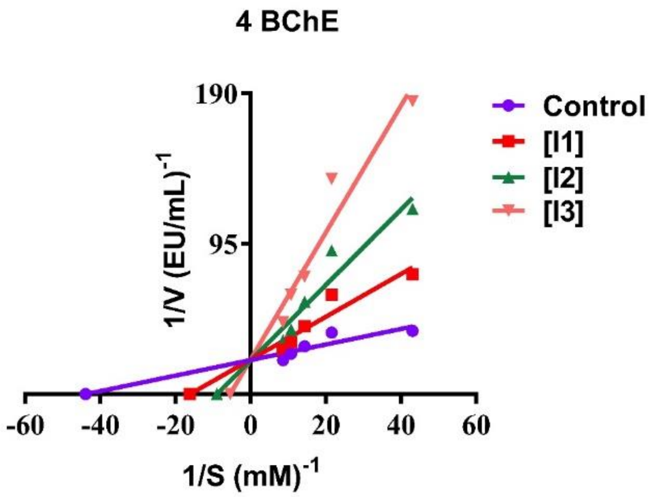

Figure S69. Lineweaver-Burk plots of compound 4 for BChE

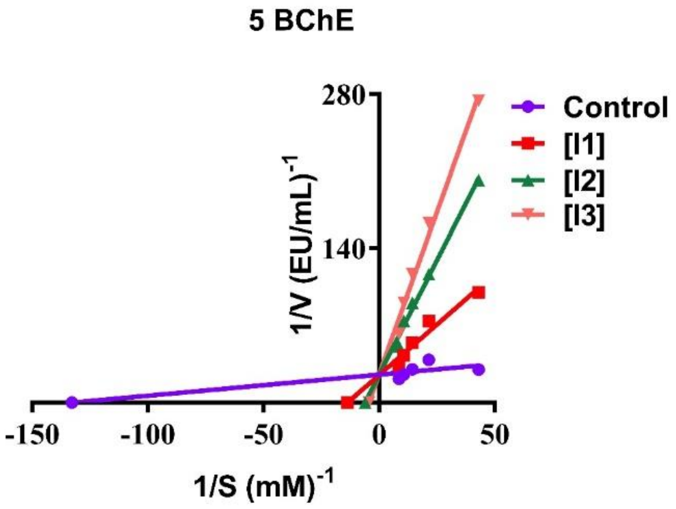

Figure S70. Lineweaver-Burk plots of compound 5 for BChE

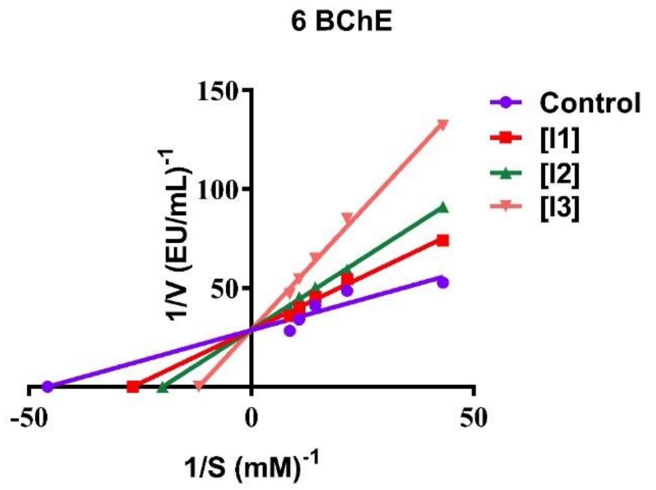

Figure S71. Lineweaver-Burk plots of compound 6 for BChE

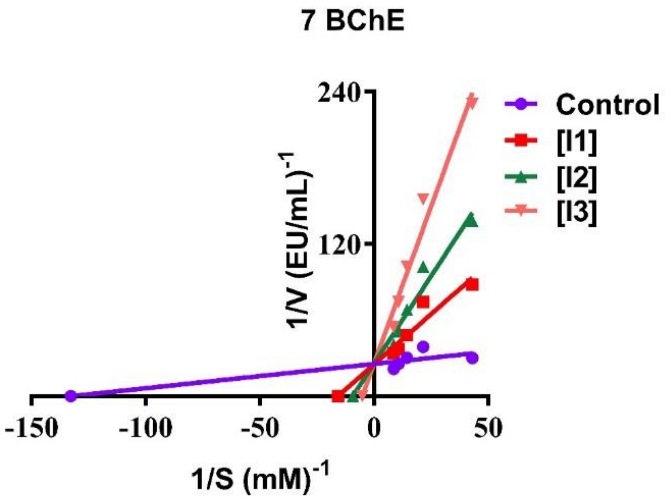

Figure S72. Lineweaver-Burk plots of compound 7 for BChE

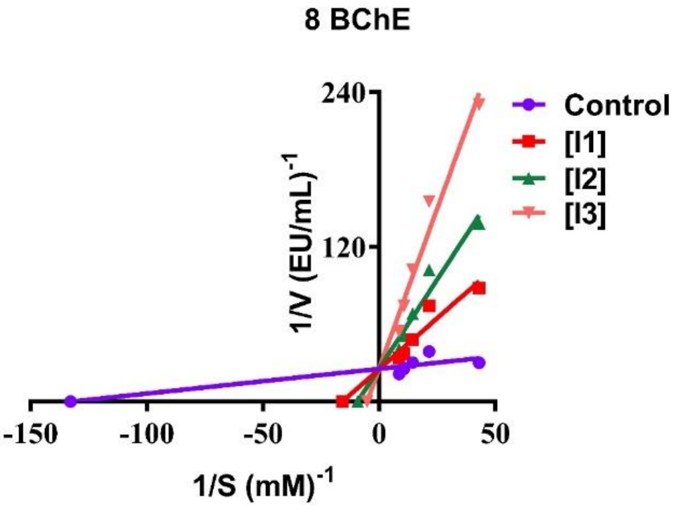

Figure S73. Lineweaver-Burk plots of compound 8 for BChE

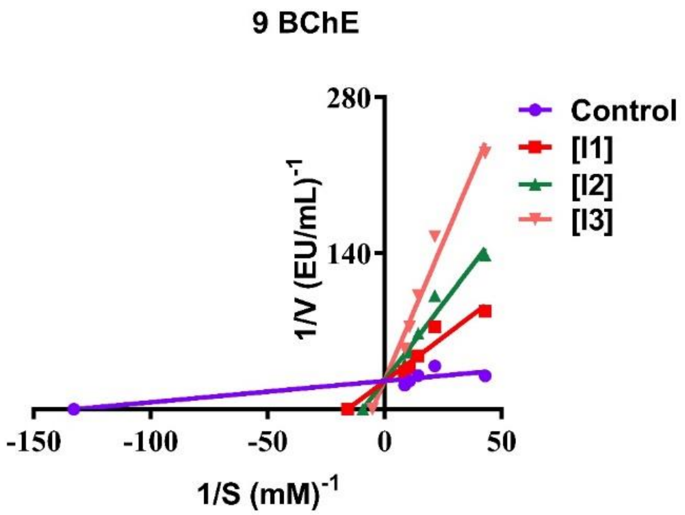

Figure S74. Lineweaver-Burk plots of compound 9 for BChE

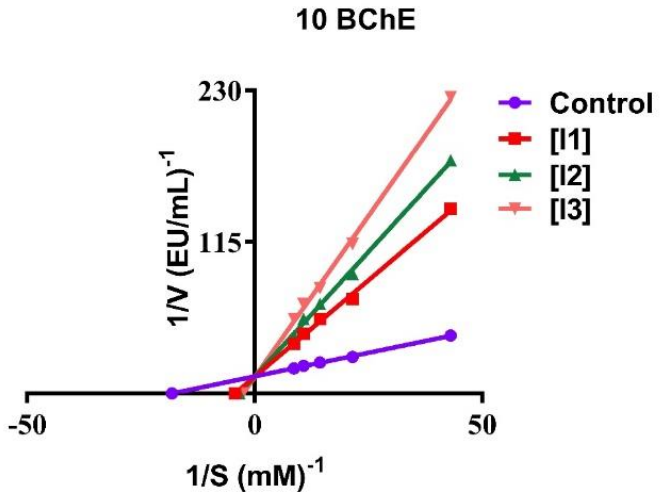

Figure S75. Lineweaver-Burk plots of compound 10 for BChE

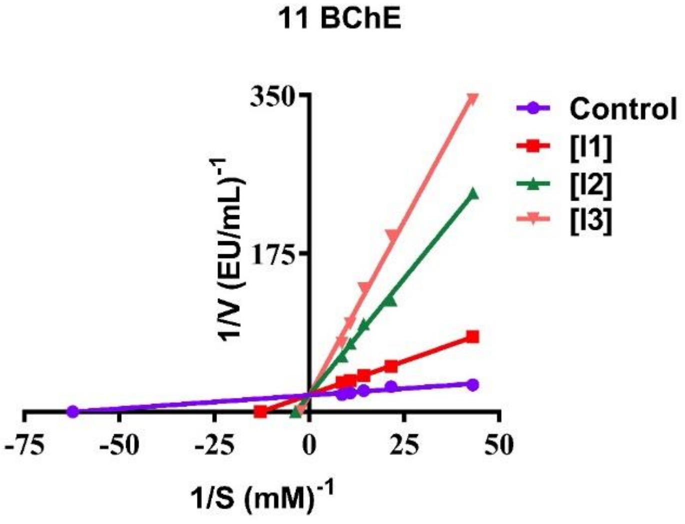

Figure S76. Lineweaver-Burk plots of compound 11 for BChE

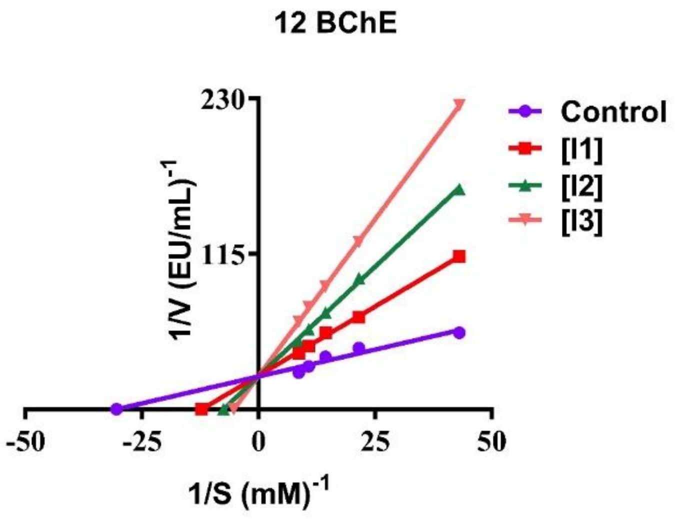

Figure S77. Lineweaver-Burk plots of compound 12 for BChE

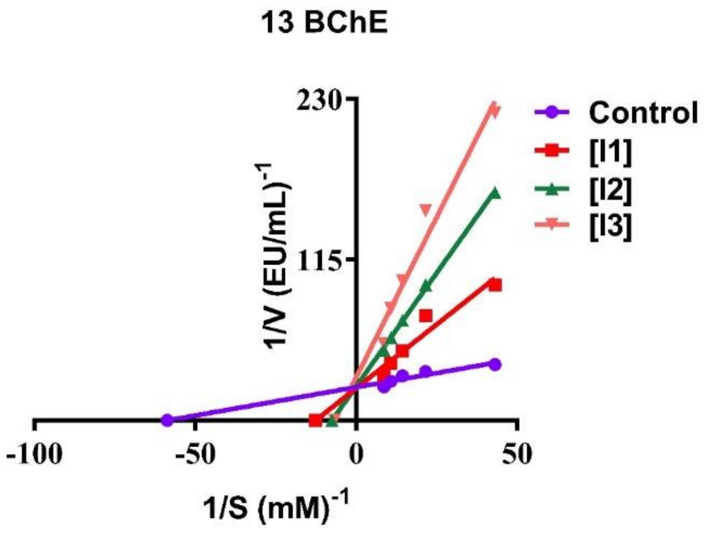

Figure S78. Lineweaver-Burk plots of compound 13 for BChE

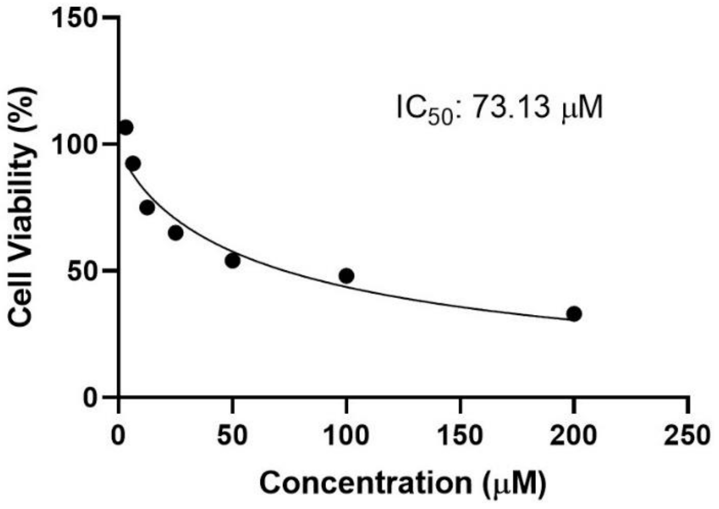

Figure S79. IC_{50} Graph of Compound 4 for HUVEC

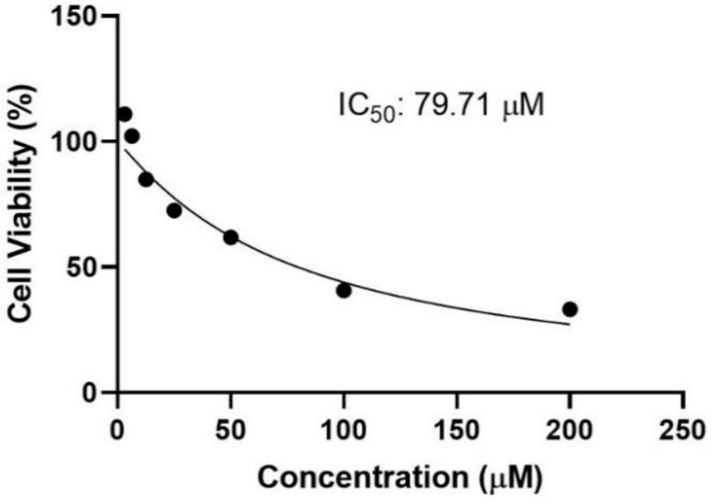

Figure S80. IC_{50} Graph of Compound 6 for HUVEC

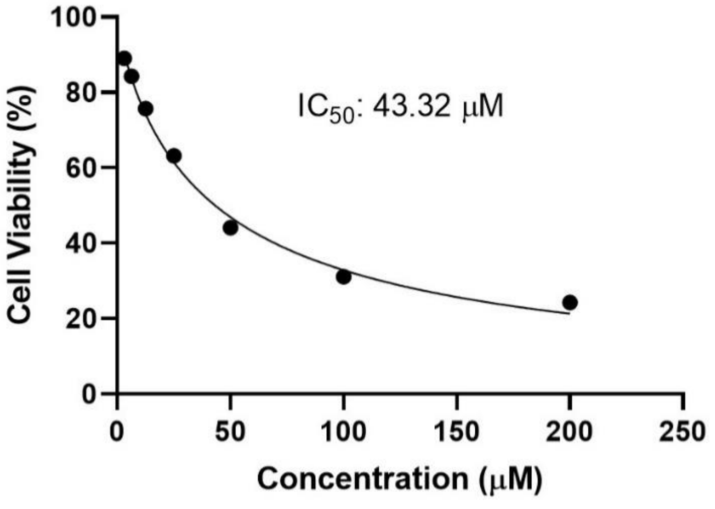

Figure S81. IC_{50} Graph of Compound 7 for HUVEC

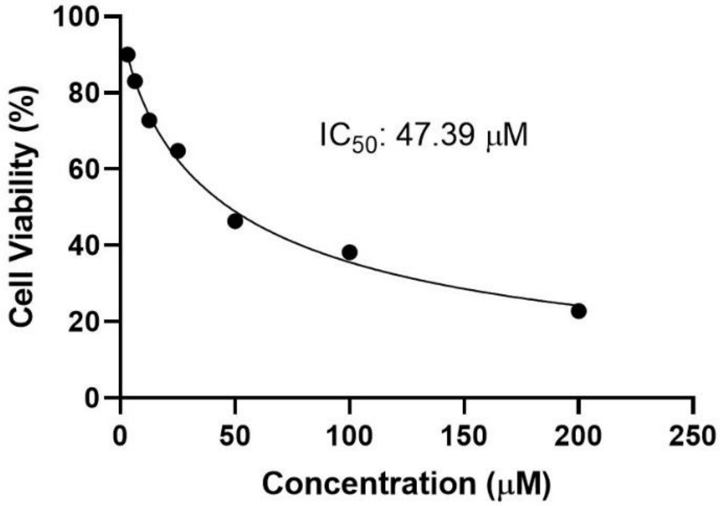

Figure S82. IC_{50} Graph of Compound 10 for HUVEC

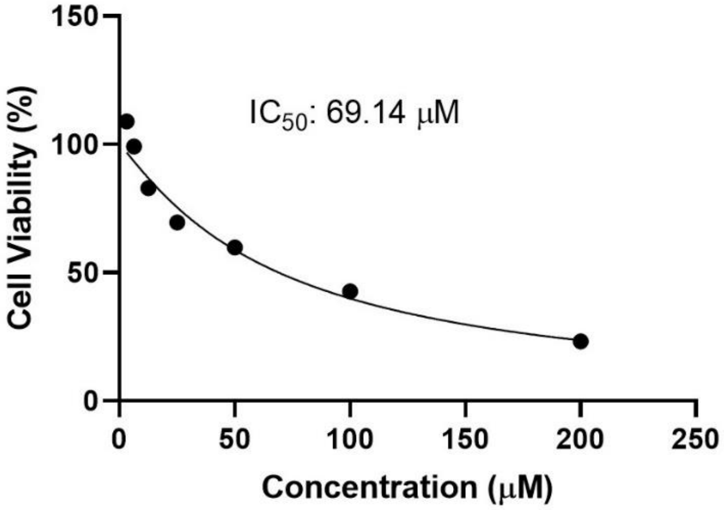

Figure S83. IC_{50} Graph of Compound 11 for HUVEC
